# Targeting of epigenetic co-dependencies enhances anti-AML efficacy of Menin inhibitor in AML with MLL1-r or mutant NPM1

**DOI:** 10.1038/s41408-023-00826-6

**Published:** 2023-04-13

**Authors:** Warren Fiskus, Christopher P. Mill, Christine Birdwell, John A. Davis, Kaberi Das, Steffen Boettcher, Tapan M. Kadia, Courtney D. DiNardo, Koichi Takahashi, Sanam Loghavi, Michael J. Soth, Tim Heffernan, Gerard M. McGeehan, Xinjia Ruan, Xiaoping Su, Christopher R. Vakoc, Naval Daver, Kapil N. Bhalla

**Affiliations:** 1 The University of Texas MD Anderson Cancer Center, Houston, TX USA; 2 University of Zurich and University Hospital Zurich, CH-8091 Zurich, Switzerland; 3 Syndax Pharmaceuticals, Waltham, MA USA; 4 Cold Spring Harbor Laboratory, Cold Spring Harbor, NY 11724 USA

**Keywords:** Acute myeloid leukaemia, Targeted therapies

## Abstract

Monotherapy with Menin inhibitor (MI), e.g., SNDX-5613, induces clinical remissions in patients with relapsed/refractory AML harboring MLL1-r or mtNPM1, but most patients either fail to respond or eventually relapse. Utilizing single-cell RNA-Seq, ChiP-Seq, ATAC-Seq, RNA-Seq, RPPA, and mass cytometry (CyTOF) analyses, present pre-clinical studies elucidate gene-expression correlates of MI efficacy in AML cells harboring MLL1-r or mtNPM1. Notably, MI-mediated genome-wide, concordant, log2 fold-perturbations in ATAC-Seq and RNA-Seq peaks were observed at the loci of MLL-FP target genes, with upregulation of mRNAs associated with AML differentiation. MI treatment also reduced the number of AML cells expressing the stem/progenitor cell signature. A protein domain-focused CRISPR-Cas9 screen in MLL1-r AML cells identified targetable co-dependencies with MI treatment, including BRD4, EP300, MOZ and KDM1A. Consistent with this, in vitro co-treatment with MI and BET, MOZ, LSD1 or CBP/p300 inhibitor induced synergistic loss of viability of AML cells with MLL1-r or mtNPM1. Co-treatment with MI and BET or CBP/p300 inhibitor also exerted significantly superior in vivo efficacy in xenograft models of AML with MLL1-r. These findings highlight novel, MI-based combinations that could prevent escape of AML stem/progenitor cells following MI monotherapy, which is responsible for therapy-refractory AML relapse.

## Introduction

Menin is a 610 amino acid scaffold protein encoded by the MEN1 gene [1, 2]. Menin binds to the Menin binding domains (MBD) in the N-terminus of MLL1 (KMT2A), which is a large (3696 amino acids) transcriptional regulator and a histone-lysine-*N*-methyltransferase [3–5]. The N-terminal 1400 amino acids of MLL1 constitute a transcription factor containing the MBD [3–5]. The C-terminus of MLL1 contains the SET domain acting as a histone methyltransferase that mediates histone H3 lysine 4 trimethylation [3–5]. The Menin-KMT2A complex regulates HOX gene clusters involved in embryonic development and hematopoiesis [2, 5–7]. HOXA9 and its co-factor MEIS1 are leukemogenic in myeloid stem progenitor cells [7–9]. HOXA9 functions as a pioneer factor and along with MEIS1 recruits CEBPα and MLL3/MLL4 to reprogram the enhancer landscape and promote leukemogenesis [8–11]. In MLL1-rearranged AML, the N-terminus of the MLL1 gene is fused to the C-terminus of any of over 80 fusion partners, including AF4, AF9, ENL, and ELL, which are part of and recruit the super elongation complex (SEC) (including AFF1/4 and pTEFb) and DOT1L to induce H3K4Me3 and H3K79Me2 marks on active chromatin [5, 10, 12, 13]. MLL1 fusion protein (MLL-FP) causes dysregulated expression of HOXA9, MEIS1, PBX3, MEF2C, and CDK6 [5, 12, 13]. Additionally, in AML with mutant (mt) NPM1 (NPM1c), MLL1 is the main oncogenic regulator of HOXA9, MEIS1 and FLT3, promoting self-renewal of myeloid progenitor cells [5, 14]. Conditional KO of MEN1 prevents MLL1-r AML [2, 5]. Treatment with orally bioavailable, investigational or clinical drug-candidate MI, disrupts binding of Menin to its binding pocket in MLL1/2 and MLL1-FP [3, 5]. This evicts Menin from the chromatin and reduces MLL1/2 and MLL1-FP binding to their targets, thereby repressing HOXA9, MEIS1, PBX3, MEF2C, FLT3, and CDK6 [5, 15–18]. Importantly, treatment with MI leads to differentiation and apoptosis of AML cells expressing MLL-FP or NPM1c [5, 15–20]. In early clinical trials, monotherapy with MI is well tolerated and has achieved objective remissions in patients with previously treated relapsed/refractory AML harboring MLL1-r or NPM1c, highlighting the promising on-target activity of MI on AML expressing MLL-FP or NPM1c [5, 21, 22]. However, most patients either fail to respond or eventually relapse [21, 22]. Therefore, there is a need to further interrogate the impact of MI treatment more comprehensively and concordantly on the epigenome and transcriptome, as well as on proteome, to elucidate molecular correlates of the biologic activity of MI. Additionally, it is also important to discover and test novel MI-based combinations that exhibit superior activity and prevent or abrogate MI-resistance. With this as the goal, we determined and highlight hitherto unreported effects of SNDX-50469, an orally bioavailable, investigational MI, concordantly altering accessible chromatin of active enhancers/promoters with the transcriptome and protein expressions in AML cell lines and patient-derived (PD) AML cells, including phenotypically defined AML stem/progenitor cells with MLL1-r or mtNPM1 [15, 16, 18]. We also present findings of a targeted, domain-specific, CRISPR-gRNA screen targeting epigenetic regulators. Based on the results of this screen, we present the synergistic in vitro activity of the MI-based combinations with novel agents that inhibit the “druggable” targets discovered by the screen. Specifically, our findings demonstrate that the combination of SNDX-50469 with BET protein, p300/CBP histone acetyltransferase (HAT), MOZ (KAT6A), or KDM1A (LSD1) inhibitor (LSD1i) exerts synergistic lethal activity against AML cell lines and PD AML cells harboring MLL-FP or NPM1c [23–26]. In addition, in immune-depleted mice engrafted with AML cell line or PD AML cells with MLL1-r with FLT3 mutation, compared to treatment with each agent alone co-treatment with SNDX-5613 (the clinical grade version of SNDX-50469) and pan-BET inhibitor OTX015 or p300/CBP inhibitor GNE-781 significantly reduced AML burden and improved overall survival of the mice [24, 27].

## Materials and methods

### Cell lines and cell culture

MOLM13 [DSMZ Cat# ACC-554, RRID: CVCL_2119], OCI-AML2 [DSMZ Cat# ACC-99, RRID:CVCL_1619) and OCI-AML3 [DSMZ Cat# ACC-582, RRID:CVCL_1844] cells were obtained from the DSMZ (Braunschweig, Germany). MV4–11 [ATCC Cat# CRL-9591, RRID:CVCL_0064] and THP-1 [ATCC Cat# TIB-202; RRID:CVCL_0006] cells were obtained from the ATCC (Manassas, VA). MOLM13 cells with isogenic TP53 mutations [R175H, R248Q and TP53-KO] were a gift from Dr. Benjamin L. Ebert (Dana Farber Cancer Center, Boston, MA). HEK-293T [RRID:CVCL_0063] cells were obtained from the Characterized Cell Line Core Facility at M.D. Anderson Cancer Center, Houston TX. All experiments with cell lines were performed within 6 months after thawing or obtaining from ATCC or DSMZ. MOLM13 and OCI-AML3 cells were cultured in RPMI-1640 media with 20% FBS, 1% penicillin/streptomycin and 1% non-essential amino acids. OCI-AML2 cells were cultured in MEM-alpha media with 20% FBS 1% penicillin/streptomycin and 1% non-essential amino acids. MV4–11 cells were cultured in ATCC-formulated IMDM media with 20% FBS, 1% penicillin/streptomycin, and 1% non-essential amino acids. HEK293T cells were cultured in high-glucose-formulated DMEM media with 10% FBS, 1% penicillin/streptomycin, and 1% glutamine. Logarithmically growing, mycoplasma-negative cells were utilized for all experiments. Following drug treatments, cells were washed free of the drug(s) prior to the performance of the studies described.

### Primary AML blasts

Patient-derived AML cells samples were obtained with informed consent as part of a clinical protocol approved by the Institutional Review Board of The University of Texas, M.D. Anderson Cancer Center. Mononuclear cells were purified by Ficoll Hypaque (Axis Shield, Oslo, Norway) density centrifugation following the manufacturer’s protocol. Mononuclear cells were washed once with sterile 1× PBS then suspended in complete RPMI media containing 20% FBS.

### Assessment of percentage non-viable cells

Following designated treatments (72–96 h), cultured cell lines or patient-derived (PD) AML blast cells, were washed with 1× PBS, stained with TO-PRO-3 iodide (Cat# T3605, Life Technologies, Carlsbad, CA) and analyzed by flow cytometry on a BD Accuri CFlow-6 flow cytometer (BD Biosciences, San Jose, CA).

### Statistical analysis

Significant differences between values obtained in AML cells treated with different experimental conditions compared to untreated control cells were determined using the Student’s *t* test in GraphPad V9. For the in vivo mouse models, a two-tailed, unpaired *t* test was utilized for comparing total bioluminescent flux. For survival analysis, a Kaplan–Meier plot and a Mantel–Cox log-rank test were utilized for comparisons of different cohorts. *p* Values of <0.05 were assigned significance.

## Results

### Knockout or MEN1 or degradation of Menin sensitizes AML cells with MLL1-r or mtNPM1 to BET or HAT inhibitor

**Fig. 1:**
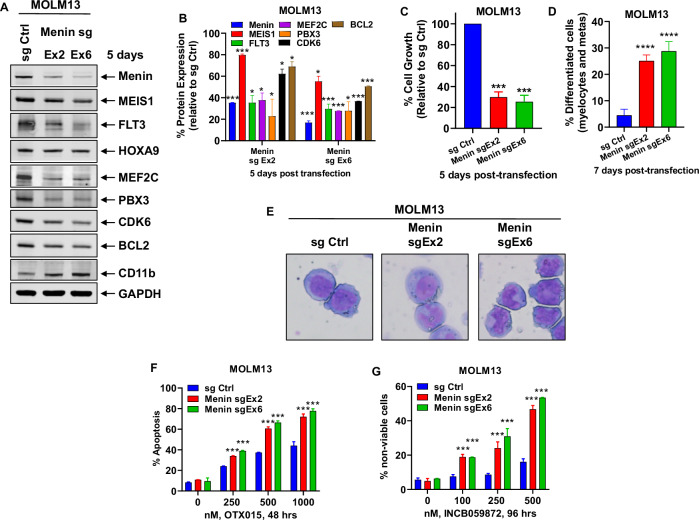
CRISPR-mediated depletion of Menin increases sensitivity to treatment with BET inhibitor or LSD1 inhibitor in AML cells. **A** MOLM13 cells were transfected with sgRNA against Exon 2 or Exon 6 of Menin or a negative control sgRNA (sg Ctrl) and incubated for 5 days. Then, total cell lysates were prepared and immunoblot analyses were conducted. The expression levels of GAPDH served as the loading control. Representative blots of two independent experiments are shown. **B** Densitometry analysis of Menin and MLL target gene immunoblots following sgRNA-mediated depletion of Menin in MOLM13 cells (from two independent experiments). *p < 0.05; ***p < 0.005 compared to sg Ctrl (determined by a two-tailed, unpaired *t* test). **C** MOLM13 cells were transfected with sg Ctrl or sg Menin Ex2 or Ex6 and incubated for 5 days. Total cell numbers in each condition (plated in duplicate) were counted using a Countess II automated cell counter. Mean of two independent experiments performed in duplicate ± S.D. ***p < 0.005 compared to sg Ctrl-transfected MOLM13 cells (determined by a two-tailed, unpaired *t* test). **D** MOLM13 were transfected with sg Ctrl or sg Menin Ex2 or Ex6 and incubated for 7 days. Cells were cytospun onto glass slides and stained with hematoxylin and eosin. The % of differentiated cells (myelocytes and metamyelocytes) were determined utilizing light microscopy. Columns, mean of two independent experiments performed in duplicate; Bars, Standard error of the mean (S.E.M). ****p < 0.001 compared to sg Ctrl-transfected MOLM13 cells (determined by a two-tailed, unpaired *t* test). **E** Representative images of sg Ctrl and sg Menin Ex2 or Ex6-induced morphologic differentiation in MOLM13 cells 7 days post-transfection. **F** MOLM13 cells were transfected with sg Ctrl or sg Menin Ex2 or Ex6 and incubated for 72 h. Then, cells were treated with the indicated concentrations of OTX015 for 48 h. The % of annexin V-positive, apoptotic cells were determined by flow cytometry. Mean of two independent experiments performed in duplicate ± S.D. ***p < 0.005 compared to sg Ctrl-transfected MOLM13 cells (determined by a two-tailed, unpaired *t* test). **G** MOLM13 cells were transfected with sg Ctrl or sg Menin Ex2 or Ex6 and incubated for 72 h. Following this, cells were treated with the indicated concentrations of INCB059872 for 96 h. The % of TO-PRO-3 iodide-positive, non-viable cells were determined by flow cytometry. Mean of two independent experiments performed in duplicate ± S.D. ***p < 0.005 compared to sg Ctrl-transfected MOLM13 cells (determined by a two-tailed, unpaired *t* test).

We first interrogated the CRISPR-gRNA dependency-screen (DepMap) to determine whether MEN1, the gene encoding Menin protein, is a dependency in AML cell lines. As shown in Fig. S1A, the MEN1 gene-effect scores were <0.5, highlighting it to be a dependency in several AML cell lines, including those that express MLL-FP (e.g., MOLM13, MV4–11, MonoMac-1 and MOLM14) or mtNPM1 (e.g., OCI-AML3) (Fig. S1A). We confirmed the dependency of MOLM13 cells on MEN1 by conducting a CRISPR-Cas9-mediated depletion of MEN1 via transfection of gRNAs targeting exon 2 or 6 of MEN1 in MOLM13 cells. Fig. S1B demonstrates that 5-days following transduction of either gRNA, there was significant repression of the mRNA of Menin, HOXA9, MEF2C, PBX3, MEIS1, JMJD1C, MYB, CDK6 and BCL2, but induction of the myeloid differentiation marker CD11b. Consistent with this, there was also a significant decline in the protein levels of MEIS1, MEF2C, PBX3, JMJD1C, MYB, CDK6 and BCL2, with up regulation of CD11b (Fig. 1A, B). This was associated with inhibition of in vitro growth of MOLM13 cells in suspension culture and increase in % of cells displaying morphologic features of differentiation (increase in % myelocytes and metamyelocytes) (Fig. 1C–E). We next determined the lethal effect of co-treatment with OTX015 (a pan-BET protein inhibitor) following gRNA mediated depletion of MEN1. This was undertaken because, by inhibiting the BET protein BRD4 activity and enhancer-driven expression of several AML relevant oncoproteins, e.g., MYC, CDK4/6 and BCL2, BET protein inhibitors were demonstrated to induce loss of viability in AML with MLL1-r or mtNPM1, and because BRD4 also transcriptionally regulates enhancer-driven expression of HOXA9-MEIS1 and their targets [20, 28–30]. Consistent with this, as shown in Fig. 1F, the CRISPR-mediated depletion of MEN1 sensitized MOLM13 cells to OTX015-induced apoptosis. Additionally, knockout of LSD1 (KDM1A) was previously documented to prevent the development of AML induced by MLL-AF9 [31]. Consistent with this, gRNA mediated depletion of MEN1 also increased cell death induced by co-treatment with the KDM1A inhibitor INCB059872 (Fig. 1G**)** [23]. Since perturbations in protein expressions, documented above, were noted 5 to 8 days after transduction of the gRNAs for CRISPR-Cas9-mediated MEN1 depletion, we employed the dTAG system to rapidly degrade MEN1-FKBP12(F36V) in MOLM13 and MV4–11 cells after sgRNA-mediated disruption of the endogenous MEN1 [18, 23, 32]. The dTAG system allows assessment of loss of Menin protein expression and downstream biological consequences within hours [32]. As shown in Fig. S2A, 24 h after adding dTAG-13, there was considerable decline in protein levels of Menin in MOLM13 and MV4–11 cells. Notably, as was observed following gRNA-mediated knockout of MEN1, in the dTAG13-treated MOLM13 and MV4–11 cells, there was a significant and dose-dependent increase in loss of viability due to treatment with OTX015 and INCB059872 (Fig. S2B–E).

### Effect of Menin inhibitor (MI) on chromatin accessibility, enhancer activity and transcriptome in AML cells

**Fig. 2:**
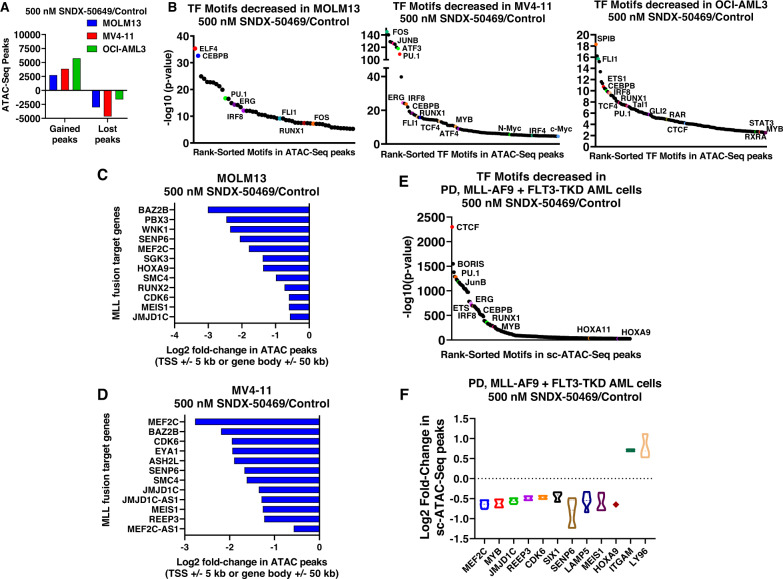
Treatment with Menin inhibitor depletes chromatin accessibility in MLL fusion target genes and Beta Catenin target genes in MLL-r AML and mtNPM1 expressing AML cells. **A** MV4-11, OCI-AML3, and MOLM13 cells were treated with the indicated concentrations of SNDX-50469 for 16 h. At the end of treatment, cells were used for ATAC-Seq analysis. Graph shows the average (of 2 replicates) of gained and lost transposase-accessible peaks. **B** Transcription factor binding motifs decreased in the lost ATAC peaks in MOLM13, OCI-AML3, and MV4–11 cells treated with 500 nM of SNDX-50469 for 16 h. **C**, **D** Log2 fold-change in chromatin accessibility (ATAC peaks) in MLL fusion target genes in MOLM13 and MV4–11 cells. **E** Transcription factor binding motifs decreased in the lost ATAC peaks in PD, MLL-AF9 + FLT3-TKD AML cells treated with 500 nM of SNDX-50469 for 16 h determined by single-cell ATAC-Seq analysis. **F** Log2 fold-change in chromatin accessibility (ATAC peaks) in MLL fusion target genes in PD, MLL-AF9 + FLT3-TKD AML cells as determined by single cell (sc) ATAC-Seq analysis.

Previous reports had highlighted that treatment with the MI SNDX-50469 repressed MLL1 or MLL-FP targets as well as induced in vitro differentiation and loss of viability of AML cells with MLL1-r or mtNPM1 [16, 18]. Here we determined the genome-wide effects of SNDX-50469 on chromatin accessibility with ATAC-Seq and gene-expression profiles with RNA-Seq in MOLM13, MV4–11 and OCI-AML3 cells [23]. Following SNDX-50469 treatment, ATAC-Seq analysis demonstrated large numbers of lost and gained peaks, representing alterations in accessibility of the chromatin (Fig. 2A). This was associated with loss of motifs for TFs involved in myelopoiesis, e.g., MYB, PU.1, IRF8, and RUNX1 (Fig. 2B). SNDX-50469 treatment caused log2 fold-change in ATAC-Seq peaks mapped to the *cis*-regulatory regions of the indicated MLL1-FP target genes in MOLM13 and MV4–11 cells (Fig. 2C, D). Also, in patient-derived AML cells harboring MLL-AF9 fusion with FLT3-TKD mutation, sc-ATAC-Seq analysis also demonstrated loss of binding sites of the same TFs and log2 fold-decline in sc-ATAC-Seq peaks in the MLL-FP target genes but increase in the ITGAM and LY96 genes (Fig. 2E, F). In OCI-AML3 cells, SNDX-50469 treatment perturbed the chromatin accessibility of several target gene loci. For example, the signal density plots of ATAC-Seq peaks show reduction in the peaks in the MEIS1, SENP6 and CDK6 loci in MOLM13 as well as MYB and TCF4 loci in OCI-AML3 cells (Fig. S3A–C). Overall, ATAC-Seq peaks declined in numerous RNA pol II transcribed loci (Fig. S3D). Utilizing ChIP-Seq analysis, by determining the signal-density of H3K27Ac occupancy, we next evaluated the effects of SNDX-50469 treatment, compared to the control, on active enhancers/promoters in MOLM13 cells. Following SNDX-50469 treatment, among the loci demonstrating log2 fold-decrease in peak density of H3K27Ac mark were the MLL1-FP target genes, including MEIS1, MEF2C, JMJD1C, SENP6, PBX3, LAMP5, and CDK6, as previously described [16, 18] (Fig. S3E).

**Fig. 3:**
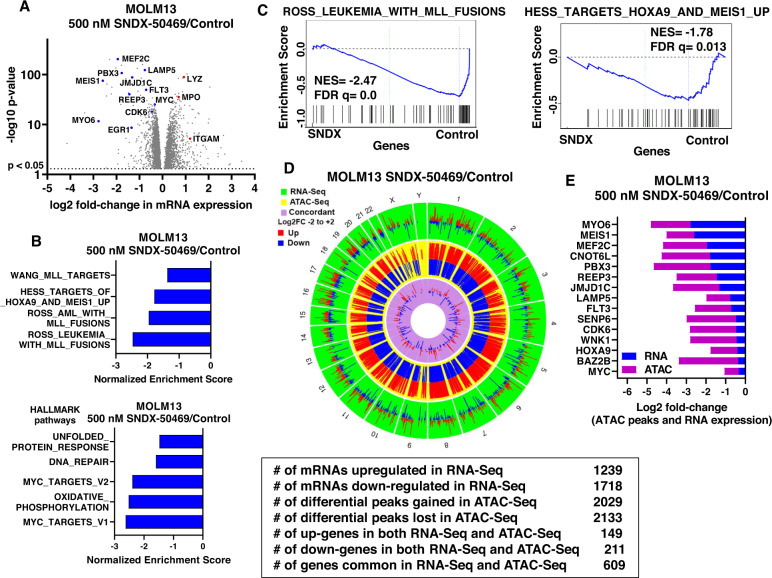
Treatment with Menin inhibitor concordantly depletes chromatin accessibility and mRNA expression in MLL-r AML cells. **A** Volcano plot of RNA-Seq-determined mRNA expression changes in MOLM13 cells treated with the indicated concentration of SNDX-50469 for 16 h. **B** Gene set enrichment analysis of SNDX-50469 treated MOLM13 cells compared to selected MLL-focused and HALLMARK data sets from MSIGDB. All q-values were less than 0.1. **C** Enrichment plots of SNDX-50469-treated MOLM13 cells with ROSS_LEUKEMIA_WITH_MLL_FUSIONS and HESS_TARGETS_HOXA9_AND_MEIS1_UP. **D**, **E** Circos plot and log2 fold-change of selected concordant ATAC-Seq and mRNA expression alterations in SNDX-50469-treated MOLM13 cells.

**Fig. 4:**
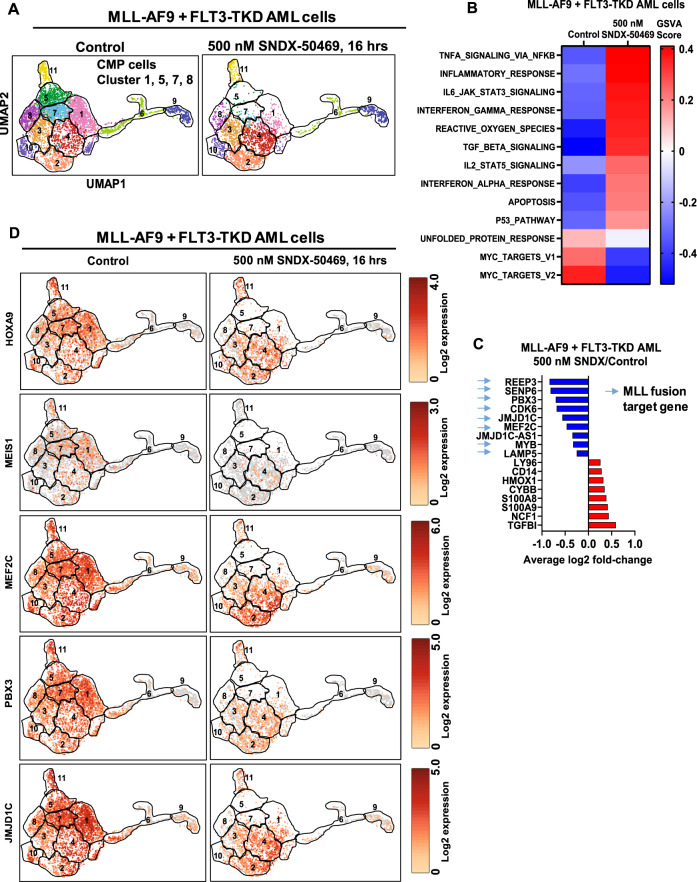
Menin inhibitor depletes MLL fusion target gene mRNA and induces differentiation associated mRNA expressions at the single-cell level in PD, MLL1-r AML cells. **A** UMAP plot of clustered gene expressions determined by single cell RNA-Seq analysis in PD, MLL-AF9 + FLT3-TKD cells treated with 500 nM of SNDX-50469 for 16 h. Based on similarities of cluster marker gene expressions, cells in cluster 1, 5, 7, and 8 were designated as myeloid progenitor cells utilizing the SingleR algorithm. The boundary of each cluster is outlined in black. **B** GSVA analysis of SNDX-50469-treated MLL-AF9 + FLT3-TKD AML mRNA expression changes against HALLMARK pathways. **C** Average log2 fold-change in significantly altered mRNAs in SNDX-50469-treated MLL-AF9 + FLT3-TKD AML cells determined by single-cell RNA-Seq analysis. **D** UMAP plot of selected MLL1 fusion target gene expressions at the single cell level in SNDX-50469-treated MLL-AF9 + FLT3-TKD AML cells.

We next determined the effects of SNDX-50469 treatment on mRNA expressions utilizing RNA-Seq analysis. Figure S4A demonstrates the heat map of mRNAs that are up- or down-regulated by SNDX-50469 treatment in MOLM13 cells, with log2 fold-decline in mRNA expressions of MEIS1, HOXA9, JMJD1C, PBX3, SENP6 and BMI but upregulation of mRNAs of the differentiation markers ITGAM and LYZ (Figs. 3A and S4B). Gene set enrichment analyses (GSEA) (compared to the GO and HALLMARK pathways) of the perturbed mRNAs due to SNDX-50469 treatment demonstrated significant negative normalized enrichment scores (NES) for gene-sets involved in MLL1 and MLL-FP targets, HOXA9 and MEIS1 targets, unfolded protein response (UPR), DNA repair, MYC targets and oxidative phosphorylation genes (Fig. 3B, C). Similar effects on mRNA expressions and their gene-sets were also observed following SNDX-50469 treatment of MV4–11 and OCI-AML3 cells (Fig. S4C–F). Next, we evaluated SNDX-50469-mediated genome-wide, concordant, log2 fold-perturbations (up or down-regulation) in ATAC-Seq and RNA-Seq peaks in MOLM13 and OCI-AML3 cells (Figs. 3D and S4G). As shown in Figs. 3E and S4H, specific peak reductions were observed at the loci of MLL-FP target genes, including MEIS1, MEF2C, FLT3, PBX3, CDK6, HOXA9 and LAMP5. The Venn diagrams in Fig. S5A, B demonstrate that, following SNDX-50469 treatment, 200 and 99 mRNAs were commonly upregulated or down regulated, respectively, in OCI-AML3 and MOLM13 cells. Among the commonly down-regulated mRNAs were those of MEIS1, FLT3, LAMP5, MYC, CDK6 and EGR1, whereas the mRNAs commonly induced were those of ITGAM, CD86 and ITGAX (Fig. S5C, D). We next utilized scRNA-Seq analysis on a patient-derived (PD) bone marrow aspirate (BMA) sample harboring MLL1-FP and FLT3-TKD (~6000 cells sampled). The UMAP (Uniform Manifold Approximation and Projection plot) revealed that SNDX-50469 treatment diminished cell events and gene expression in the CMP (common myeloid progenitor) clusters (# 1, 5, 7, and 8) (Figs. 4A and S6A). GSVA of the scRNA-Seq-determined mRNA expressions demonstrated that, following SNDX-50469 treatment, GSVA scores increased for the gene-sets of TNFα signaling via NFkB, inflammatory response, IL6-JAK-STAT signaling, interferon α and γ responses, reactive oxygen species, TGFβ signaling and apoptosis, whereas GSVA scores declined for MYC targets (Fig. 4B). SNDX-50469 treatment also caused log2 fold-reduction in the main MLL-FP target genes (Fig. 4C). Notably, these genes, including HOXA9, MEIS1, FLT3, LAMP5, SENP6, MEF2C, CDK6, PBX3 and JMJD1C were especially reduced in the CMP clusters of cells (Figs. 4D and S6B). Additionally, SNDX-50469 treatment reduced the number of cells expressing a stem/progenitor cell signature (Single-R) (Fig. S6C) [33]. Next, we determined the effects of SNDX-50469 treatment on protein expressions via RPPA (reversed phase protein arrays) in the PD AML cells harboring MLL1-FP and FLT3-TKD. Figure S6D shows the heat map of protein expressions up (92 proteins) or down regulated (130 proteins), following exposure to SNDX-50469 for 48 h. Whereas log2 fold-decline in the protein expressions of p-4EBP1, p-mTOR, pRB, pS6, SGK3, p-BAD, BCL2 and XIAP was observed, the levels of cleaved caspase 3, 7, 8, BIM and p-H2AX increased (Fig. S6E).

### Targetable dependencies identified by protein domain-specific CRISPR-Cas9 screen and synergistic lethality due to SNDX-50469 and BET inhibitor

**Fig. 5:**
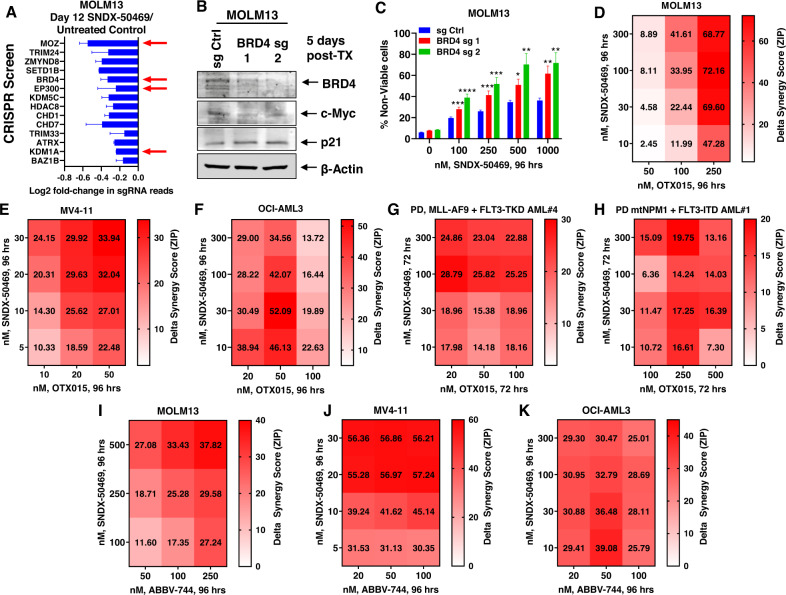
Co-treatment with Menin inhibitor and BETi OTX015 induces synergistic in vitro lethality in MLL1-r and mtNPM1-expressing AML cells. **A** MOLM13-Cas9 expressing cells were transduced (biologic replicates) with a library of domain-specific sgRNAs against chromatin modifying proteins and incubated for 8 days. Then, 500 nM of SNDX-50469 was added and the cells were incubated for an additional 96 h. Live cells were harvested; genomic DNA was isolated and minimally amplified with primers flanking the sgRNA sequences. Sequencing libraries were generated and amplicon-seq was performed. The graph shows log2 fold-change in sgRNAs which dropped out significantly (*p* < 0.05 and f.d.r. <0.05) more due to treatment with SNDX-50469 than control cells at day 12 post-transduction in both replicates. **B** MOLM13 cells were transfected with RNPs containing sg Ctrl or BRD4 sgRNAs and incubated for 5 days. At the end of treatment, cells were harvested and immunoblot analysis was conducted as indicated. The expression levels of β-Actin in the lysates served as the loading control. **C** MOLM13 cells were transfected with RNPs containing sg Ctrl or BRD4 sgRNAs and incubated for 3 days. Then, cells were treated with the indicated concentrations of SNDX-50469 for 96 h. The % of TO-PRO-3 iodide-positive, non-viable cells were determined by flow cytometry. Columns, mean of three independent experiments; Bars, S.E.M. *p < 0.05, *p* < 0.01, ***p < 0.005 and ****p < 0.001 compared to sg Ctrl transfected cells treated with SNDX-50469 for 96 h (determined by a two-tailed, unpaired *t* test in GraphPad V9). **D**–**F** MOLM13, MV4–11 and OCI-AML3 cells were treated with the indicated concentrations of SNDX-50469 and/or OTX015 for 96 h. At the end of treatment, the % non-viable cells were determined by staining with TO-PRO-3 iodide and flow cytometry analysis. Delta synergy scores were determined by the ZIP method within the SynergyFinder V2.0 web application. Synergy scores >1.0 indicate a synergistic interaction of the two agents in the combination. **G**, **H** Patient-derived AML cells with MLL1-r and FLT3-TKD (#4) or mtNPM1 and FLT3-ITD (#1) were treated with the indicated concentrations of SNDX-50469 and/or OTX015 for 72 h. At the end of treatment, the % non-viable cells were determined by staining with TO-PRO-3 iodide and flow cytometry analysis. Delta synergy scores were determined by the ZIP method within the SynergyFinder V2.0 web application. Synergy scores >1.0 indicate a synergistic interaction of the two agents in the combination. **I**–**K** MV4–11, OCI-AML3, and MOLM13 cells were treated with the indicated concentrations of SNDX-50469 and/or ABBV-744 for 96 h. At the end of treatment, the % non-viable cells were determined by staining with TO-PRO-3 iodide and flow cytometry analysis. Delta synergy scores were determined by the ZIP method within the SynergyFinder V2.0 web application. Synergy scores >1.0 indicate a synergistic interaction of the two agents in the combination.

We next conducted a protein domain-focused CRISPR-Cas9 screen to nominate potential drug targets in SNDX-50469-treated and untreated MOLM13 cells [23, 34]. For this, we utilized a previously validated GFP-tagged gRNA library (1390 gRNAs and ~8 gRNA per gene) targeting chromatin regulators, which were transduced into MOLM13 and MV4–11 cells that stably expressed Cas9. Eight days after transduction of gRNAs, cells were treated or untreated with SNDX-50469 (500 nM) for 4 days, following which sequencing libraries were constructed and NGS (Next Generation Sequencing) was performed to determine negative selection of the gRNAs found on day-12 in SNDX-50469-treated versus untreated MOLM13 and MV4–11 cells, compared to the gRNA profile sequenced on day-2 following transduction of gRNAs. Figure S7A, B demonstrate that there was a log2 fold-decline in gRNA reads in untreated MOLM13 and MV4–11 cells targeting specific genes on day-12 compared to day-2, including MOZ BRD4, DOT1L, EP300 and KDM1A. Comparing the day-12 gRNA reads in untreated and SNDX-50469-treated MOLM13 cells also showed greater dropouts (log2 fold-decline) of BRD4, EP300, MOZ and KDM1A gRNAs, indicating these targets to be co-dependencies with SNDX-50469 treatment (Fig. 5A). By transfecting two separate gRNAs, we next knocked out BRD4 in MOLM13 cells, which depleted BRD4 and c-Myc protein levels, but increased p21 levels, as previously described (Fig. 5B) [25]. Notably, knockout of BRD4 sensitized MOLM13 cells to SNDX-50469-induced loss of viability in a dose-dependent manner (Fig. 5C). We next determined in vitro synergistic activity of co-treatment with SNDX-50469 and the pan-BET protein inhibitor OTX015 against AML cell lines and PD AML cells expressing MLL-FP or NPM1c. Treatment with this combination exerted synergistic lethality against MOLM13, MV4–11 and OCI-AML3 cells, with delta synergy scores by the ZIP method of greater than 2 (Figs. S7C–E and 5D–F). This synergistic lethality due to the combination was observed in MOLM13 cells despite low levels of lethal activity of either SNDX-50469 or OTX015 alone (Fig. S7C). Similar synergistic lethality with the two agents was also observed in THP1 and OCI-AML2 cells, which also harbor MLL1-r (Fig. S7F, G). As observed in cultured cell lines, co-treatment with SNDX-50469 and OTX015 also displayed synergistic lethal activity against PD AML cells harboring MLL-AF9 plus FLT3-TKD (sample #4, in the oncoplot in Fig. S8A) or mtNPM1 plus FLT3-ITD (Figs. 5G, H and S8B, C). Synergistic lethal activity of co-treatment with SNDX-50469 and OTX015 was also observed in 3 samples each of PD MLL1-r or mtNPM1 (Fig. S8D–J). Additionally, co-treatment with SNDX-50469 and the BD2 (bromodomain 2)-selective BET inhibitor ABBV-744 was also synergistically lethal against MV4–11, MOLM13 and OCI-AML3 cells (Figs. 5I–K and S9A–C) [35]. Approximately 9% of MLL1-r AML also exhibits a co-mutation in TP53, which is known to confer therapy resistance and poor outcome in AML [18, 36–39]. We had previously reported that treatment with SNDX-50469 exerted the same but modest lethal effect in MOLM13 versus CRISPR-edited MOLM13 cells with mono-allelic knock-in of TP53-R175H or TP53-R248Q mutation [18, 40]. Here, we determined that, although OTX015 monotherapy exerted modest lethal effects, co-treatment with SNDX-50469 and OTX015 induced synergistic lethal effects in the CRISPR-edited MOLM13 cells with mono-allelic TP53-R175H or TP53-R248Q mutation (Fig. S10A, B). Additionally, co-treatment with SNDX-50469 and ABBV-744 was also synergistically lethal against MOLM13 cells with the knock-in of TP53 mutations (Fig. S10C, D).

Based on the additional druggable co-dependencies revealed by the CRISPR screen, e.g., EP300, KDM1A (LSD1) and MOZ, we next evaluated the synergistic in vitro lethal activity of co-treatment with SNDX-50469 and GNE-049 or GNE-781 (CBP/p300 inhibitors), INCB059872 (KDM1A inhibitor) or WM1119 (MOZ inhibitor). The combination of SNDX-50469 and GNE-049 synergistically induced loss of viability in MV4–11, OCI-AML3, OCIAML2, MOLM13 as well as the CRISPR-edited MOLM13 cells with the knock-in of TP53 mutations (Fig. S11A–F). This combination also exerted synergistic lethality against PD AML cells with MLL1-r (2 samples) or with mtNPM1 (2 samples) (Fig. S11G–K). It is noteworthy that treatment with SNDX-50469 and GNE-049 or OTX015 induced loss of viability in less than 20% (over untreated control) normal CD34 + progenitor cells (Fig. S11L, M). Co-treatment with SNDX-50469 and GNE-781 was also synergistically lethal against MOLM13, MV4–11 and OCI-AML3 (Fig. S12A–C). Also, co-treatment with SNDX-50469 and the MOZ inhibitor WM1119 was synergistically lethal against MV4–11 and MOLM13 cells (Fig. S12D, E). The combination of SNDX-50469 and the KDM1A inhibitor INCB059872 also exerted synergistic lethality against MOLM13, MV4–11, OCI-AML3, as well as against PD AML cells with MLL1-r or mtNPM1 (Fig. S13A–G). Overall, these results validate findings of the CRISPR screen by demonstrating that combined targeting with SNDX-50469 and agents that target CBP/EP300, MOZ or KDM1A, versus each agent alone, exerts greater lethal effects against AML cells with MLL1-r or mtNPM1.

### Molecular correlates of superior efficacy of co-treatment with Menin inhibitor and BET inhibitor against AML cells

**Fig. 6:**
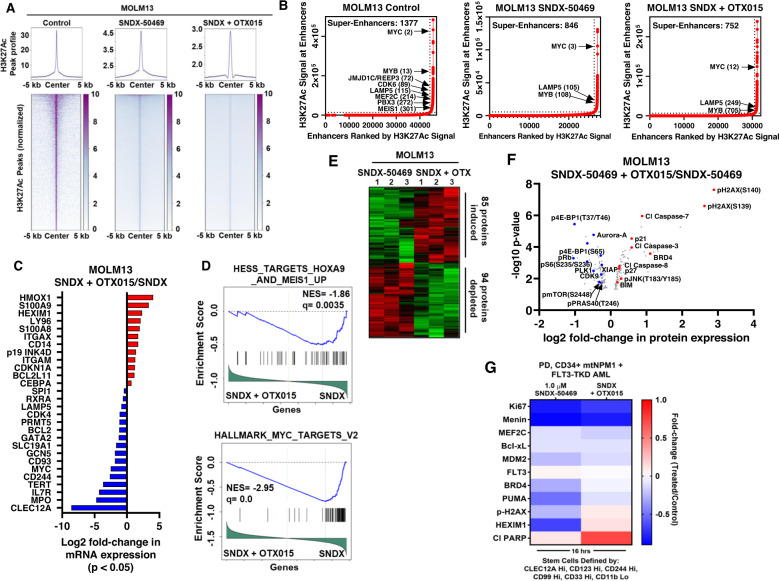
Compared to SNDX-50469 alone, co-treatment with SNDX-50469 and OTX015 further reduces H3K27Ac occupancy on chromatin, alters the super enhancer landscape, transcriptome and proteome in MLL1-r MOLM13 cells and PD, mtNPM1 expressing AML cells. **A** MOLM13 cells were treated with 500 nM of SNDX-50469 or SNDX-50469 plus 500 nM of OTX015 for 16 h. H3K27Ac ChIP-Seq analysis was conducted. Tag density plots and heat maps of H3K27Ac peaks genome-wide are shown for each treatment. **B** Ranked ordering of super enhancers (ROSE) analysis in MOLM13 cells treated with SNDX-50469 alone or in combination with OTX015, as described in **A**. **C** MOLM13 cells were treated with 500 nM of SNDX-50469 and/or 500 nM of OTX015 for 16 h. RNA-Seq analysis was performed. Log2 fold-changes in selected, significantly altered (p < 0.05) leukemia relevant genes are shown. **D** Enrichment plots comparing the mRNA expression signature of SNDX-50469 + OTX015 over SNDX-50469 to HESS_TARGETS_HOXA9_AND_MEIS1_UP and HALLMARK_MYC_TARGETS_V2 gene sets. **E**, **F** MOLM13 cells were treated in biologic triplicates with 500 nM of SNDX-50469 alone for 48 h or with OTX015 added in the last 24 h of incubation. Cells were harvested and reverse phase protein array analysis was conducted. The heat map shows the total number of significantly (p < 0.05) up and down-regulated proteins in each cell sample due to combination treatment over single agent SNDX-50469 treatment. The volcano plot highlights the most significantly altered proteins in each cell sample due to combination treatment over single agent SNDX-50469 treatment. **G** Patient-derived AML cells were treated with 1000 nM of SNDX-50469 alone, and in combination with 500 nM of OTX015 for 16 h. Cells were harvested and analyzed by CyTOF analysis utilizing a cocktail of rare metal element-tagged antibodies. Leukemia stem cells were defined by high expression of CLEC12A, CD123, CD244, CD99 and CD33 but low expression of CD11b. Heat map shows the absolute fold change of significantly altered protein expressions in the treated over control for each sample.

Next, we further probed the superior efficacy of co-treatment with SNDX-50469 and OTX015 versus SNDX-50469 alone, by comparing their effects on the active enhancers and gene expressions in MOLM13 and PD AML cells with mtNPM1. Utilizing H3K27Ac ChIP-Seq analysis, heatmaps of H3K27Ac peaks at enhancers and promoters, genome-wide, showed a greater reduction in the peaks following treatment with SNDX-50469 and OTX015 compared to SNDX-50469 alone (Fig. 6A). As shown in the peak density plots, treatment of MOLM13 cells with SNDX-50469 and OTX015, compared to control or SNDX-50469 alone [18] caused marked log2 fold-reduction in the H3K27Ac peaks at the enhancers of MEIS1, MEF2C, CDK6 and FLT3 (Fig. S14A–D). Compared to untreated control MOLM13, or MOLM13 cells treated with SNDX-50469 alone, treatment with SNDX-50469 plus OTX015 reduced the rank-ordering of the super enhancers (ROSE), including those of MYC, MYB and LAMP5 genes and loss of MLL-FP target genes, including MEF2C, PBX3, CDK6, JMJD1C/REEP3 and MEIS1 (Fig. 6B). RNA-Seq analysis showed that, consistent with greater inhibition of the active chromatin at super-enhancers and enhancer-promoters, there were greater log2 fold-perturbations in the mRNA expressions of numerous genes, including LAMP5, CDK4, BCL2, GATA2, GCN5, MYC, CD93, CD244, TERT, IL7R (IL7 receptor), SLC19A1 (folate transporter, a target of c-Myc), MPO and CLEC12A, but log2 fold-increase in HMOX1, S100A8/9, HEXIM1, ITGAM, CDKN1A, and BCL2L11 in MOLM13 cells (Figs. 6C and S14E). Gene set enrichment analyses (GSEA) of the RNA-Seq-determined, perturbed mRNAs due to SNDX-50469 plus OTX015, compared to SNDX-50469 treatment alone in MOLM13 cells demonstrated significant, negative enrichment for target gene-set of HOXA9-MEIS1 and MYC (Fig. 6D), as well as mTORC1 signaling, mRNA processing, oxidative phosphorylation, ribosome biogenesis, translation initiation, and translation elongation (Fig. S14F). However, gene sets with significant, positive enrichment included those involved in TNFα signaling via NFkB, interferon α and inflammatory responses, apoptosis, TGFβ signaling, stem cell differentiation, CEBPα targets and TP53 pathway (Fig. S14F). Utilizing RPPA, we next determined the effect of SNDX-50469 plus OTX015, compared to SNDX-50469 treatment alone, on protein expressions in MOLM13 cells. Figure 6E demonstrates the heat map of perturbations in protein levels by the RPPA analysis, showing increase in 85 and reduction in 94 protein expressions, following treatment of MOLM13 cells with SNDX-50469 plus OTX015 over SNDX-50469 alone for 48 h. Among the proteins exhibiting a log2 fold-reduction in levels were p-PRAS40, p-mTOR, p-4EBP1, p-S6 and PLK4, all involved in protein translation, as well as XIAP, CDK9, Aurora A and p-RB (Fig. 6F). In contrast, log2 fold-increase in protein levels of BRD4, p21, p-H2AX, p-JNK, BIM and cleaved caspase 3/7 were observed (Fig. 6F). We also utilized CyTOF analysis to determine protein expression perturbations in PD, CD34 + , phenotypically characterized, AML stem-progenitor cells with mtNPM1 and FLT3-TKD, i.e., exhibiting high expressions of CLEC12A, CD123, CD244, CD90 and CD33, but low expression of CD11b, following treatment with SNDX-50469 plus OTX015 compared to SNDX-50469 alone [41–43]. Figure 6G demonstrates that treatment with SNDX-50469 plus OTX015 compared to SNDX-50469 alone for 16 h, while resulting in decline in the protein levels of Ki67, Menin and FLT3, upregulated expressions of p-H2AX, HDM2, Puma, HEXIM1 and cleaved PARP involved in growth inhibition and apoptosis of AML cells [44–46]. These findings also suggest that co-treatment with SNDX-50469 and OTX015 exerts potentially greater lethal activity against PD AML stem-progenitor cells.

### Superior in vivo efficacy of combined therapy with Menin inhibitor and BET or CBP/p300 inhibitor against AML cells

**Fig. 7:**
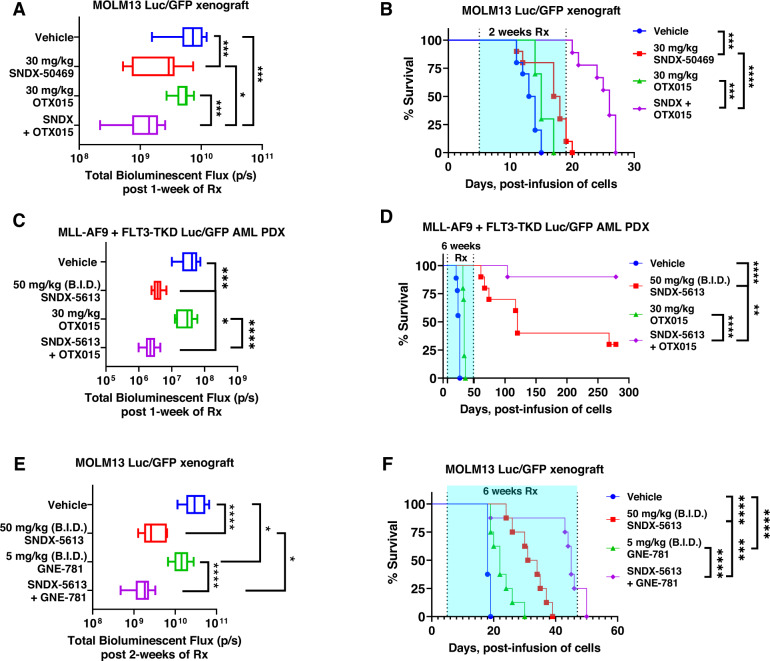
Treatment with Menin inhibitor-based combinations reduces leukemia burden and significantly improves median and overall survival of NSG mice bearing MLL-r AML xenografts. **A** Total photon counts [flux] (determined by bioluminescent imaging) in NSG mice (*n* = 10 per cohort) engrafted with MOLM13 Luc/GFP cells and treated for 1 week with SNDX-50469 and/or OTX015 at the indicated doses. **B** Kaplan–Meier survival plot of NSG mice engrafted with MOLM13 Luc/GFP cells and treated with SNDX-50469 (30 mg/kg, daily × 5 days, P.O.) and/or 30 mg/kg of OTX015 (daily × 5 days, P.O.) for 2 weeks. Significance was calculated by a Mantel–Cox log-rank test. **C** Total photon counts [flux] (determined by bioluminescent imaging) in NSG mice (*n* = 10 per cohort) engrafted with PD, MLL-AF9 + FLT3-TKD Luc/GFP AML cells and treated with vehicle, SNDX-5613 and/or OTX015 at the indicated doses for 1 week. **D** Kaplan–Meier survival plot of NSG mice engrafted with PD, MLL-AF9 + FLT3-TKD Luc/GFP AML cells and treated with SNDX-5613 (50 mg/kg B.I.D. × 5 days, P.O.) and/or OTX015 (30 mg/kg, daily × 5 days, P.O.) for 6 weeks. Significance was calculated by a Mantel–Cox log-rank test. *p < 0.05, **p < 0.01, ***p < 0.005, ****p < 0.001. **E** Total photon counts [flux] (determined by bioluminescent imaging) in NSG mice (*n* = 10 per cohort) engrafted with MOLM13 Luc/GFP cells and treated for 2 weeks with vehicle, SNDX-5613 and/or GNE-781 at the indicated doses. *p < 0.05, ****p < 0.001 determined by a two-tailed, unpaired *t* test in GraphPad V9. **F** Kaplan–Meier survival plot of NSG mice engrafted with MOLM13 Luc/GFP cells and treated with SNDX-5613 (50 mg/kg B.I.D. × 5 days, P.O.) and/or 5 mg/kg of GNE-781 (B.I.D. × 5 days, P.O.) for 6 weeks. Significance was calculated by a Mantel–Cox log-rank test. ***p < 0.005, ****p < 0.001 compared to vehicle or either single agent.

We next determined the in vivo anti-leukemia efficacy of SNDX-50469 or SNDX-5613 and/or OTX015 in an aggressive and lethal MOLM13 xenograft model or in an AML PDX (PD xenograft) model expressing MLL-AF9 and FLT3-TKD, both models transduced with Luciferase/GFP for bioluminescence imaging. Following tail-vein infusion and engraftment of MOLM13 cells, cohorts of mice were treated with vehicle control, or with SNDX-50469, or OTX015 alone, or co-treatment with SNDX-50469 and OTX015. The dose of each drug employed here was previously determined to be safe [18, 23, 47]. Whereas monotherapy with OTX015 alone for one week exhibited modest reduction of AML burden, treatment with SNDX-50469 alone induced marked and significant reduction in AML burden (Fig. 7A). Moreover, co-treatment with SNDX-50469 and OTX015 was significantly superior in reducing the AML burden compared to vehicle or each drug alone (Fig. 7A). Consistent with this, co-treatment with SNDX-50469 and OTX015 for 2 weeks was significantly superior in improving the median and overall survival of the mice, as compared to treatment with each drug alone or with vehicle control (Fig. 7B). In NSG mice engrafted with PD AML cells harboring MLL1-r (MLL-AF9) and FLT3-TKD, combined therapy with SNDX-5613 and OTX015 for one or for 6 weeks, as compared to each drug alone, was also significantly superior in reducing the AML burden, as well as significantly improving survival of the mice, respectively (Fig. 7C, D). Indeed, co-treatment with SNDX-5613 and OTX015 yielded a plateau in the survival curve for up to 280 days with 80% of mice in the cohort surviving, compared to 30% of the cohort treated with SNDX-5613 alone (*p* < 0.01) (Fig. 7D). Neither monotherapy with the agents nor treatment with the combinations was associated with weight loss nor other toxicities, as compared to mice treated with vehicle alone. We next evaluated the in vivo efficacy of SNDX-5613 and GNE-781 versus each drug alone or vehicle control in NSG mice tail-vein engrafted with MOLM13 cells harboring MLL1-r (MLL-AF9) and FLT3-ITD. Whereas SNDX-5613 more so than GNE-781 significantly reduced AML burden, treatment with SNDX-5613 and GNE-781 for 2-weeks was significantly superior in reducing the AML burden compared to each drug alone or vehicle control alone (*p* < 0.05) (Fig. 7E). Co-treatment with SNDX-5613 and GNE-781 for 6-weeks was significantly superior to either SNDX-5613 or GNE-781 alone in improving median and overall survival of the mice without inflicting weight loss or other toxicities (Fig. 7F). These findings indicate that co-treatment with SNDX-50469 or SNDX-5613 and OTX015 or GNE-781 are effective combination therapies worthy of further in vivo testing and development in AML with MLL1-r and mtNPM1.

## Discussion

Utilizing ATAC-Seq and RNA-Seq, our studies profile for the first time the concordant perturbations, genome-wide, in chromatin accessibility and mRNA expressions, following treatment with Menin inhibitor. In MLL1-r AML cells, they show a decline in ATAC-Seq and RNA-Seq peak densities in MLL1/MLL-FP-target genes, including HOXA9, MEIS1, MYB and MYC, and their targets. Reduction in ATAC-Seq peaks at several of these loci, representing reduced chromatin accessibility, was associated with reduced H3K27Ac occupancy, indicating repression of active regulatory chromatin of Menin-MLL1 targets, including MEIS1, PBX3, MEF2C, JMJD1C, SENP6 and CDK6 [16, 18, 19]. Our findings also highlight the RNA-Seq-determined, perturbed mRNA expressions which were common between SNDX-50469-treated AML cells with MLL1-r (MOLM13) and mtNPM1 (OCI-AML3). These included repression of MLL1/MLL-FP targets as well as induction of AML differentiation-associated gene expressions. Furthermore, data from scRNA-Seq conducted on PD BMA sample of AML with MLL1-r and FLT3-TKD confirm that treatment with MI repressed cell events and MLL-FP target genes in CMP cell-clusters. Importantly, this was associated with reduced numbers of cells expressing the stem/progenitor cell signature [42, 43, 48]. This anti-AML activity of MI correlated with positive enrichment of mRNA gene-sets belonging to TNFα signaling via NFκB, inflammatory response, IL6-JAK-STAT signaling, interferon α and γ responses, reactive oxygen species, TGFβ signaling and apoptosis. Conversely, gene-set of MYC targets showed negative enrichment. These findings suggest that MI treatment exerts cell autonomous and microenvironment cell effects in AML associated with its biologic activity. We also determined important protein-expression correlates of MI activity agnostically, as determined via RPPA analysis, which was further confirmed through a directed CyTOF analysis of MI-treated versus untreated AML cells. Protein expressions that negatively correlated include those involved in protein translation, cell proliferation and survival, whereas protein expressions that positively correlated included inhibitors of growth as well as inducers of apoptosis and loss of cell-survival. Collectively, these perturbations in gene-expressions are consistent with the overall growth inhibitory and lethal effects of MI.

Findings from early phase clinical trials involving MI treatment have demonstrated that primary resistance to MI, as well as emergence of therapy-resistant AML is a common observation [21, 22]. This underscores the need to identify and pre-clinically develop MI-based combinations which would prevent the escape and survival of sub-clonal AML that is resistant to MI therapy [49, 50]. Findings presented here demonstrate for the first time that Menin depletion by CRISPR-Cas9 or Menin degradation by the dTAG system not only reduces MLL1/MLL-FP targets and induces differentiation and lethality, but also sensitizes AML cells with MLL1-r or mtNPM1 to BETi- or LSD1i-induced loss of viability. This was further corroborated by findings demonstrating that co-treatment with MI and pan-BETi (OTX015) or BD2-selective BETi (ABBV-744), or LSD1i, synergistically induced in vitro loss of viability not only of AML cell lines but also PD AML cells that harbor either MLL1-r or mtNPM1 along with FLT3-ITD and/or mtFLT3 (TKD). Combined therapy with MI and pan-BETi was also superior to monotherapy with these drugs in a PDX model of MLL1-r AML with FLT3-TKD. Compared to treatment with MI alone co-treatment with MI and BETi was clearly more potent in inhibiting active chromatin and reducing binding by important oncoprotein transcription factors. Combination treatment with MI and BETi was superior to monotherapy with MI in negatively enriching gene sets of not only HOXA9-MEIS1 and MYC targets but also of mTORC1 signaling, mRNA processing, oxidative phosphorylation, ribosome biogenesis, protein translational initiation and elongation. Moreover, co-treatment with MI and BETi versus MI alone positively enriched gene-sets for TGFβ signaling, stem cell differentiation, TP53 pathway, TNFα signaling via NFkB, interferon α and inflammatory responses and apoptosis. RPPA data presented here also revealed that combination of MI and BETi treatment repressed proteins involved in protein translation and cell-cycle progression, but induced protein expressions involved in DNA damage response, cell cycle inhibition and cell death. Whether selected MI-induced mRNA and protein expression perturbations would serve as correlates of clinical activity of MI will require their prospective evaluation in clinical trial settings. The CRISPR screen conducted in MOLM13, and MV4–11 cells also highlighted dependencies on “druggable” targets, including BRD4, KDM1A, EP300 and MOZ, as well as confirmed previously described dependency on DOT1L [51]. Consistent with these findings, co-treatment with MI and CBP/p300 (GNE-049 and GNE-781) or MOZ (WM1119) inhibitor synergistically induced loss of viability in AML cells with MLL1-r or mtNPM1. The in vitro synergistic lethality induced by co-treatment with MI and BETi or CBP/p300i was also associated with superior in vivo efficacy, highlighted by greater reduction of AML burden and significantly improved survival of the mice due to the combination versus each drug alone in the xenograft model of MOLM13 cells. Preclinical efficacy of the synergistic combination of MI and BETi or GNE-781 validates BRD4 and CBP/p300 as co-dependencies in MI-treated AML cells. BRD4 and CBP/p300 are transcriptional co-factors for the enhancer-driven expression of MYC, MYB, RUNX1, CDK6 and BCL2. Hence, the combination of MI with BETi or HATi would be particularly effective against AML cells that resist MI-induced lethality due to active epigenetic mechanisms and addiction to oncogene expressions driven by BRD4. Whether this synergistic combination would abrogate adaptive mechanisms that lead to emergence of MI-tolerant/persister cells following MI monotherapy remains to be fully evaluated [52, 53].

Although uncommon and observed in approximately 10% of patients with MLL1-r AML, missense mutations and/or allelic loss of TP53 is sub-clonal [18, 37, 38]. It rarely occurs in AML with mtNPM1 [37]. The presence of these TP53 lesions is associated with resistance to lethal activity of DNA-damaging AML chemotherapy and with poor clinical outcomes [37, 40]. Although treatment with hypomethylating agents with or without venetoclax are currently under investigation, none of these agents have so far overcome therapy resistance conferred by TP53 lesions and improved clinical outcomes [54, 55]. Utilizing previously described paired isogenic MOLM13 cells into which TP53 mutations R175H and R248Q had been introduced [40], our findings here demonstrate that co-treatment with MI and BETi or CBP/p300i exerted synergistic lethality in MOLM13-TP53-R175H or -R248Q cells. We had previously reported that co-treatment with MI and venetoclax is synergistically lethal against MOLM13-R175H or -R248Q cells [18]. Furthermore, a previous report had also shown that combination of MI with the FLT3 kinase inhibitor gilteritinib exerted synergistic lethality against MOLM13-TP53 + / + as well as MOLM13-TP53-R175H or -R248Q cells or MOLM13-TP53-/- cells [18, 56, 57]. Taken together, these observations identify several promising MI-based combinations that could be developed and tested in vivo models of MLL1-r AML with or without TP53 lesions.

In AML with mtNPM1, the observed relative sensitivity to cytarabine or other DNA-damaging chemotherapy is well documented [20, 58]. Previous reports have also highlighted sensitivity of this AML sub-type to venetoclax [59]. Despite documentation of Menin-MLL1-dependent, HOXA9-Meis1-target gene expressions in AML with mtNPM1 [14, 15, 20], MI-induced lethality is variable in this AML sub-type [21, 22]. This variability of response and resistance to differentiation induction due to MI treatment could partly be due to the presence of co-mutations in other genes, including FLT3, RAS pathway genes, RUNX1 and epigenetic regulators [60]. These co-mutations may promote MI-tolerant/persister state and escape from MI therapy through a specific dysregulated transcriptome and proteome in these cells. Thus, the disparate response to MI treatment may be due to MI-induced perturbations in gene-expressions that confer MI-tolerant/persister state promoting survival of AML stem/progenitor cells [42, 61]. Overall, findings presented, and combined with previous reports, clearly underscore the need to test rational MI-based combinations that would overcome escape mechanisms in AML controlled by epigenetic drivers such as BRD4 or HATs, e.g., by employing MI plus BETi or HATi; or by growth promoting signaling kinases such as CDK4/6 or FLT3, e.g., by MI plus abemaciclib or MI plus quizartinib; or by anti-apoptotic proteins such as BCL2, e.g., by combining MI with venetoclax [18, 57]. Since the submission and during review of this manuscript, a new report has highlighted that missense mutations in Menin that hinder the binding of MI to Menin, cause emergence and growth of AML resistant to MI treatment leading to relapse [62]. This resistance mechanism is likely to depend on the site of binding of MI to Menin, binding avidity, and/or potency, thereby suggesting that treatment with a rational MI-based synergistic combination might be effective in eliminating the otherwise MI-resistant AML clone and prevent relapse with the therapy-refractory AML. To confirm this, in vitro and in vivo models of PD MI-tolerant/resistant cells will have to be developed in which the impact of novel MI-based combinations, highlighted here and in previous reports, can be evaluated.

## Supplementary information

Change in Authorship Agreement

Supplemental Figure Legends

Supplemental Figures

Supplemental Materials and Methods

Reproducibility Checklist

## Data Availability

Bulk ATAC Seq, single-cell ATAC-Seq, ChIP-Seq, bulk RNA-Seq, and single-cell RNA-Seq datasets have been deposited in GEO and assigned Accession IDs GSE190719 and GSE228326 as part of a Super Series.
