# Correction: Targeting of epigenetic co-dependencies enhances anti-AML efficacy of Menin inhibitor in AML with MLL1-r or mutant NPM1

**DOI:** 10.1038/s41408-025-01306-9

**Published:** 2025-05-21

**Authors:** Warren Fiskus, Christopher P. Mill, Christine Birdwell, John A. Davis, Kaberi Das, Steffen Boettcher, Tapan M. Kadia, Courtney D. DiNardo, Koichi Takahashi, Sanam Loghavi, Michael J. Soth, Tim Heffernan, Gerard M. McGeehan, Xinjia Ruan, Xiaoping Su, Christopher R. Vakoc, Naval Daver, Kapil N. Bhalla

**Affiliations:** 1 The University of Texas MD Anderson Cancer Center, Houston, TX USA; 2 University of Zurich and University Hospital Zurich, CH-8091 Zurich, Switzerland; 3 Syndax Pharmaceuticals, Waltham, MA USA; 4 Cold Spring Harbor Laboratory, Cold Spring Harbor, NY 11724 USA

Correction to: *Blood Cancer Journal* 10.1038/s41408-023-00826-6, published online 13 April 2023

Following the publication of this article an error was noted in Fig. 1. In the original publication, in panel A of Fig. 1, an incorrect Western blot for PBX3 was inadvertently used during the preparation of the figure.

The correct Western blot for PBX3 in panel A of Fig. 1 is presented below. The correction of this figure does not affect the reported results or the conclusions of the article.
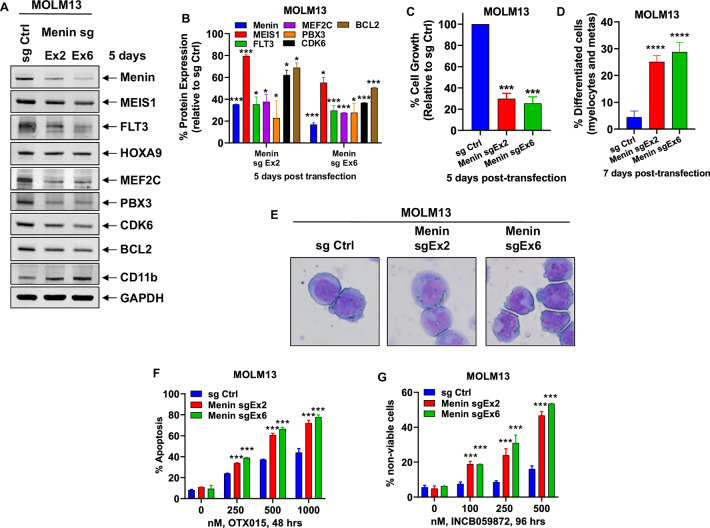


The original article has been corrected.

