# Supplementary material for: Targeting of epigenetic co-dependencies enhances anti-AML efficacy of Menin inhibitor in AML with MLL1-r or mutant NPM1
Source: Blood Cancer J. 2023 Apr 13;13(1):53. doi: 10.1038/s41408-023-00826-6 (PMC10102188; doi:10.1038/s41408-023-00826-6)
Supplement: Supplementary file 1 — Change in Authorship Agreement [file 41408_2023_826_MOESM1_ESM.pdf]

Warren Fiskus[1],

Fri 3/31/2023 3:49 PM

Fiskus,Warren C

RE: Change in authorship agreement for 23-BCJ-0116RR

To Bhalla,Kapil

I agree to the change in authorship for the manuscript

Regards,

Warren Fiskus

Christopher P. Mill[1],

Fri 3/31/2023 3:41 PM

Mill,Christopher P

RE: Change in authorship agreement for 23-BCJ-0116RR

To Fiskus,Warren C

Greetings Warren,

I agree to the change in authorship in the revised menin manuscript.

Regards,

Christopher

Christine Birdwell[1],

Fri 3/31/2023 4:24 PM

Birdwell,Christine E

RE: Change in authorship agreement for 23-BCJ-0116RR

To Fiskus,Warren C

Dear Warren,

I agree to the authorship changes.

Sincerely,

Christine Birdwell

John A. Davis[1],

Fri 3/31/2023 3:42 PM

Davis,John A

RE: Change in authorship agreement for 23-BCJ-0116RR

To Fiskus,Warren C; Bhalla,Kapil; Steffen.Boettcher@usz.ch; gmcgeehan@syndax.com; vakoc@cshl.edu; Soth,Michael J; Heffernan,Tim; Su,Xiaoping; Ruan,Xinjia

Cc Mill,Christopher P; Birdwell,Christine E; Das,Kaberi; Kadia,Tapan Mahendra; DiNardo,Courtney; Takahashi,Koichi; Loghavi,Sanam; Daver,Naval

I agree with the aforementioned changes to the manuscript authorship.

John Davis

Kaberi Das[1],

Fri 3/31/2023 3:58 PM

Das,Kaberi

RE: Change in authorship agreement for 23-BCJ-0116RR

To Davis,John A; Fiskus,Warren C; Bhalla,Kapil; Steffen.Boettcher@usz.ch; gmcgeehan@syndax.com; vakoc@cshl.edu; Soth,Michael J; Heffernan,Tim; Su,Xiaoping; Ruan,Xinjia

Cc Mill,Christopher P; Birdwell,Christine E; Kadia,Tapan Mahendra; DiNardo,Courtney; Takahashi,Koichi; Loghavi,Sanam; Daver,Naval

I agree to the change in authorship in the revised manuscript.

Kaberi Das

Steffen Boettcher[2],

Sat 4/1/2023 7:24 AM

Böttcher Steffen <Steffen.Boettcher@usz.ch>

[EXTERNAL] AW: [EXTERN] Change in authorship agreement for 23-BCJ-0116RR

To Fiskus,Warren C; Bhalla,Kapil; gmcgeehan@syndax.com; vakoc@cshl.edu; Soth,Michael J; Heffernan,Tim; Su,Xiaoping; Ruan,Xinjia

Cc Mill,Christopher P; Birdwell,Christine E; Davis,John A; Das,Kaberi; Kadia,Tapan Mahendra; DiNardo,Courtney; Takahashi,Koichi; Loghavi,Sanam; Daver,Naval

THIS EMAIL IS A PHISHING RISK

Do you trust the sender?
The email address is: steffen.boettcher@usz.ch
While this email has passed our filters, we need you to review with caution before taking any action.
If the email looks at all suspicious, click the Report a Phish button.

I agree as well.

Best,

Steffen

Tapan M. Kadia[1],

Sat 4/1/2023 2:15 PM

Kadia,Tapan Mahendra

Re: Change in authorship agreement for 23-BCJ-0116RR

To Fiskus,Warren C

Cc Heffernan,Tim; Bhalla,Kapil

Thanks Warren. Yes of course I approve!

Sent from my iPhone

Courtney D. DiNardo[1],

Fri 3/31/2023 4:05 PM

DiNardo,Courtney

Re: Change in authorship agreement for 23-BCJ-0116RR

To Das,Kaberi

Cc Davis,John A; Fiskus,Warren C; Bhalla,Kapil; Steffen.Boettcher@usz.ch; gmcgeehan@syndax.com; vakoc@cshl.edu; Soth,Michael J; Heffernan,Tim; Su,Xiaoping; Ruan,Xinjia; Mill,Christopher P; Birdwell,Christine E; Kadia,Tapan Mahendra; Takahashi,Koichi; Loghavi,Sanam; Daver,Naval

I agree to the change in authorship as proposed

Sent from my iPhone

Koichi Takahashi[1],

Fri 3/31/2023 3:16 PM

Takahashi,Koichi

RE: Change in authorship agreement for 23-BCJ-0116RR

To Fiskus,Warren C

Approve!

Sanam Loghavi[1],

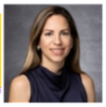

Fri 3/31/2023 4:27 PM

Loghavi,Sanam

Re: [EXTERNAL] RE: Change in authorship agreement for 23-BCJ-0116RR

To Gerard McGeehan

Cc Das,Kaberi; Davis,John A; Fiskus,Warren C; Bhalla,Kapil; steffen.boettcher@usz.ch; vakoc@cshl.edu; Soth,Michael J; Heffernan,Tim; Su,Xiaoping; Ruan,Xinjia; Mill,Christopher P; Birdwell,Christine E; Kadia,Tapan Mahendra; DiNardo,Courtney; Takahashi,Koichi; Daver,Naval

I agree

Michael J. Soth[1],

Fri 3/31/2023 4:10 PM

Soth,Michael J

RE: Change in authorship agreement for 23-BCJ-0116RR

To Fiskus,Warren C

I agree to the change.

Michael J. Soth, Ph.D.
Institute Director, Medicinal Chemistry
Institute for Applied Cancer Science
MD Anderson Cancer Center
E-mail: MJSoth@mdanderson.org
Phone: 713-745-3914

Tim Heffernan[1],

Sat 4/1/2023 2:32 PM

Heffernan,Tim

Re: Change in authorship agreement for 23-BCJ-0116RR

To Kadia,Tapan Mahendra

Cc Fiskus,Warren C; Bhalla,Kapil

I approve. Thank you Warren

Sent from my iPhone

Gerard M. McGeehan[3],

Fri 3/31/2023 4:07 PM

Gerard McGeehan <gmcgeehan@syndax.com>

[EXTERNAL] RE: Change in authorship agreement for 23-BCJ-0116RR

To Das,Kaberi; Davis,John A; Fiskus,Warren C; Bhalla,Kapil; Steffen.Boettcher@usz.ch; vakoc@cshl.edu; Soth,Michael J; Heffernan,Tim; Su,Xiaoping; Ruan,Xinjia

Cc Mill,Christopher P; Birdwell,Christine E; Kadia,Tapan Mahendra; DiNardo,Courtney; Takahashi,Koichi; Loghavi,Sanam; Daver,Naval

THIS EMAIL IS A PHISHING RISK

Do you trust the sender?
The email address is: gmcgeehan@syndax.com
While this email has passed our filters, we need you to review with caution before taking any action.
If the email looks at all suspicious, click the Report a Phish button.

I agree to the change as well.

Jerry McGeehan, Ph.D.
Vice President, Menin Program
Syndax Pharmaceuticals, Inc.
C. 484.431.9385
gmcgeehan@syndax.com

Xinjia Ruan[1],

Fri 3/31/2023 3:45 PM

Ruan,Xinjia

RE: Change in authorship agreement for 23-BCJ-0116RR

To Fiskus,Warren C

Dear Warren,

Thanks for your email. I agree to the change of authorship in the revised manuscript.

Best regards,
Xinjia Ruan

Xiaoping Su[1],

Fri 3/31/2023 3:35 PM

Su,Xiaoping

RE: Change in authorship agreement for 23-BCJ-0116RR

To Fiskus,Warren C

I agree to the change in authorship in the revised manuscript.

Thanks!
Xiaoping

Christopher R. Vakoc[4],

Fri 3/31/2023 3:54 PM

Vakoc, Christopher <vakoc@cshl.edu>

[EXTERNAL] Re: Change in authorship agreement for 23-BCJ-0116RR

To Fiskus,Warren C

THIS EMAIL IS A PHISHING RISK
Do you trust the sender?
The email address is: vakoc@cshl.edu
While this email has passed our filters, we need you to review with caution before taking any action.
If the email looks at all suspicious, click the Report a Phish button.

I agree to this authorship change
Sent from my iPhone

Naval Daver[1],

Fri 3/31/2023 3:14 PM

Daver,Naval

Re: Change in authorship agreement for 23-BCJ-0116RR

To Fiskus,Warren C; Bhalla,Kapil

I agree with this change in authorship.

Thanks

Naval Daver

Kapil N. Bhalla[1]

I agree with the change in authorship in the revised manuscript.

Kapil
