# Supplementary material for: Targeting of epigenetic co-dependencies enhances anti-AML efficacy of Menin inhibitor in AML with MLL1-r or mutant NPM1
Source: Blood Cancer J. 2023 Apr 13;13(1):53. doi: 10.1038/s41408-023-00826-6 (PMC10102188; doi:10.1038/s41408-023-00826-6)
Supplement: Supplementary file 2 — Supplemental Figure Legends [file 41408_2023_826_MOESM2_ESM.docx]

**Supplemental Figure Legends**

**Figure S1. Menin expression is a dependency in AML cells and knockout of Menin depletes MLL target gene expressions.** **A**. Log2 expression of Menin and effect of MEN1 (Menin) knockout in AML cells determined by DEPMAP utilizing the Avana sgRNA library. Dependency was scored based on cell proliferation effects of introducing the sgRNA. A lower score means a greater dependency on MEN1. **B**. MOLM13 cells were transfected with sgNeg or sg Menin Ex 2 or Ex 6 and incubated for 5 days. Then, total RNA was isolated and reverse transcribed. The resulting cDNA was utilized for qPCR analysis. The expression of each mRNA was normalized to GAPDH and compared relative to the sg Negative Ctrl-transfected cells.

**Figure S2. dTAG13-mediated degradation of Menin increases sensitivity to treatment with BET inhibitor or LSD1 inhibitor in MLL-r AML cells. A**. MOLM13-Menin-FKBP12^F36V^ and MV4-11-Menin-FKBP12^F36V^ cells were treated with the indicated concentrations of dTAG-13 for 24 hours. At the end of treatment, cell lysates were prepared and immunoblot analyses were conducted for HA-tagged Menin to document degradation of Menin. The expression levels of GAPDH served as the loading control. **B-C**. MOLM13-Menin-FKBP12^F36V^ and MV4-11-Menin-FKBP12^F36V^ cells were treated with the indicated concentrations of OTX015 for 48 hours alone or co-treated with 250 nM of dTAG-13. The % of TO-PRO-3 iodide-positive, non-viable cells were determined by flow cytometry. Mean of two independent experiments performed in duplicate + S.D. *= p<0.05; **=p<0.01; ***=p<0.005 compared to MOLM13-Menin-FKBP12^F36V^ cells not treated with dTAG-13 (determined by a two-tailed, unpaired t-test in GraphPad V8). **D-E.** MOLM13-Menin-FKBP12^F36V^ MV4-11-Menin-FKBP12^F36V^ cells were treated with the indicated concentrations of INCB0598672 for 96 hours without or with 500 nM of dTAG-13. The % of TO-PRO-3 iodide-positive cells were determined by flow cytometry. Mean of two independent experiments performed in duplicate + S.D. *= p<0.05; **=p<0.01; ***=p<0.005 compared to MOLM13-Menin-FKBP12^F36V^ cells not treated with dTAG-13 (determined by a two-tailed, unpaired t-test in GraphPad V8).

**Figure S3. Treatment with Menin inhibitor depletes chromatin accessibility in MLL fusion target genes in MLL-r AML as well as genes involved in RNA Pol II transcription in mtNPM1-expressing OCI-AML3 cells. A-C.** IGV plots of ATAC-Seq peak density at the MEIS1, SENP6 and CDK6 locus in SNDX-50469-treated MOLM13 and the MYB and TCF4 locus in SNDX-50469-treated OCI-AML3 cells. **D**. Log2 fold-change in chromatin accessibility (ATAC peaks) in RNA Pol II transcription genes in OCI-AML3 cells. **E**. Menin inhibitor-induced log2 fold-change in H3K27Ac ChIP-Seq peaks (enhancers and promoters) in selected MLL-fusion target genes as determined by DiffReps.

**Figure S4. Treatment with Menin inhibitor depletes mRNA expression in MLL-r and concordantly depletes chromatin accessibility and mRNA expression in mtNPM1 expressing AML cells. A.** MOLM13 cells were treated with 500 nM of SNDX-50469 for 16 hours as biologic replicates. Total RNA was isolated and utilized for RNA-Seq analysis. Heatmap shows the number of induced and depleted RNAs due to SNDX-50469 treatment at p < 0.05**. B.** RNA-Seq-determined log2 fold-changes in mRNA expression due to treatment with the indicated concentration of SNDX-50469 for 16 hours in MOLM13 cells compared to the ROSS_LEUKEMIA_WITH_MLL_FUSIONS target gene dataset. **C**. Volcano plot of RNA-Seq-determined mRNA expression changes (>1.25 fold-change and p<0.05) in MV4-11 cells treated with the indicated concentration of SNDX-50469 for 16 hours. **D**. Gene set enrichment analysis of SNDX-50469 treated MV4-11 cells compared to HALLMARK and REACTOME pathways. All q-values were less than 0.1. **E.** OCI-AML3 cells were treated with 500 nM of SNDX-50469 for 16 hours as biologic replicates. Total RNA was isolated and utilized for RNA-Seq analysis. Volcano plot shows the RNA-Seq-determined mRNA expression changes (>1.25 fold-change and p<0.05) **F**. Gene set enrichment analysis of SNDX-50469 treated OCI-AML3 cells compared to selected HALLMARK and KEGG pathways. All q-values were less than 0.1. **G-H**. Circos plot and log2 fold-changes of selected concordant ATAC-Seq and mRNA expression alterations in SNDX-50469-treated OCI-AML3 cells.

**Figure S5**. **Treatment with Menin inhibitor induces overlapping mRNA expression alterations in MLL1-r and mtNPM1 expressing AML cells. A-B**. Venn diagram of overlapping upregulated and downregulated gene expression alterations in MOLM13 and OCI-AML3 cells treated with 500 nM of SNDX-50469 for 16 hours. **C**. Gene name and log2 fold-change of the 200 overlapping induced genes in MOLM13 and OCI-AML3 cells treated with SNDX-50469 for 16 hours. **D**. Gene name and log2 fold-change of the 99 overlapping depleted genes in MOLM13 and OCI-AML3 cells treated with SNDX-50469 for 16 hours.

**Figure S6**. **Menin inhibitor depletes MLL fusion target genes, reduces the number of cells with an HSC/LSC mRNA signature in patient-derived MLL1-r AML cells and induces protein expression changes in MLL1-r AML cells. A.** PD, MLL-AF9 + FLT3-TKD cells treated with 500 nM of SNDX-50469 for 16 hours were analyzed by single-cell RNA Seq analysis. Heat map shows SNDX-50469-mediated log2 fold-change in the marker genes defining the 11-clusters. **B**. UMAP plot of MLL1 fusion target genes MYB, LAMP5, SENP6, FLT3 and CDK6 at the single-cell level in SNDX-50469-treated (500 nM, 16 hrs) MLL-AF9 + FLT3-TKD AML cells. **C**. UMAP plot of cell-type designation determined by mRNA expression utilizing the Single-R algorithm. SNDX-50469 treatment reduces the # of PD, MLL-AF9 + FLT3-TKD cells exhibiting an HSC/LSC mRNA signature. **D-E.** PD, MLL-AF9 + FLT3-TKD AML cells were treated with the indicated concentrations of SNDX-50469 for 48 hours in biologic triplicates. Cells were harvested and reverse phase protein array (RPPA) analysis was conducted. The heat map (**D**) shows the total number of significantly (p < 0.05) up and down-regulated proteins in each cell sample due to SNDX-50469 treatment. The volcano plot (**E**) highlights the most significantly (p < 0.05) altered proteins in each cell sample due to SNDX-50469 treatment.

**Figure S7. Epigenetic and chromatin modifying domain-focused CRISPR screen identified dependencies in MLL1-r AML MOLM13 and MV4-11 cells.** **A-B.** MOLM13-Cas9 and MV4-11 Cas9 expressing cells were transduced (biologic replicates) with a library of domain-specific sgRNAs against chromatin modifying proteins and incubated for 12 days. Live cells were harvested; genomic DNA was isolated and minimally amplified with primers flanking the sgRNA sequences. Sequencing libraries were generated and amplicon-seq was performed. The graph shows log2 fold-changes in sgRNAs which dropped out significantly (p < 0.05 and f.d.r. <0.05) at day 12 post-transduction versus day 2 post-transduction in both replicates for both cell lines. **C-E.** MOLM13, MV4-11 and OCI-AML3 cells were treated with the indicated concentrations of SNDX-50469 and/or OTX015 for 96 hours. At the end of treatment, the % non-viable cells were determined by staining with TO-PRO-3 iodide and flow cytometry analysis. **F-G**. THP1, and OCI-AML2 cells were treated with the indicated concentrations of SNDX-50469 and/or OTX015 for 96 hours. At the end of treatment, the % non-viable cells were determined by staining with TO-PRO-3 iodide and flow cytometry analysis. Delta synergy scores were determined by the ZIP method within the SynergyFinder 2.0 web application. Synergy scores >1.0 indicate a synergistic interaction of the two agents in the combination.

**Figure S8. Co-treatment with Menin inhibitor and OTX015 induces synergistic *in vitro* lethality in patient-derived AML cells with MLL1-r or mtNPM1 expression with or without mt FLT3**. **A**. Oncoplot of the mutations identified (by Next-Gen sequencing of an 81-gene panel) in the patient-derived, de novo AML samples utilized in these studies. **B-I**. PD, MLL1-r AML cells or mtNPM1 expressing AML cells with or without mtFLT3 or FLT3-ITD were treated with the indicated concentrations of SNDX-50469 and/or OTX015 for 72 hours. At the end of treatment, the % non-viable cells were determined by staining with TO-PRO-3 iodide and flow cytometry analysis. Delta synergy scores were determined by the ZIP method within the SynergyFinder 2.0 web application. Synergy scores >1.0 indicate a synergistic interaction of the two agents in the combination. **J**. Combination index values for SNDX-50469 and OTX015 calculated utilizing Compusyn (Chou and Talalay method) for the PD MLL1-r and mtNPM1 expressing AML cells in (**B-I**)

**Figure S9.** **Co-treatment with Menin inhibitor and BD2 selective BET inhibitor ABBV-744 induces synergistic *in vitro* lethality in AML cells with mtNPM1 or MLL1-r A-C**. MV4-11, MOLM13 and OCI-AML3, cells were treated with the indicated concentrations of SNDX-50469 and/or ABBV-744 for 96 hours. At the end of treatment, the % non-viable cells were determined by staining with TO-PRO-3 iodide and flow cytometry analysis.

**Figure S10.** **Co-treatment with Menin inhibitor and OTX015 or BD2 selective BET inhibitor ABBV-744 induces synergistic *in vitro* lethality in MLL1-r AML MOLM13 cells with isogenic TP53 mutations. A-D**. MOLM13 TP53-R175H and MOLM13 TP53-R248Q cells were treated with the indicated concentrations of SNDX-50469 and/or OTX015 or ABBV-744 for 96 hours. At the end of treatment, the % non-viable cells were determined by staining with TO-PRO-3 iodide and flow cytometry analysis. Delta synergy scores were determined by the ZIP method within the SynergyFinder 2.0 web application. Synergy scores >1.0 indicate a synergistic interaction of the two agents in the combination.

**Figure S11. Co-treatment with Menin inhibitor and bromodomain-targeting HAT inhibitor GNE-049 exerts synergistic *in vitro* lethality in cultured and patient-derived AML cells with MLL1-r or expressing mtNPM1 with or without FLT3-ITD or mtFLT3**. **A-F**. MV4-11, OCI-AML3, OCI-AML2, MOLM13 TP53-WT, MOLM13 TP53-R175H, and MOLM13 TP53-R248Q cells were treated with the indicated concentrations of SNDX-50469 and/or GNE-049 for 96 hours. At the end of treatment, the % non-viable cells were determined by staining with TO-PRO-3 iodide and flow cytometry analysis. Delta synergy scores were determined by the ZIP method within the SynergyFinder 2.0 web application. Synergy scores >1.0 indicate a synergistic interaction of the two agents in the combination. **G-J**. Patient-derived AML cells with MLL1 rearrangement or mtNPM1 with or without FLT3 alterations were treated with the indicated concentrations of SNDX-50469 and/or GNE-049 for 72 hours. The % non-viable cells were determined by staining with TO-PRO-3 iodide and flow cytometry analysis. Delta synergy scores were determined by the ZIP method within the SynergyFinder 2.0 web application. Synergy scores >1.0 indicate a synergistic interaction of the two agents in the combination. **K**. Combination index values for SNDX-50469 and GNE-049 calculated utilizing CompuSyn (Chou and Talalay method) for the PD MLL1-r and mtNPM1 expressing AML cells. **L-M**. Normal CD34+ HPCs from cord blood were treated with the indicated concentrations of SNDX-50469 and/or OTX015 (n=5) or GNE-049 (n=4) for 72 hours. The % non-viable cells were determined by staining with TO-PRO-3 iodide and flow cytometry analysis.

**Figure S12. Co-treatment with Menin inhibitor and clinic-bound bromodomain-targeting HAT inhibitor GNE-781 or MOZ inhibitor WM1119 exerts synergistic *in vitro* lethality in cultured AML cells with MLL1-r**. **A-C**. MOLM13, MV4-11, and OCI-AML3 cells were treated with the indicated concentrations of SNDX-50469 and/or GNE-781 for 96 hours. At the end of treatment, the % non-viable cells were determined by staining with TO-PRO-3 iodide and flow cytometry analysis. Delta synergy scores were determined by the ZIP method within the SynergyFinder 2.0 web application. Synergy scores >1.0 indicate a synergistic interaction of the two agents in the combination. **D-E**. MV4-11 and MOLM13 cells were treated with the indicated concentrations of SNDX-50469 and/or MOZ inhibitor WM1119 for 96 hours. At the end of treatment, the % non-viable cells were determined by staining with TO-PRO-3 iodide and flow cytometry analysis. Delta synergy scores were determined by the ZIP method within the SynergyFinder 2.0 web application. Synergy scores >1.0 indicate a synergistic interaction of the two agents in the combination.

**Figure S13. Co-treatment with Menin inhibitor and KDM1A inhibitor exerts synergistic *in vitro* lethality in cultured and patient-derived AML cells with MLL1-r or expressing mtNPM1 with or without FLT3-ITD or mtFLT3**. **A-C**. MV4-11, MOLM13 and OCI-AML3 cells were treated with the indicated concentrations of SNDX-50469 and/or INCB059872 for 96 hours. At the end of treatment, the % non-viable cells were determined by staining with TO-PRO-3 iodide and flow cytometry analysis. Delta synergy scores were determined by the ZIP method within the SynergyFinder 2.0 web application. Synergy scores >1.0 indicate a synergistic interaction of the two agents in the combination. **D-F**. Patient-derived AML cells with MLL1 rearrangement or mtNPM1 with or without FLT3 alterations were treated with the indicated concentrations of SNDX-50469 and/or INCB059872 for 72 hours. The % non-viable cells were determined by staining with TO-PRO-3 iodide and flow cytometry analysis. Delta synergy scores were determined by the ZIP method within the SynergyFinder 2.0 web application. Synergy scores >1.0 indicate a synergistic interaction of the two agents in the combination. **G**. Combination index values for SNDX-50469 and INCB059872 calculated utilizing CompuSyn (Chou and Talalay method) for the PD MLL1-r and mtNPM1 expressing AML cells.

**Figure S14** **Compared to SNDX-50469 alone, co-treatment with SNDX-50469 and OTX015 further reduces H3K27Ac occupancy on chromatin and alters transcription of genes in TNF alpha and APOPTOSIS pathways in MOLM13 cells. A-D**. IGV plot of H3K27Ac occupancy on the MEIS1, MEF2C, CDK6 and FLT3 locus in MOLM13 cells treated with 500 nM of SNDX-50469 or SNDX-50469 plus 500 nM of OTX015 for 16 hours. **E**. MOLM13 cells were treated with 500 nM of SNDX-50469 and/or 500 nM of OTX015 for 16 hours. RNA-Seq analysis was performed on biologic replicates. Heat map shows mRNAs depleted or induced greater than 1.25-fold and p< 0.05 by SNDX-50469 + OTX015 over SNDX-50469 treatment alone. **F**. Gene set enrichment analysis of SNDX-50469 + OTX015 treated over SNDX-50469-treated MOLM13 cells compared to HALLMARK, REACTOME and GO pathways. All q-values were less than 0.1.
