# Supplementary material for: Targeting of epigenetic co-dependencies enhances anti-AML efficacy of Menin inhibitor in AML with MLL1-r or mutant NPM1
Source: Blood Cancer J. 2023 Apr 13;13(1):53. doi: 10.1038/s41408-023-00826-6 (PMC10102188; doi:10.1038/s41408-023-00826-6)
Supplement: Supplementary file 3 — Supplemental Figures [file 41408_2023_826_MOESM3_ESM.pdf]

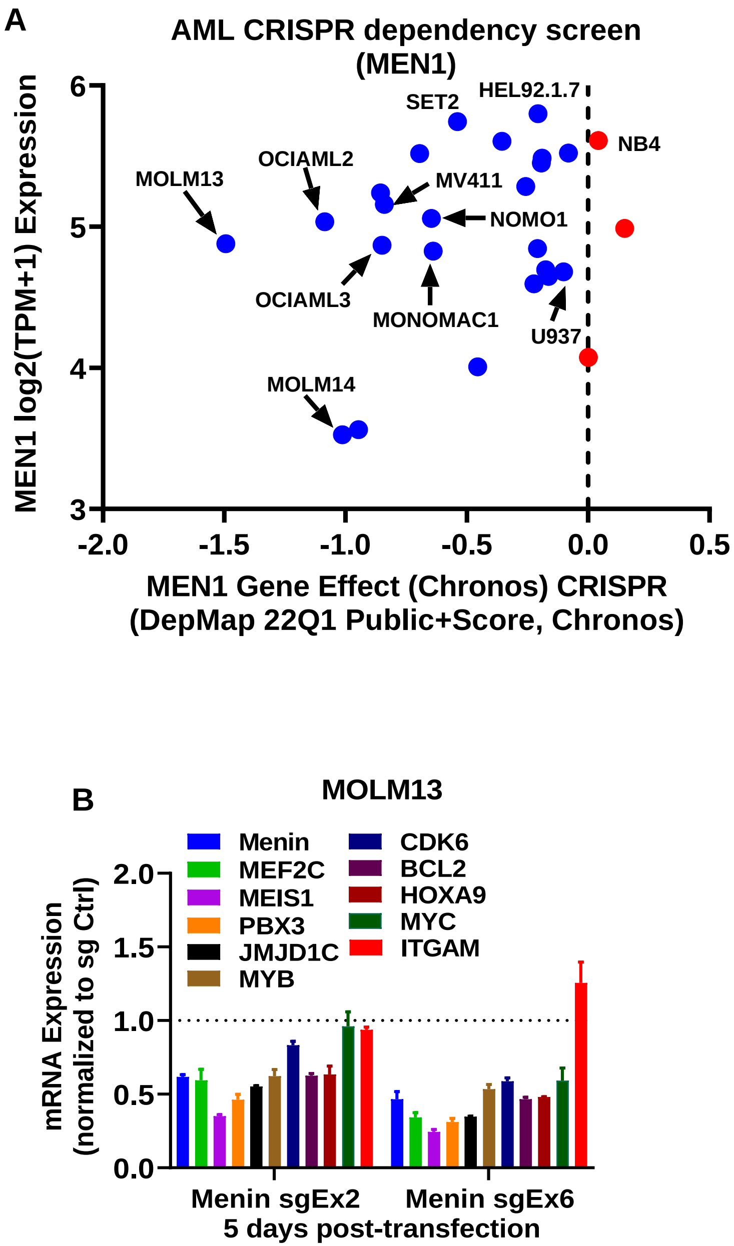
A
AML CRISPR dependency screen (MEN1)
MEN1 log2(TPM+1) Expression
MEN1 Gene Effect (Chronos) CRISPR (DepMap 22Q1 Public+Score, Chronos)
SET2
HEL92.1.7
NB4
OCIAML2
MOLM13
MV411
NOMO1
OCIAML3
MONOMAC1
U937
MOLM14
6
5
4
3
-2.0
-1.5
-1.0
-0.5
0.0
0.5
B
MOLM13
mRNA Expression (normalized to sg Ctrl)
Menin
MEF2C
MEIS1
PBX3
JMJD1C
MYB
CDK6
BCL2
HOXA9
MYC
ITGAM
2.0
1.5
1.0
0.5
0.0
Menin sgEx2
Menin sgEx6
5 days post-transfection

Figure S1

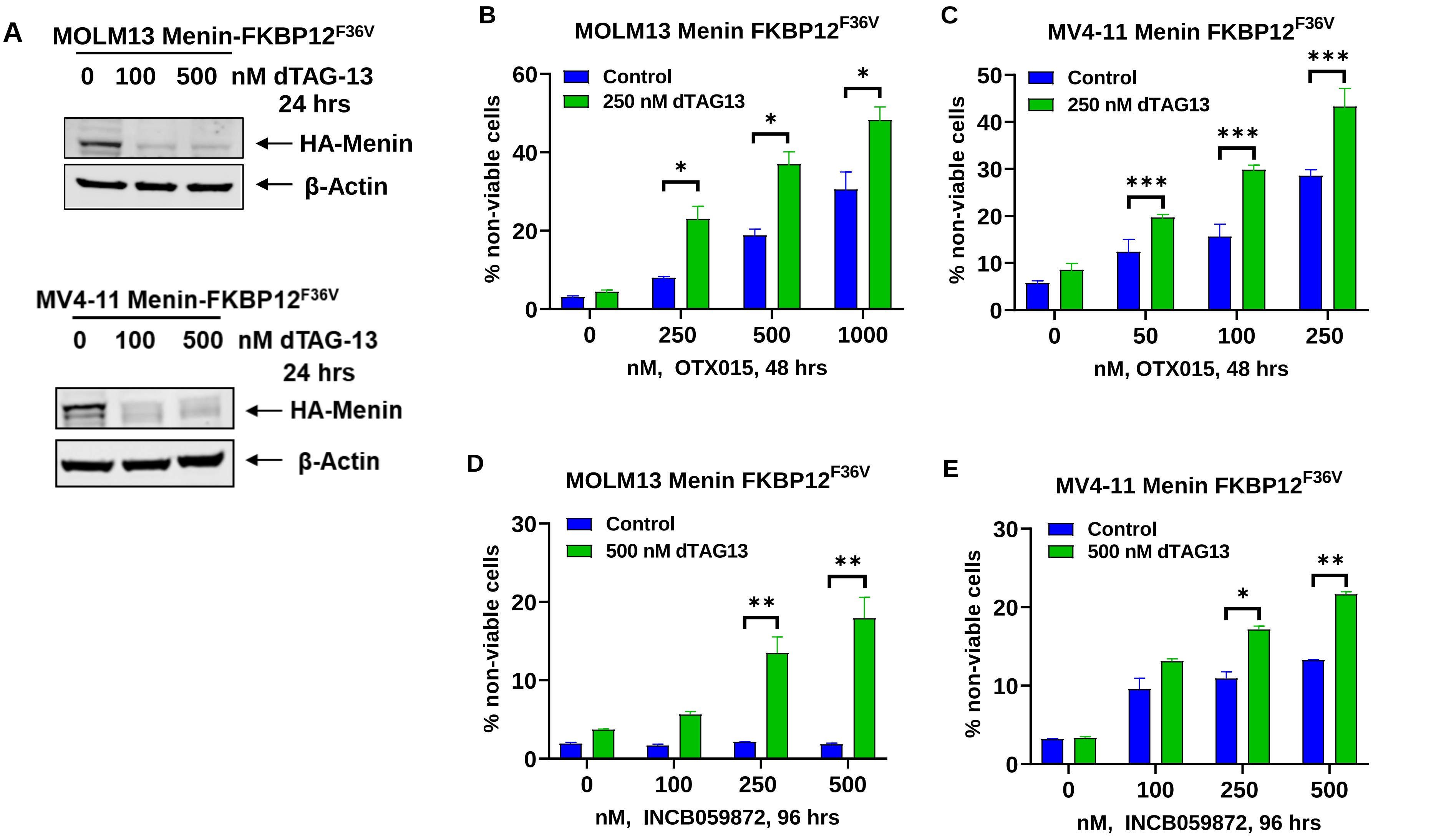
A
MOLM13 Menin-FKBP12F36V
0 100 500 nM dTAG-13
24 hrs
HA-Menin
β-Actin
MV4-11 Menin-FKBP12F36V
0 100 500 nM dTAG-13
24 hrs
HA-Menin
β-Actin
B
MOLM13 Menin FKBP12F36V
Control
250 nM dTAG13
% non-viable cells
0
20
40
60
0
250
500
1000
nM, OTX015, 48 hrs
C
MV4-11 Menin FKBP12F36V
Control
250 nM dTAG13
% non-viable cells
0
10
20
30
40
50
0
50
100
250
nM, OTX015, 48 hrs
D
MOLM13 Menin FKBP12F36V
Control
500 nM dTAG13
% non-viable cells
0
10
20
30
0
100
250
500
nM, INCB059872, 96 hrs
E
MV4-11 Menin FKBP12F36V
Control
500 nM dTAG13
% non-viable cells
0
10
20
30
0
100
250
500
nM, INCB059872, 96 hrs

Figure S2

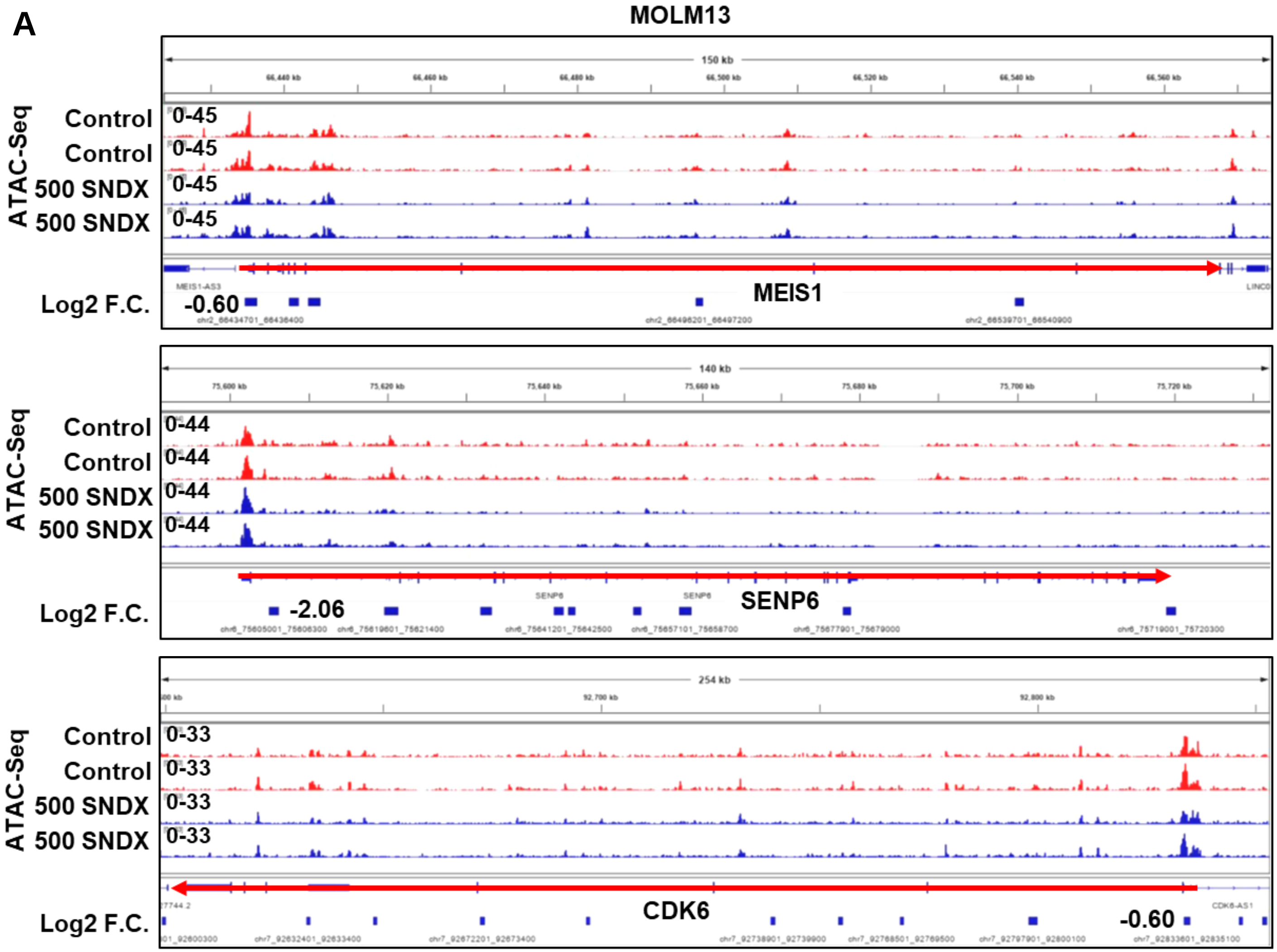
A
MOLM13
ATAC-Seq
Control
Control
500 SNDX
500 SNDX
0-45
Log2 F.C.
-0.60
MEIS1
0-44
-2.06
SENP6
0-33
CDK6
-0.60

Figure S3

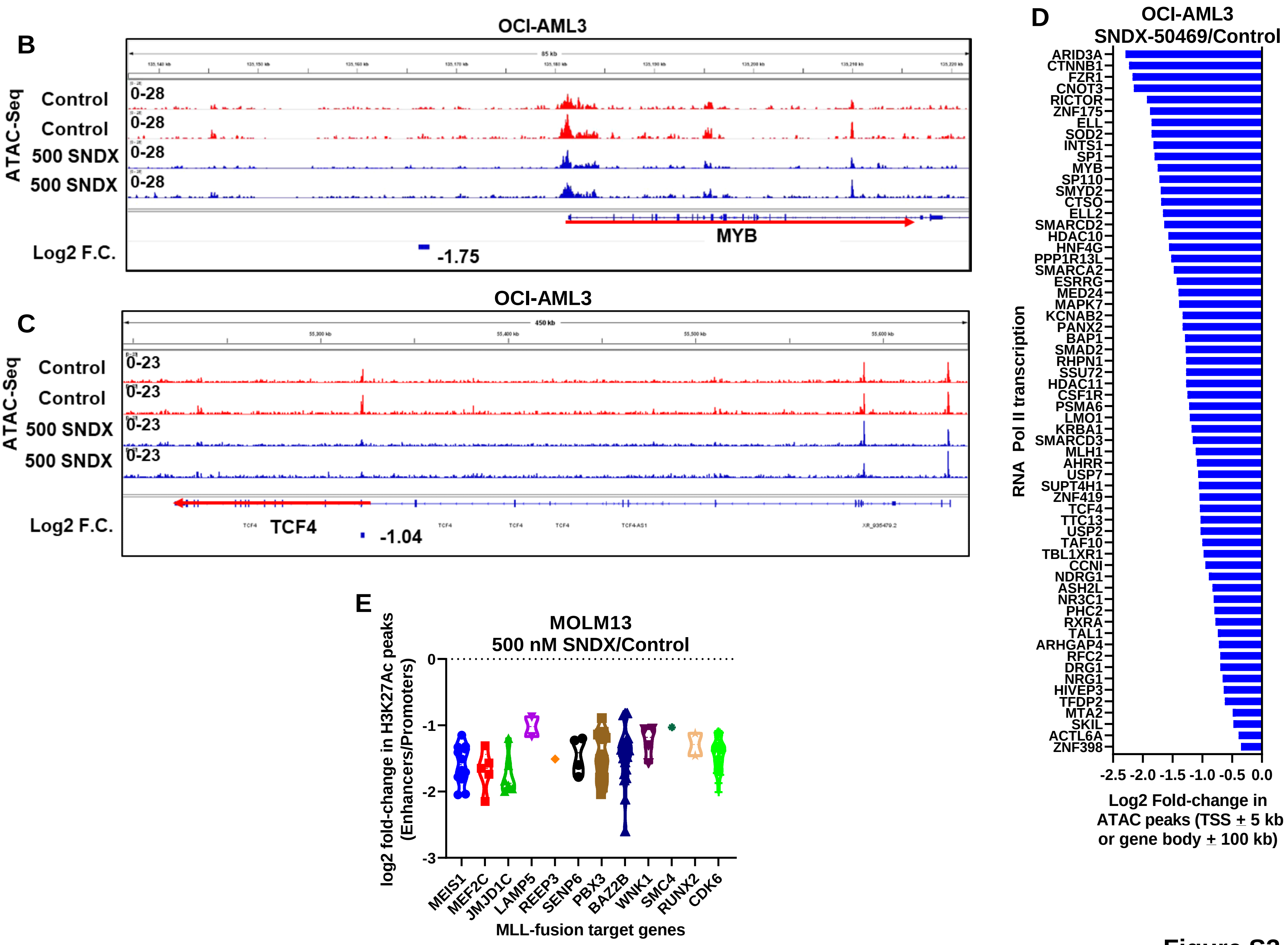

B
OCI-AML3
ATAC-Seq
Control
Control
500 SNDX
500 SNDX
0-28
85 kb
MYB
Log2 F.C.
-1.75
C
OCI-AML3
ATAC-Seq
Control
Control
500 SNDX
500 SNDX
0-23
450 kb
TCF4
Log2 F.C.
-1.04
D
OCI-AML3
SNDX-50469/Control
RNA Pol II transcription
ARID3A
CTNNB1
FZR1
CNOT3
RICTOR
ZNF175
ELL
SOD2
INTS1
SP1
MYB
SP110
SMYD2
CTSO
ELL2
SMARCD2
HDAC10
HNF4G
PPP1R13L
SMARCA2
ESRRG
MED24
MAPK7
KCNAB2
PANX2
BAP1
SMAD2
RHPN1
SSU72
HDAC11
CSF1R
PSMA6
LMO1
KRBA1
SMARCD3
MLH1
AHRR
USP7
SUPT4H1
ZNF419
TCF4
TTC13
USP2
TAF10
TBL1XR1
CCNI
NDRG1
ASH2L
NR3C1
PHC2
RXRA
TAL1
ARHGAP4
RFC2
DRG1
NRG1
HIVEP3
TFDP2
MTA2
SKIL
ACTL6A
ZNF398
-2.5 -2.0 -1.5 -1.0 -0.5 0.0
Log2 Fold-change in ATAC peaks (TSS + 5 kb or gene body + 100 kb)
E
MOLM13
500 nM SNDX/Control
log2 fold-change in H3K27Ac peaks (Enhancers/Promoters)
0
-1
-2
-3
MEIS1
MEF2C
JMJD1C
LAMP5
REEP3
SENP6
PBX3
BAZ2B
WNK1
SMC4
RUNX2
CDK6
MLL-fusion target genes


**Figure S3**

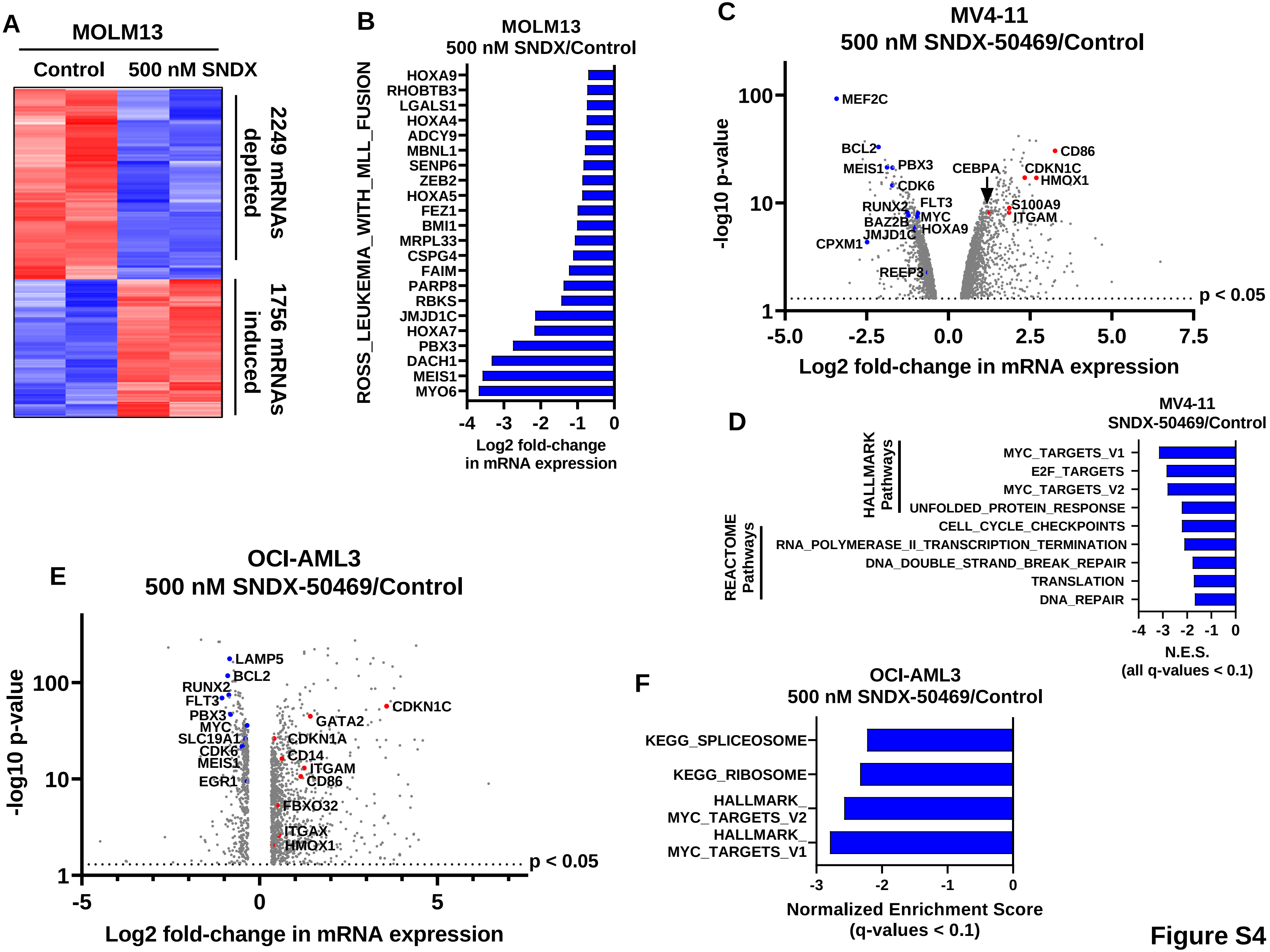
A
MOLM13
Control
500 nM SNDX
2249 mRNAs
depleted
1756 mRNAs
induced
B
MOLM13
500 nM SNDX/Control
ROSS_LEUKEMIA_WITH_MLL_FUSION
HOXA9
RHOBTB3
LGALS1
HOXA4
ADCY9
MBNL1
SENP6
ZEB2
HOXA5
FEZ1
BMI1
MRPL33
CSPG4
FAIM
PARP8
RBKS
JMJD1C
HOXA7
PBX3
DACH1
MEIS1
MYO6
Log2 fold-change
in mRNA expression
C
MV4-11
500 nM SNDX-50469/Control
-log10 p-value
MEF2C
BCL2
MEIS1
PBX3
CDK6
CEBPA
CD86
CDKN1C
HMOX1
RUNX2
FLT3
MYC
BAZ2B
HOXA9
JMJD1C
CPXM1
S100A9
ITGAM
REEP3
p < 0.05
Log2 fold-change in mRNA expression
D
MV4-11
SNDX-50469/Control
HALLMARK Pathways
REACTOME Pathways
MYC_TARGETS_V1
E2F_TARGETS
MYC_TARGETS_V2
UNFOLDED_PROTEIN_RESPONSE
CELL_CYCLE_CHECKPOINTS
RNA_POLYMERASE_II_TRANSCRIPTION_TERMINATION
DNA_DOUBLE_STRAND_BREAK_REPAIR
TRANSLATION
DNA_REPAIR
N.E.S.
(all q-values < 0.1)
E
OCI-AML3
500 nM SNDX-50469/Control
-log10 p-value
LAMP5
BCL2
RUNX2
FLT3
PBX3
MYC
SLC19A1
CDK6
MEIS1
EGR1
CDKN1C
GATA2
CDKN1A
CD14
ITGAM
CD86
FBXO32
ITGAX
HMOX1
p < 0.05
Log2 fold-change in mRNA expression
F
OCI-AML3
500 nM SNDX-50469/Control
KEGG_SPLICEOSOME
KEGG_RIBOSOME
HALLMARK_
MYC_TARGETS_V2
HALLMARK_
MYC_TARGETS_V1
Normalized Enrichment Score
(q-values < 0.1)

Figure S4

G

OCI-AML3 SNDX-50469/Control

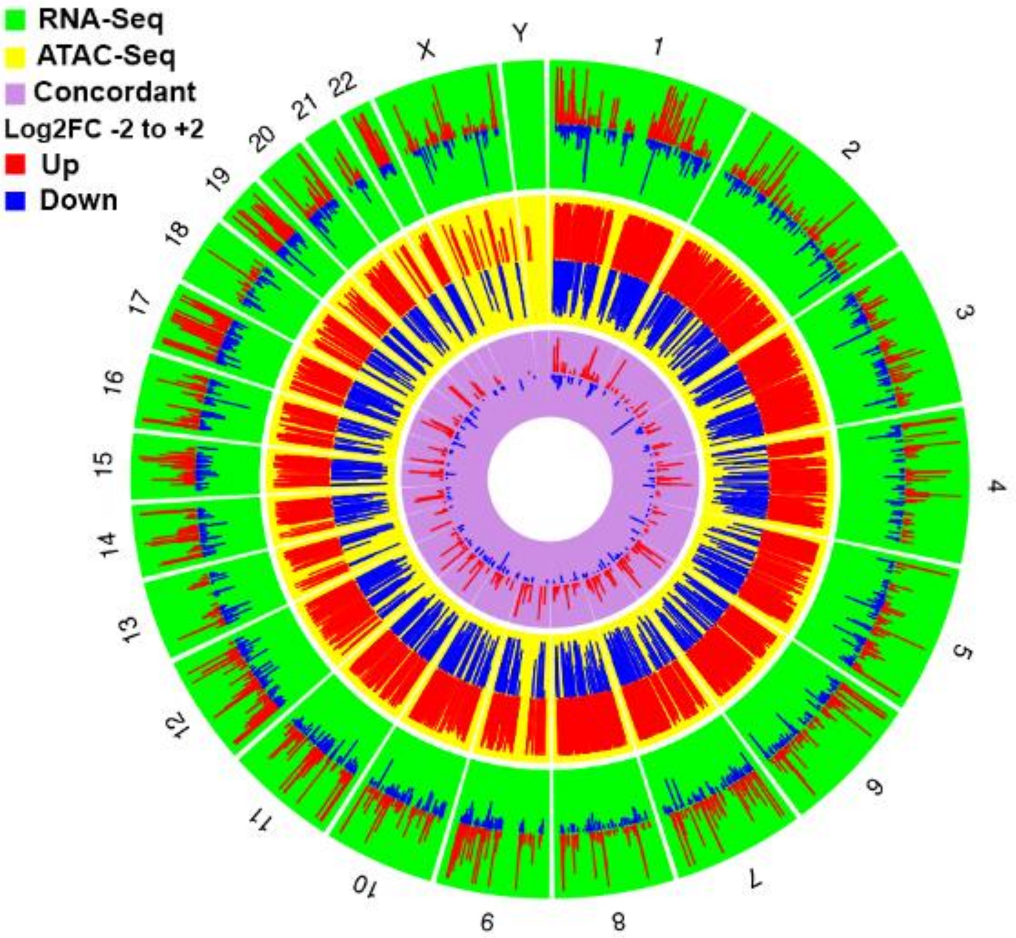


| | |
|---|---|
| # of genes upregulated in RNA-Seq | 2301 |
| # of genes down-regulated in RNA-Seq | 2500 |
| # of differential peaks gained in ATAC-Seq | 3354 |
| # of differential peaks lost in ATAC-Seq | 1134 |
| # of up genes in both RNA-Seq and ATAC-Seq | 267 |
| # of down genes in both RNA-Seq and ATAC-Seq | 192 |
| # of genes common in RNA-Seq and ATAC-Seq | 832 |

H

OCI-AML3
500 nM SNDX-50469/Control

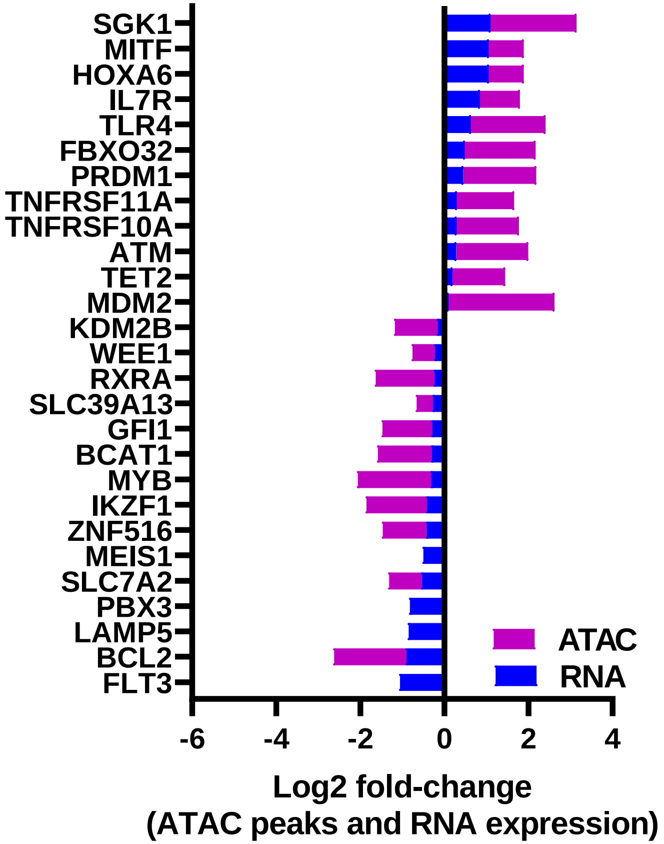


Figure S4

**A**

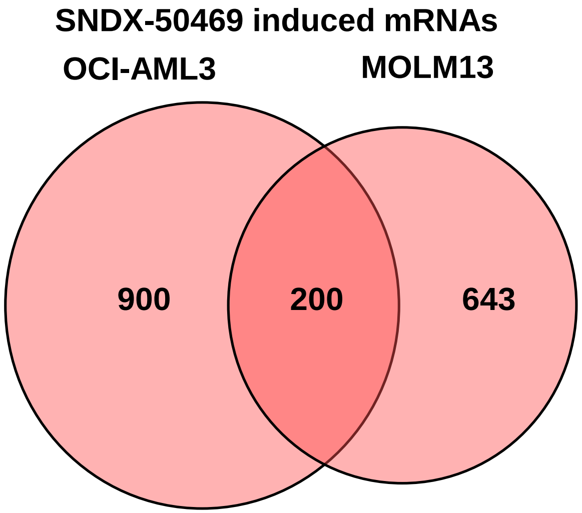
SNDX-50469 induced mRNAs
OCI-AML3
MOLM13
900
200
643

**B**

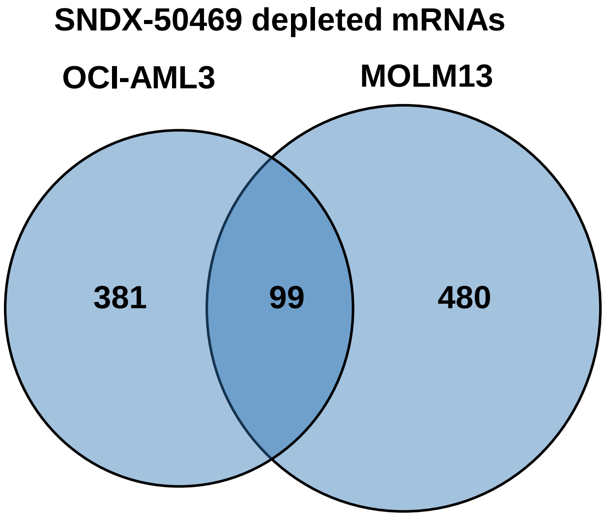
SNDX-50469 depleted mRNAs
OCI-AML3
MOLM13
381
99
480

**Figure S5**

C

| Gene Name | OCI3_log2 Fold-Change | MOLM_log2 Fold-Change |
|---|---|---|
| ANXA1 | 0.769126671 | 2.050580391 |
| METTL7A | 1.130841473 | 0.847306002 |
| TUBA1A | 1.131696753 | 0.344448454 |
| SAMD9L | 1.15998037 | 0.983014002 |
| LRP10 | 0.586423591 | 0.412973253 |
| FUCA1 | 0.951084363 | 0.431478809 |
| TPPP | 1.620478169 | 0.619654256 |
| KIAA0040 | 1.618970702 | 1.50893655 |
| LILRA2 | 0.643202581 | 0.913427186 |
| TLR4 | 0.635016328 | 1.279735163 |
| ABCA7 | 0.576437837 | 0.587335732 |
| IL7R | 0.850877104 | 0.348329468 |
| USP53 | 0.709446237 | 0.544774289 |
| C3 | 0.662770705 | 1.170914352 |
| F11R | 1.018595921 | 0.485488884 |
| MT2A | 0.810602932 | 0.408295396 |
| KLHL24 | 0.637013546 | 0.484316086 |
| SLC43A2 | 0.59555641 | 0.50981815 |
| BMF | 0.800350424 | 0.921079652 |
| RAB7B | 0.370740315 | 0.453151312 |
| HELZ2 | 0.573110271 | 0.367427521 |
| ARID5B | 0.903714225 | 1.159021156 |
| KIAA0513 | 0.937634336 | 1.639889271 |
| BNIP3L | 0.387214989 | 0.764398148 |
| TRAF3IP3 | 0.581943578 | 0.584583799 |
| SLA | 0.381566838 | 0.794559452 |
| APOL6 | 0.494915234 | 0.484327997 |
| IDS | 0.678759958 | 0.874018668 |
| RAB37 | 0.480812763 | 0.718738274 |
| MAP3K12 | 0.841452642 | 1.161111127 |
| CDC42EP4 | 0.511644176 | 0.475287129 |
| MBNL2 | 0.661794997 | 0.777167958 |
| CD37 | 0.571849756 | 0.527531943 |
| PIM1 | 0.960371466 | 0.393803711 |
| PIAS3 | 0.435995338 | 0.470304992 |
| ITPR2 | 1.00949731 | 0.794325161 |
| CPNE3 | 0.38318417 | 0.344449832 |
| NLRP1 | 0.454880994 | 1.838673337 |
| CCNG2 | 0.446809137 | 0.878540435 |
| KIFC2 | 0.614202696 | 0.353068603 |
| NFKB2 | 0.410809367 | 0.463385655 |
| ABTB1 | 0.663619868 | 0.835468289 |
| SYNE1 | 0.836854313 | 2.893787405 |
| KIAA1551 | 0.424826036 | 0.440958201 |
| TSPOAP1 | 0.443018333 | 0.685331799 |
| RUBCN | 0.396341924 | 0.404241031 |
| CFP | 0.703229456 | 1.097445997 |
| PDE4DIP | 0.632014406 | 0.651619635 |
| FAM111A | 0.341067852 | 0.333478141 |
| SLC26A2 | 0.53570467 | 0.61464032 |
| CXCL10 | 1.155943052 | 1.487722805 |
| UBE2L6 | 0.449416641 | 0.669729105 |
| NAGK | 0.52341085 | 0.453570925 |
| FCGR2B | 1.716012032 | 2.410834719 |
| PBXIP1 | 0.353484532 | 0.479179443 |
| NBR1 | 0.335671912 | 0.326441308 |
| CBLB | 0.584379329 | 0.726848329 |
| GCC2 | 0.464075595 | 0.466215135 |
| ACSF2 | 0.761642782 | 0.63179147 |
| ZNF467 | 0.724357328 | 1.149804555 |
| SYNPO | 0.588428558 | 1.773983207 |
| SLC12A6 | 0.408187986 | 0.520058354 |
| CLCN3 | 0.35760623 | 0.329313288 |
| SV2A | 0.376638509 | 0.403405555 |
| ITGAM | 1.250935324 | 1.193462384 |
| GLTP | 0.446658991 | 0.640877051 |
| IFI6 | 0.769288621 | 1.657136986 |
| IFFO2 | 1.187968924 | 0.711287958 |
| MYOF | 0.461753939 | 0.807093904 |

| Gene Name | OCI3_log2 Fold-Change | MOLM_log2 Fold-Change |
|---|---|---|
| BCAS3 | 0.465697456 | 0.825372617 |
| TMEM87B | 0.348431114 | 0.489412108 |
| NTNG2 | 1.445280839 | 0.897482732 |
| UACA | 0.754659674 | 0.590032069 |
| ZSWIM8 | 0.346082636 | 0.412243771 |
| JUN | 0.394618945 | 0.952660108 |
| NRBP2 | 0.408903007 | 0.451528558 |
| APOL2 | 0.420470922 | 0.380280201 |
| WDR19 | 0.604602433 | 0.390017731 |
| CD86 | 1.158144941 | 1.06250525 |
| CX3CR1 | 0.514037521 | 2.017295783 |
| CTTNBP2NL | 0.49399208 | 0.70557269 |
| SLC44A2 | 0.654738008 | 0.701089744 |
| BBS2 | 0.362820962 | 0.607857715 |
| STXBP1 | 0.448022756 | 0.687281846 |
| C3AR1 | 1.221694645 | 0.932697656 |
| TOM1L2 | 0.452707302 | 0.364755292 |
| AKAP9 | 0.357819474 | 0.446521618 |
| ZYG11B | 0.335492213 | 0.436903929 |
| PDLIM2 | 0.433078413 | 0.513401917 |
| RAB11FIP1 | 0.342800412 | 0.527339301 |
| CD244 | 0.807034237 | 0.514357051 |
| SLFN5 | 1.516432134 | 1.492366991 |
| GPR137 | 0.421547566 | 0.578150348 |
| HIPK2 | 0.330923464 | 0.727141182 |
| YPEL3 | 0.521502865 | 1.335678725 |
| CALCOCO1 | 0.370902688 | 0.486639511 |
| ADGRB1 | 0.924347954 | 0.511838214 |
| ATP6V0A1 | 0.394250521 | 0.328040405 |
| SAT1 | 0.412683467 | 0.479306462 |
| SYT11 | 0.416628995 | 0.632954886 |
| KIAA1109 | 0.421602981 | 0.654416986 |
| ADD3 | 0.336428991 | 0.357477818 |
| SRD5A1 | 0.408189245 | 0.655737528 |
| NCOA7 | 0.425373905 | 0.391533614 |
| RNF213 | 0.657762616 | 0.75886797 |
| RCBTB2 | 0.442344218 | 0.510846955 |
| MXD3 | 0.385632497 | 0.491572275 |
| FAM49A | 0.359203064 | 0.628440986 |
| GATS | 0.786452737 | 1.656471268 |
| DPYSL3 | 0.896261707 | 0.680895133 |
| SLC26A11 | 0.819788165 | 4.794263792 |
| TRANK1 | 0.726684399 | 1.310873798 |
| PGAP1 | 0.529706087 | 1.315518698 |
| ATP7A | 0.516784941 | 0.573020726 |
| CLSTN3 | 0.495342391 | 0.427767302 |
| GPR18 | 0.358532637 | 0.456048706 |
| JUP | 1.462260776 | 0.852542223 |
| ARRDC3 | 0.555188435 | 1.067477118 |
| GNG2 | 0.806043054 | 0.486493849 |
| ZFYVE26 | 0.330797044 | 0.469311262 |
| NCF2 | 0.881315902 | 0.811791012 |
| PGM2L1 | 0.688685588 | 0.642482906 |
| TLR6 | 0.66772655 | 0.727490497 |
| TMEM8B | 0.723906022 | 0.860020147 |
| ABCA5 | 0.367513971 | 1.850670448 |
| CLCN5 | 0.408060293 | 0.634924917 |
| PAQR8 | 0.453174445 | 0.857923376 |
| CYTIP | 0.839348561 | 0.719222686 |
| TLN2 | 0.821386102 | 0.438802642 |
| ZFHX3 | 1.096647095 | 0.95481196 |
| EXD2 | 0.412304694 | 0.550006445 |
| C1RL | 0.368459366 | 0.517921031 |
| ARMC9 | 0.42848912 | 0.754766431 |
| CCDC186 | 0.339177733 | 0.515035873 |
| HCN3 | 0.574924524 | 0.632203026 |
| KIF3A | 0.372179667 | 0.89034981 |
| ST3GAL6 | 0.519346075 | 0.921253279 |
| FAM161B | 0.666461066 | 0.736229026 |
| RBMS2 | 0.631748676 | 0.649310526 |

| Gene Name | OCI3_log2 Fold-Change | MOLM_log2 Fold-Change |
|---|---|---|
| PDE4B | 0.352456131 | 0.758254057 |
| LILRB1 | 0.411427086 | 0.452965695 |
| MMP25 | 0.686980358 | 0.540620268 |
| ID3 | 0.824744944 | 1.860681076 |
| PPM1L | 0.576138011 | 0.364537186 |
| TNFSF14 | 0.553042418 | 0.658494302 |
| TCP11L2 | 0.810034588 | 1.378475524 |
| IZUMO4 | 0.625807617 | 0.744764371 |
| DDX60 | 0.441978755 | 0.919900684 |
| IL16 | 0.767902742 | 0.732064488 |
| NEK1 | 0.323725029 | 0.439033443 |
| RAP1GAP2 | 0.507598612 | 0.617935404 |
| IL10RA | 0.390770673 | 1.055185292 |
| PCNX2 | 0.526711998 | 1.243761557 |
| ZNF697 | 0.329821733 | 0.441468191 |
| ZBED6 | 0.435129938 | 0.719370258 |
| DNASE2 | 2.559981297 | 1.063533372 |
| HERC6 | 0.49650297 | 1.603392029 |
| PLXDC2 | 0.612615596 | 1.442011086 |
| WDR31 | 0.387644144 | 0.829895216 |
| SSBP2 | 0.363597643 | 0.795505674 |
| TBC1D2 | 0.522095257 | 0.360127881 |
| CLEC12A | 1.358228349 | 0.745613028 |
| AGPAT4 | 0.766165722 | 1.255886792 |
| TRIM52 | 0.378165 | 0.779334083 |
| ABHD4 | 0.559804822 | 0.436279846 |
| GPR68 | 0.414751909 | 0.990790546 |
| GIPR | 1.685600231 | 0.776034697 |
| PORCN | 0.861686867 | 0.527197971 |
| AHNAK | 0.41599897 | 0.886870709 |
| ATP8A1 | 0.521598808 | 1.129621732 |
| SH2D3C | 1.252554528 | 0.550206054 |
| EFHC1 | 0.352067154 | 0.781076831 |
| LILRA1 | 0.406543436 | 0.869112137 |
| TXNDC16 | 0.350555955 | 0.471645262 |
| LILRB2 | 1.337436627 | 1.752720784 |
| NBEAL1 | 0.40449611 | 0.927040292 |
| SCIMP | 0.451203837 | 1.407058276 |
| CIITA | 0.614624659 | 1.546655712 |
| JAML | 1.478277329 | 0.876956842 |
| ITGAX | 0.533639109 | 1.090570387 |
| PDE1B | 0.463404859 | 0.575854074 |
| AVPR2 | 0.565152461 | 0.918441081 |
| CCDC146 | 0.767119293 | 1.423660585 |
| SP110 | 0.418252735 | 1.024912162 |
| ANKRD36C | 0.419040935 | 0.596539217 |
| SPATC1 | 1.312086081 | 1.796085974 |
| LMTK3 | 0.989395453 | 0.952308957 |
| SPACA6 | 0.386970809 | 1.334299817 |
| MAP3K14 | 0.352575841 | 0.486211771 |
| ABHD8 | 0.421757524 | 0.749295326 |
| TSHZ3 | 0.578717177 | 0.943820211 |
| PRX | 0.423918245 | 0.743940845 |
| SDS | 1.390282554 | 1.737905939 |
| AIM1 | 0.561737512 | 0.445537147 |
| ITPR3 | 0.525410363 | 0.348512757 |
| MTMR11 | 0.355997634 | 0.466360915 |
| TMEM255B | 0.477851949 | 0.723588463 |
| EML6 | 0.610815094 | 2.049094041 |
| RASGRF1 | 1.407975218 | 0.839551044 |
| PILRA | 0.327575984 | 0.758696684 |

Figure S5

**D**

| GeneName | OCI3_log2 FC | MOLM_log2 FC |
|---|---|---|
| CDKN2C | -1.649733229 | -0.957504284 |
| PHF10 | -1.121464064 | -0.570739022 |
| IGF2BP2 | -1.155806612 | -0.679343221 |
| TMEM255A | -2.570378971 | -2.989631346 |
| LAMP5 | -0.845916214 | -0.768378707 |
| IRF8 | -0.749492619 | -0.339881248 |
| SATB1 | -0.680088531 | -0.92339395 |
| CXorf21 | -0.598252328 | -0.545910616 |
| SATB2 | -0.679825687 | -0.600778519 |
| SRGN | -0.553921529 | -0.4168654 |
| FLT3 | -1.056400388 | -0.712988005 |
| CPEB2 | -1.230555823 | -1.809057416 |
| KCNN2 | -0.953083024 | -0.813782437 |
| FOXP1 | -0.750430988 | -0.497603164 |
| TESC | -0.859177611 | -0.610680802 |
| PBX3 | -0.818842026 | -1.768859435 |
| SDC2 | -0.487149636 | -1.067202025 |
| KLHL23 | -0.816710615 | -0.78709842 |
| MYC | -0.351530754 | -0.337674731 |
| REEP3 | -0.581024563 | -1.441970132 |
| TERT | -0.660329416 | -0.324917509 |
| ZNF516 | -0.417680197 | -0.351410952 |
| DLX1 | -0.550081491 | -0.730310543 |
| TFB2M | -0.409087985 | -0.414146039 |
| SLC25A19 | -0.412433634 | -0.377046677 |
| NANOS1 | -1.193674438 | -0.992017796 |
| FAM120AOS | -0.45521356 | -0.363490026 |
| HSPA4L | -0.475301018 | -0.441551735 |
| CENPV | -0.833750069 | -0.586459719 |
| CDK6 | -0.434179181 | -0.457528672 |
| MEIS1 | -0.494833554 | -2.592036446 |
| RRP7A | -0.340158808 | -0.436506155 |
| DCUN1D5 | -0.362309609 | -0.333435683 |
| DNAJA4 | -0.431822443 | -0.440829487 |
| STARD8 | -0.615129477 | -0.362435997 |
| MRTO4 | -0.355331992 | -0.427786232 |
| BATF3 | -0.851320649 | -0.771901131 |
| MRPS34 | -0.435247545 | -0.362801654 |
| CSPG4 | -0.588121242 | -0.645501735 |
| ST3GAL4 | -0.423082118 | -0.354410615 |
| NDUFAF3 | -0.370546953 | -0.637817861 |
| RPL36AL | -0.330113755 | -0.384295181 |
| ZEB2 | -0.373498827 | -0.492659654 |
| BAG2 | -0.58136371 | -0.420149031 |
| WDR4 | -0.383034064 | -0.465727625 |
| SFMBT1 | -0.637262679 | -0.345642443 |
| CGREF1 | -0.428232511 | -0.588302825 |
| HS3ST1 | -0.520323269 | -0.941612428 |
| SFXN4 | -0.365622965 | -0.621281327 |
| PLD6 | -0.387615714 | -0.437421361 |

| GeneName | OCI3_log2 FC | MOLM_log2 FC |
|---|---|---|
| PSMG1 | -0.323615833 | -0.424594781 |
| AGMAT | -0.689972746 | -0.333246665 |
| TEAD4 | -0.363515217 | -0.333273264 |
| NOP16 | -0.333407476 | -0.340139849 |
| ZBTB33 | -0.323060432 | -0.606023325 |
| CMTM7 | -0.418332454 | -0.53578187 |
| PIWIL4 | -0.458460044 | -0.450316448 |
| LHX6 | -0.602949475 | -0.506947065 |
| GAREM2 | -0.471599636 | -0.418863173 |
| TIFAB | -1.283306613 | -0.671791898 |
| IL15RA | -0.485748542 | -0.446254007 |
| CSPG5 | -0.580692098 | -1.920972963 |
| EGR1 | -0.356252851 | -1.346300897 |
| AVEN | -0.473762414 | -0.33768645 |
| LRFN1 | -0.389552782 | -0.369222456 |
| CHST10 | -0.325282393 | -0.360801176 |
| SAP30 | -0.436849801 | -0.44226863 |
| QPRT | -0.569628604 | -0.504319731 |
| RAB33A | -0.624997086 | -0.422864464 |
| MYO6 | -0.33596582 | -2.777965996 |
| BEND3 | -0.409590254 | -0.411459062 |
| AP1S3 | -0.622053183 | -0.397109917 |
| CHRM4 | -0.801624196 | -4.094642204 |
| PPP1R27 | -0.606765019 | -0.872440061 |
| RHOBTB3 | -0.351258823 | -0.414934525 |
| C16orf91 | -0.323351116 | -0.349866938 |
| IGFBP7 | -0.39575928 | -0.369522524 |
| RNF157 | -0.44924468 | -0.352607549 |
| FAM89A | -0.447227041 | -0.519579501 |
| FAM127C | -0.364217964 | -0.379933983 |
| KCNQ4 | -0.828723286 | -0.708966915 |
| RGS9BP | -0.670167973 | -1.04644981 |
| TBC1D30 | -0.759982571 | -1.106274122 |
| SOCS2 | -0.896893553 | -2.410567303 |
| HACD1 | -0.519382061 | -0.463960157 |
| MRPS28 | -0.331196482 | -0.522708566 |
| SHOX2 | -0.496676938 | -1.594606875 |
| PPP1R3G | -0.559960037 | -0.629169284 |
| PROK2 | -1.527030596 | -0.542269701 |
| CABLES1 | -0.459216227 | -0.5528942 |
| TOMM5 | -0.366123264 | -0.533577587 |
| GAL | -0.454234218 | -0.473886456 |
| ITPRIPL1 | -0.337822683 | -0.488668089 |
| GET4 | -0.756694731 | -0.454602228 |
| EMID1 | -0.394457229 | -0.629347219 |
| TXNDC5 | -0.530907719 | -0.53845581 |
| DLX2 | -0.470491862 | -0.985056205 |
| GADD45GIP1 | -0.339853646 | -0.3401186 |
| FAM174B | -0.578203186 | -0.4703284 |

**Figure S5**

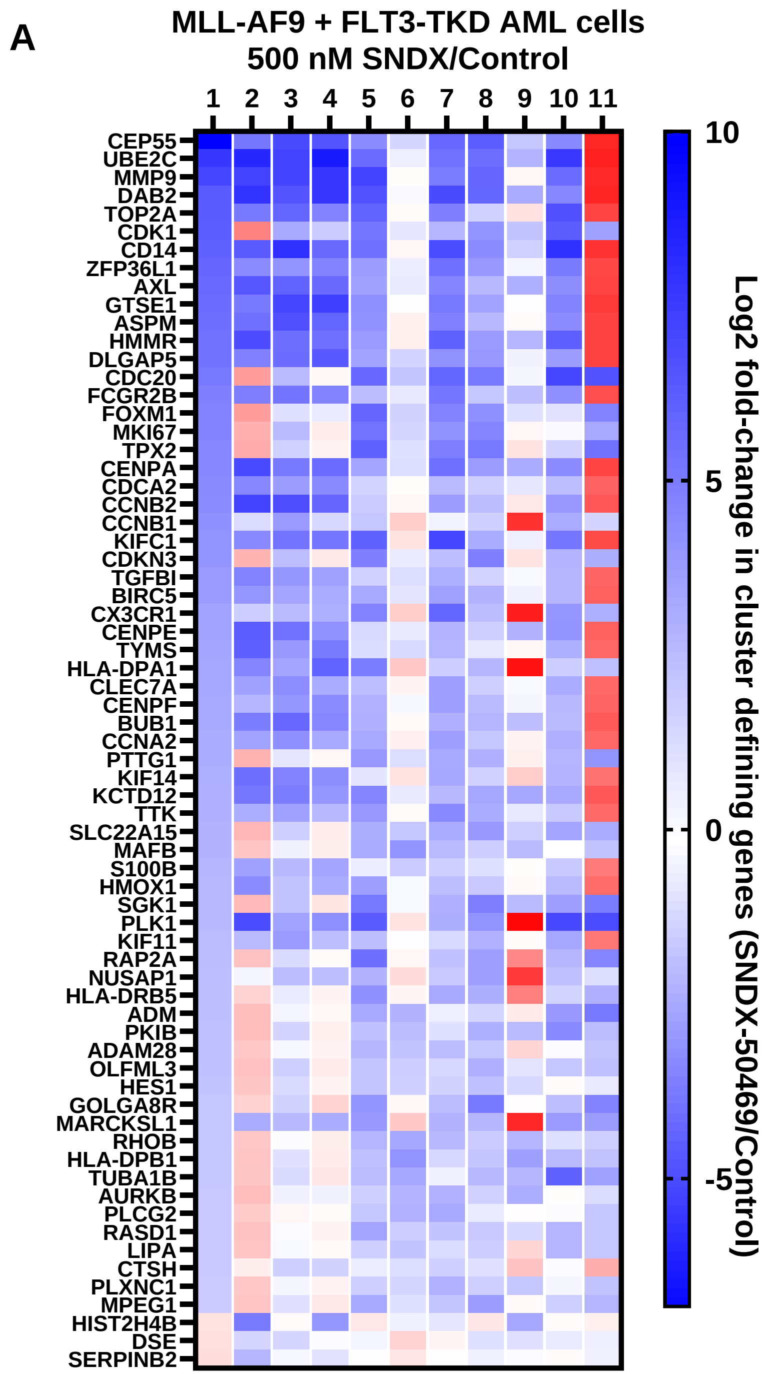
A
MLL-AF9 + FLT3-TKD AML cells
500 nM SNDX/Control
1 2 3 4 5 6 7 8 9 10 11
CEP55
UBE2C
MMP9
DAB2
TOP2A
CDK1
CD14
ZFP36L1
AXL
GTSE1
ASPM
HMMR
DLGAP5
CDC20
FCGR2B
FOXM1
MKI67
TPX2
CENPA
CDCA2
CCNB2
CCNB1
KIFC1
CDKN3
TGFBI
BIRC5
CX3CR1
CENPE
TYMS
HLA-DPA1
CLEC7A
CENPF
BUB1
CCNA2
PTTG1
KIF14
KCTD12
TTK
SLC22A15
MAFB
S100B
HMOX1
SGK1
PLK1
KIF11
RAP2A
NUSAP1
HLA-DRB5
ADM
PKIB
ADAM28
OLFML3
HES1
GOLGA8R
MARCKSL1
RHOB
HLA-DPB1
TUBA1B
AURKB
PLCG2
RASD1
LIPA
CTSH
PLXNC1
MPEG1
HIST2H4B
DSE
SERPINB2
10
5
0
-5
Log2 fold-change in cluster defining genes (SNDX-50469/Control)

Figure S6

B

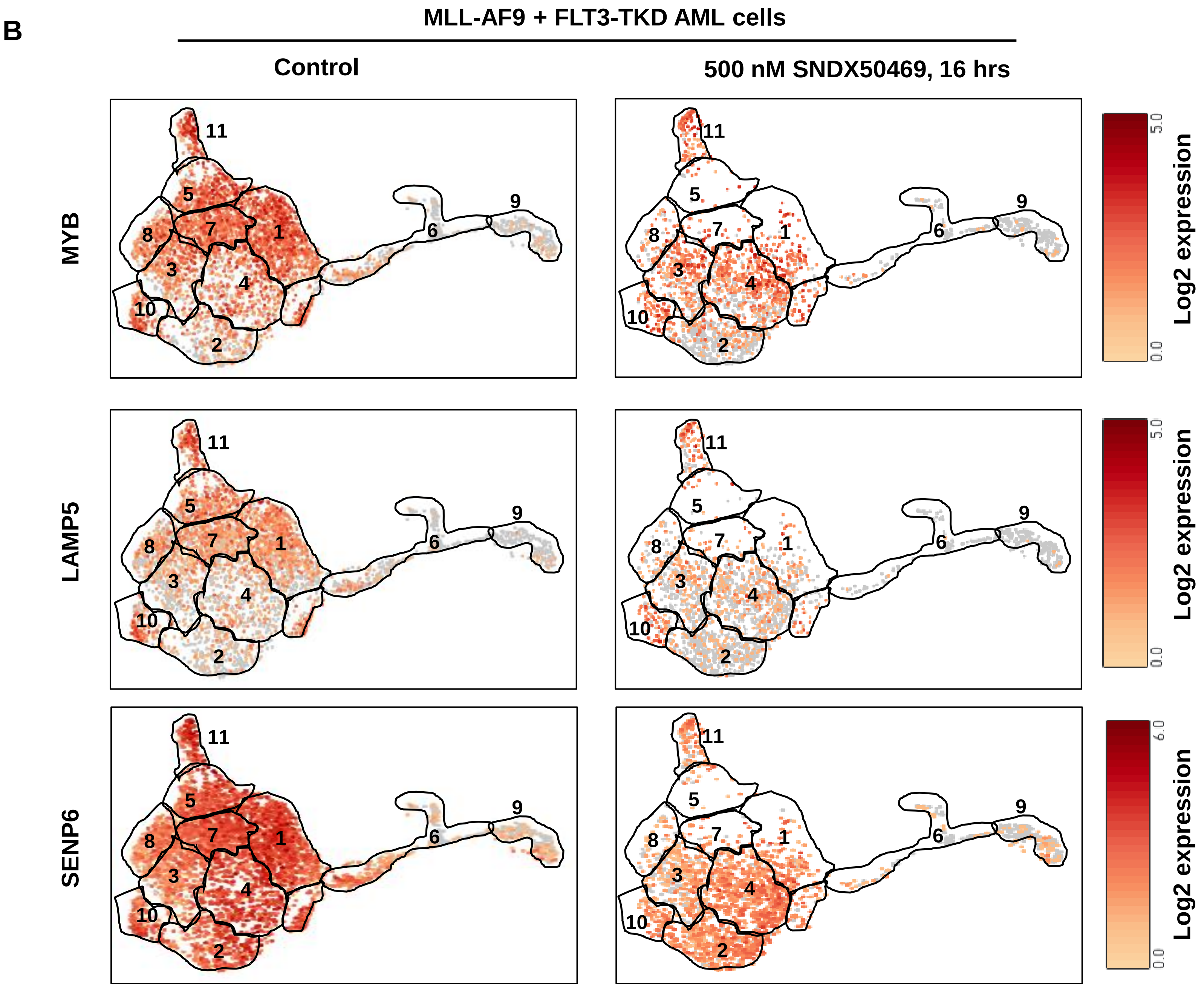
MLL-AF9 + FLT3-TKD AML cells
Control
500 nM SNDX50469, 16 hrs
MYB
LAMP5
SENP6
Log2 expression
5.0
0.0
6.0
11
5
8
7
1
3
4
10
2
6
9

Figure S6

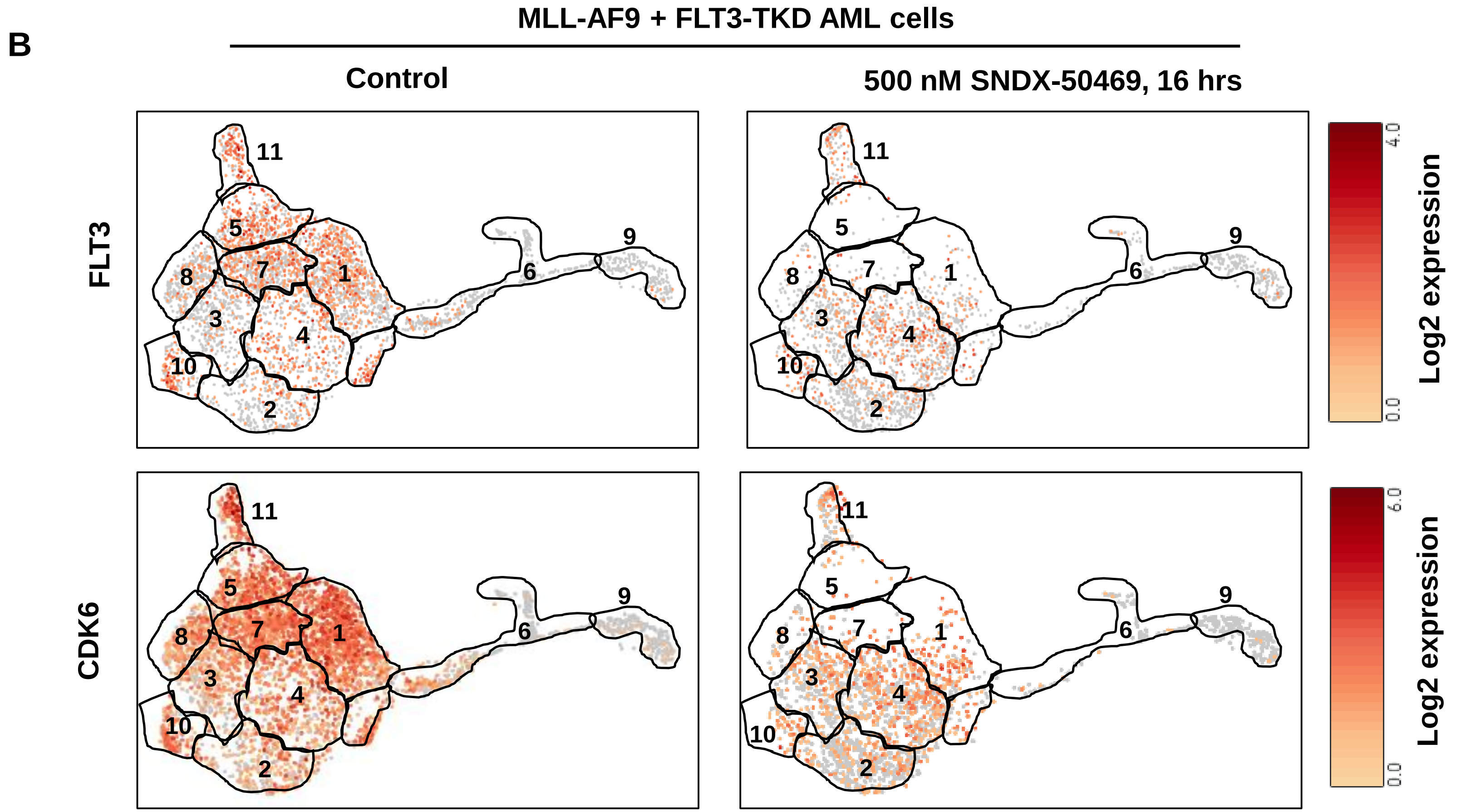
B
MLL-AF9 + FLT3-TKD AML cells
Control
500 nM SNDX-50469, 16 hrs
FLT3
CDK6
11
5
7
1
8
3
4
10
2
6
9
Log2 expression
4.0
0.0
6.0

Figure S6

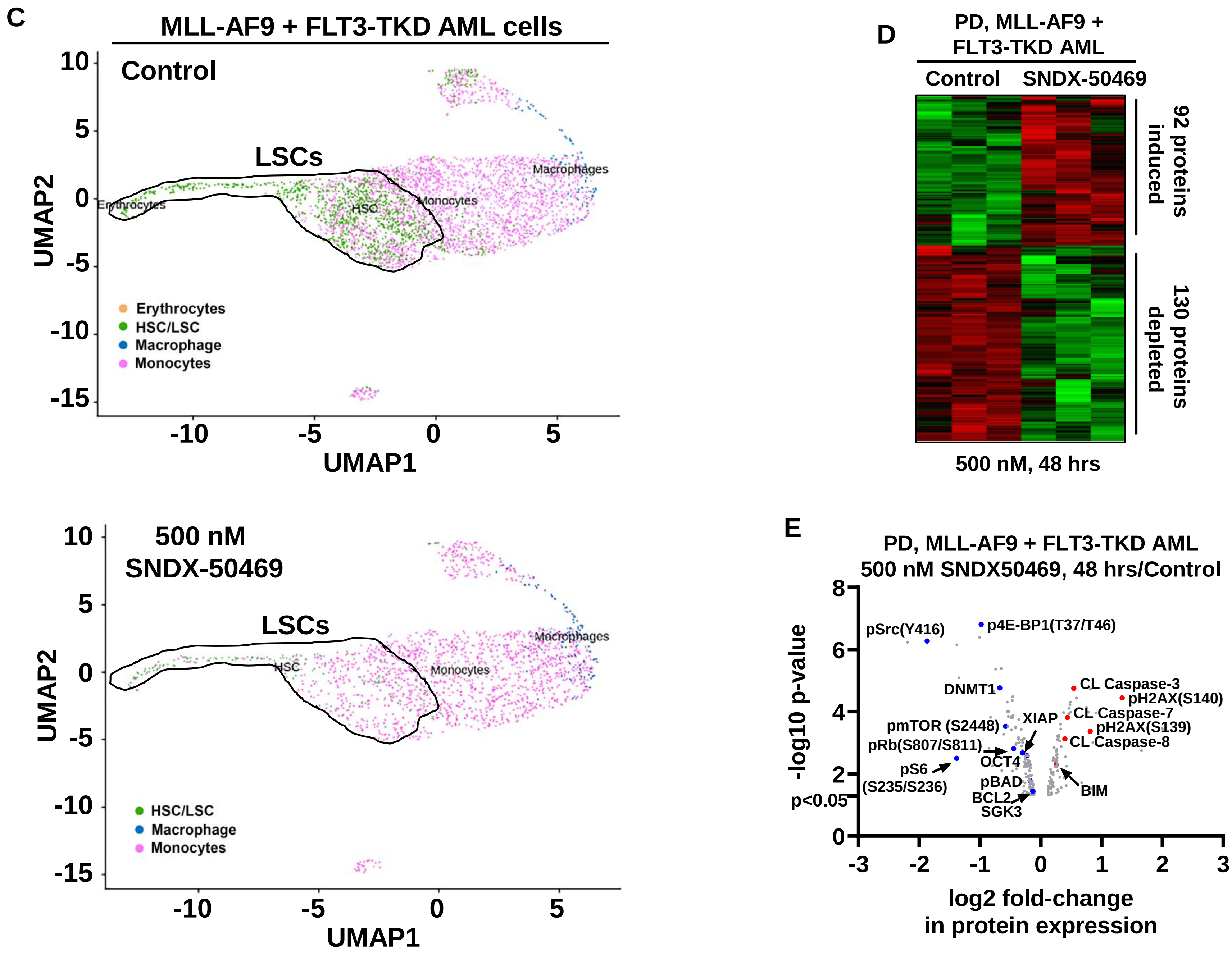


SNDX-50469 treatment decreases the # of cells with an HSC/LSC mRNA signature.

Figure S6

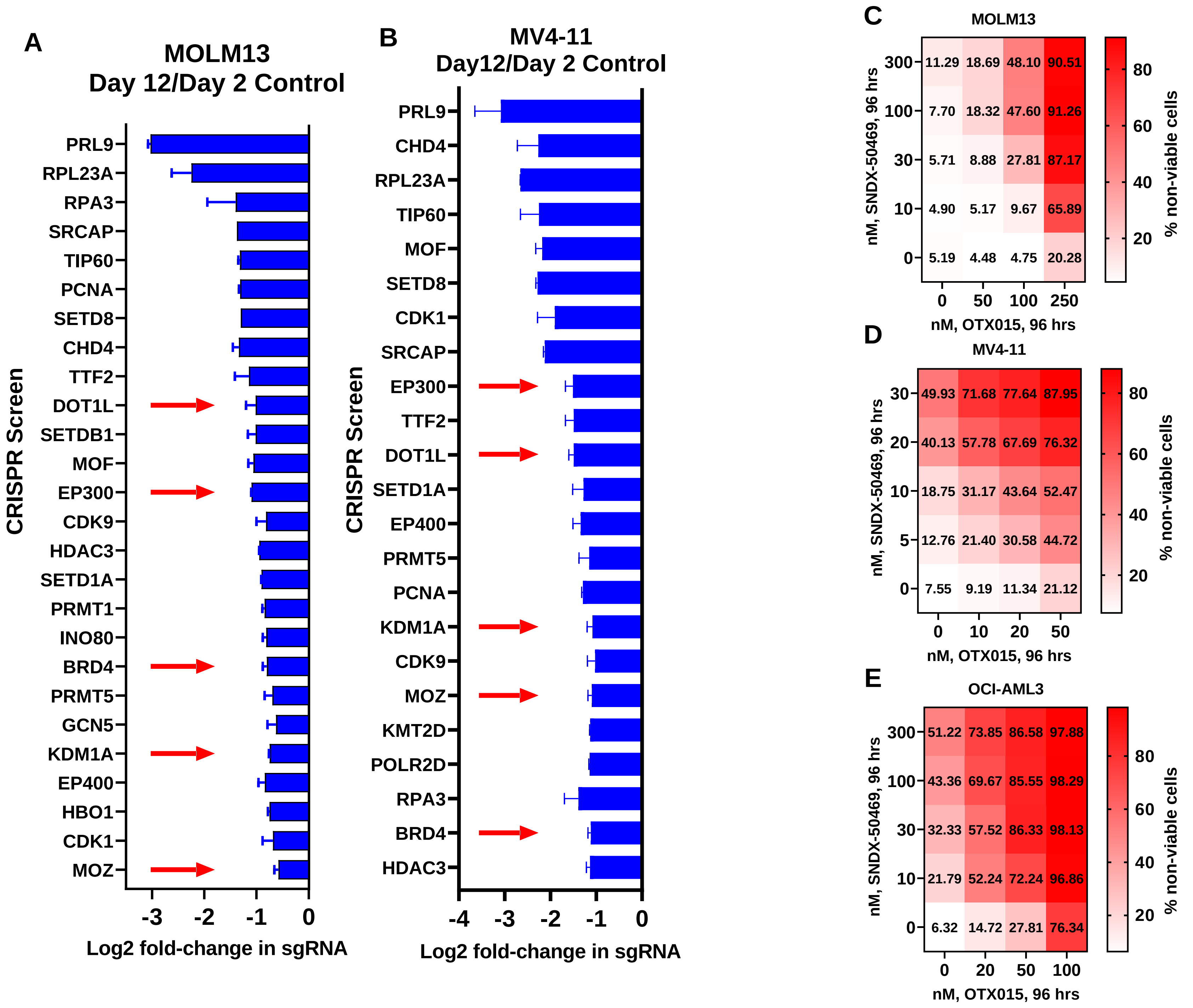
A
MOLM13
Day 12/Day 2 Control
PRL9
RPL23A
RPA3
SRCAP
TIP60
PCNA
SETD8
CHD4
TTF2
DOT1L
SETDB1
MOF
EP300
CDK9
HDAC3
SETD1A
PRMT1
INO80
BRD4
PRMT5
GCN5
KDM1A
EP400
HBO1
CDK1
MOZ
CRISPR Screen
-3 -2 -1 0
Log2 fold-change in sgRNA
B
MV4-11
Day12/Day 2 Control
PRL9
CHD4
RPL23A
TIP60
MOF
SETD8
CDK1
SRCAP
EP300
TTF2
DOT1L
SETD1A
EP400
PRMT5
PCNA
KDM1A
CDK9
MOZ
KMT2D
POLR2D
RPA3
BRD4
HDAC3
CRISPR Screen
-4 -3 -2 -1 0
Log2 fold-change in sgRNA
C
MOLM13
300 11.29 18.69 48.10 90.51
100 7.70 18.32 47.60 91.26
30 5.71 8.88 27.81 87.17
10 4.90 5.17 9.67 65.89
0 5.19 4.48 4.75 20.28
0 50 100 250
nM, SNDX-50469, 96 hrs
nM, OTX015, 96 hrs
% non-viable cells
20 40 60 80
D
MV4-11
30 49.93 71.68 77.64 87.95
20 40.13 57.78 67.69 76.32
10 18.75 31.17 43.64 52.47
5 12.76 21.40 30.58 44.72
0 7.55 9.19 11.34 21.12
0 10 20 50
nM, SNDX-50469, 96 hrs
nM, OTX015, 96 hrs
% non-viable cells
20 40 60 80
E
OCI-AML3
300 51.22 73.85 86.58 97.88
100 43.36 69.67 85.55 98.29
30 32.33 57.52 86.33 98.13
10 21.79 52.24 72.24 96.86
0 6.32 14.72 27.81 76.34
0 20 50 100
nM, SNDX-50469, 96 hrs
nM, OTX015, 96 hrs
% non-viable cells
20 40 60 80

Figure S7

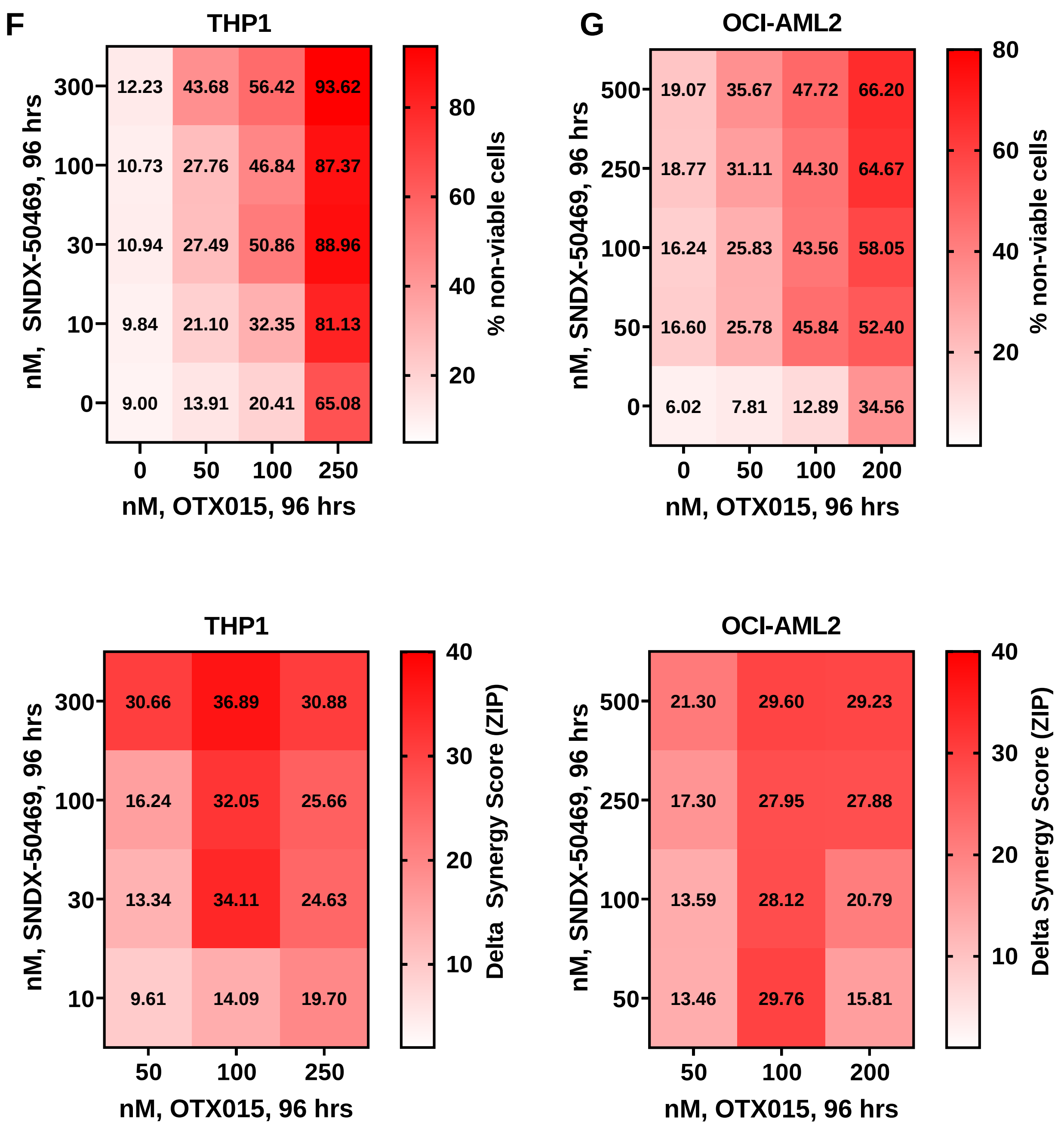
F
THP1
300 12.23 43.68 56.42 93.62
100 10.73 27.76 46.84 87.37
30 10.94 27.49 50.86 88.96
10 9.84 21.10 32.35 81.13
0 9.00 13.91 20.41 65.08
0 50 100 250
nM, OTX015, 96 hrs
nM, SNDX-50469, 96 hrs
20 40 60 80
% non-viable cells
G
OCI-AML2
500 19.07 35.67 47.72 66.20
250 18.77 31.11 44.30 64.67
100 16.24 25.83 43.56 58.05
50 16.60 25.78 45.84 52.40
0 6.02 7.81 12.89 34.56
0 50 100 200
nM, OTX015, 96 hrs
nM, SNDX-50469, 96 hrs
20 40 60 80
% non-viable cells
THP1
300 30.66 36.89 30.88
100 16.24 32.05 25.66
30 13.34 34.11 24.63
10 9.61 14.09 19.70
50 100 250
nM, OTX015, 96 hrs
nM, SNDX-50469, 96 hrs
10 20 30 40
Delta Synergy Score (ZIP)
OCI-AML2
500 21.30 29.60 29.23
250 17.30 27.95 27.88
100 13.59 28.12 20.79
50 13.46 29.76 15.81
50 100 200
nM, OTX015, 96 hrs
nM, SNDX-50469, 96 hrs
10 20 30 40
Delta Synergy Score (ZIP)

**Figure S7**

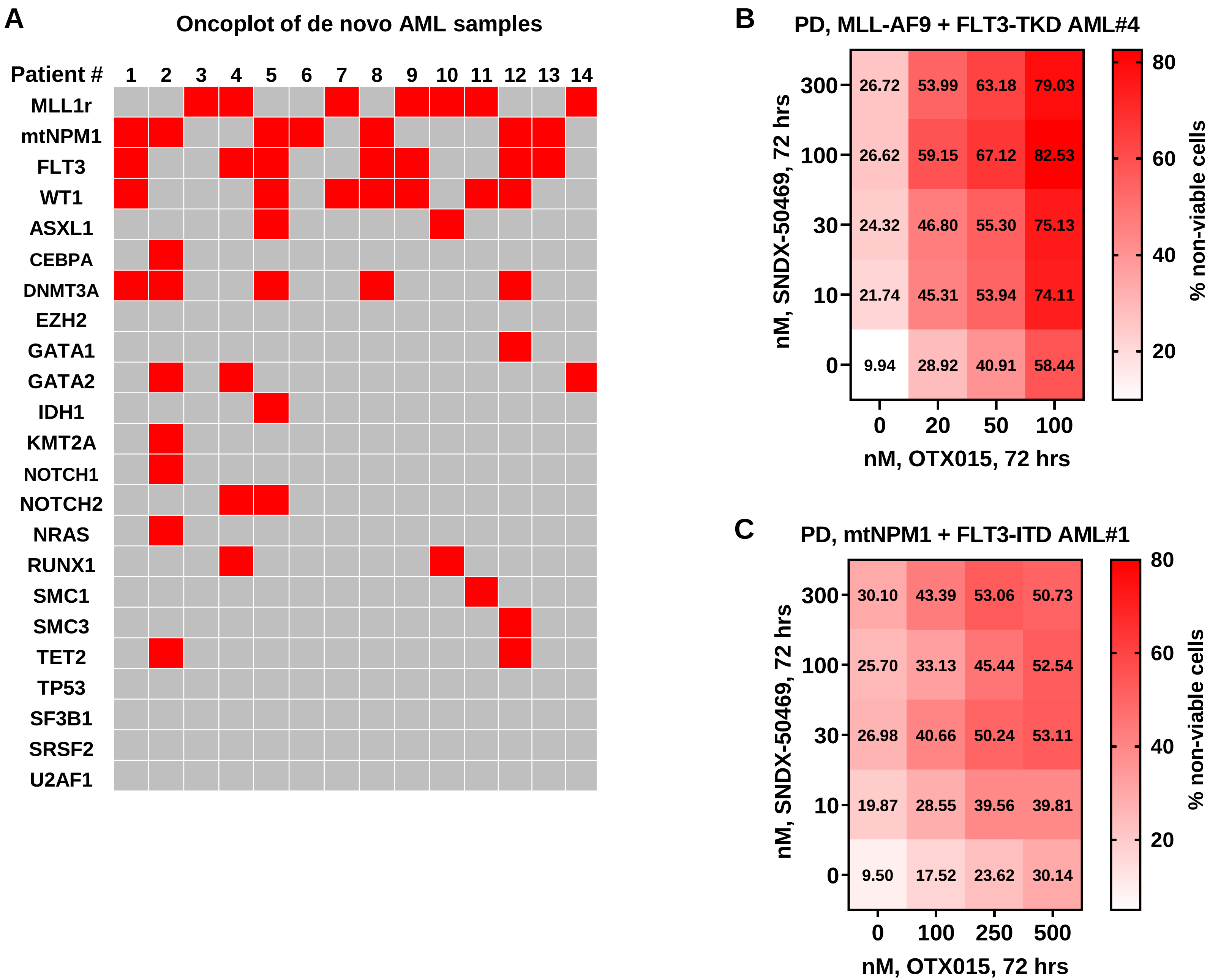
A
Oncoplot of de novo AML samples
Patient #
1 2 3 4 5 6 7 8 9 10 11 12 13 14
MLL1r
mtNPM1
FLT3
WT1
ASXL1
CEBPA
DNMT3A
EZH2
GATA1
GATA2
IDH1
KMT2A
NOTCH1
NOTCH2
NRAS
RUNX1
SMC1
SMC3
TET2
TP53
SF3B1
SRSF2
U2AF1
B
PD, MLL-AF9 + FLT3-TKD AML#4
300 26.72 53.99 63.18 79.03
100 26.62 59.15 67.12 82.53
30 24.32 46.80 55.30 75.13
10 21.74 45.31 53.94 74.11
0 9.94 28.92 40.91 58.44
0 20 50 100
nM, OTX015, 72 hrs
nM, SNDX-50469, 72 hrs
% non-viable cells
80 60 40 20
C
PD, mtNPM1 + FLT3-ITD AML#1
300 30.10 43.39 53.06 50.73
100 25.70 33.13 45.44 52.54
30 26.98 40.66 50.24 53.11
10 19.87 28.55 39.56 39.81
0 9.50 17.52 23.62 30.14
0 100 250 500
nM, OTX015, 72 hrs
nM, SNDX-50469, 72 hrs
% non-viable cells
80 60 40 20

Figure S8

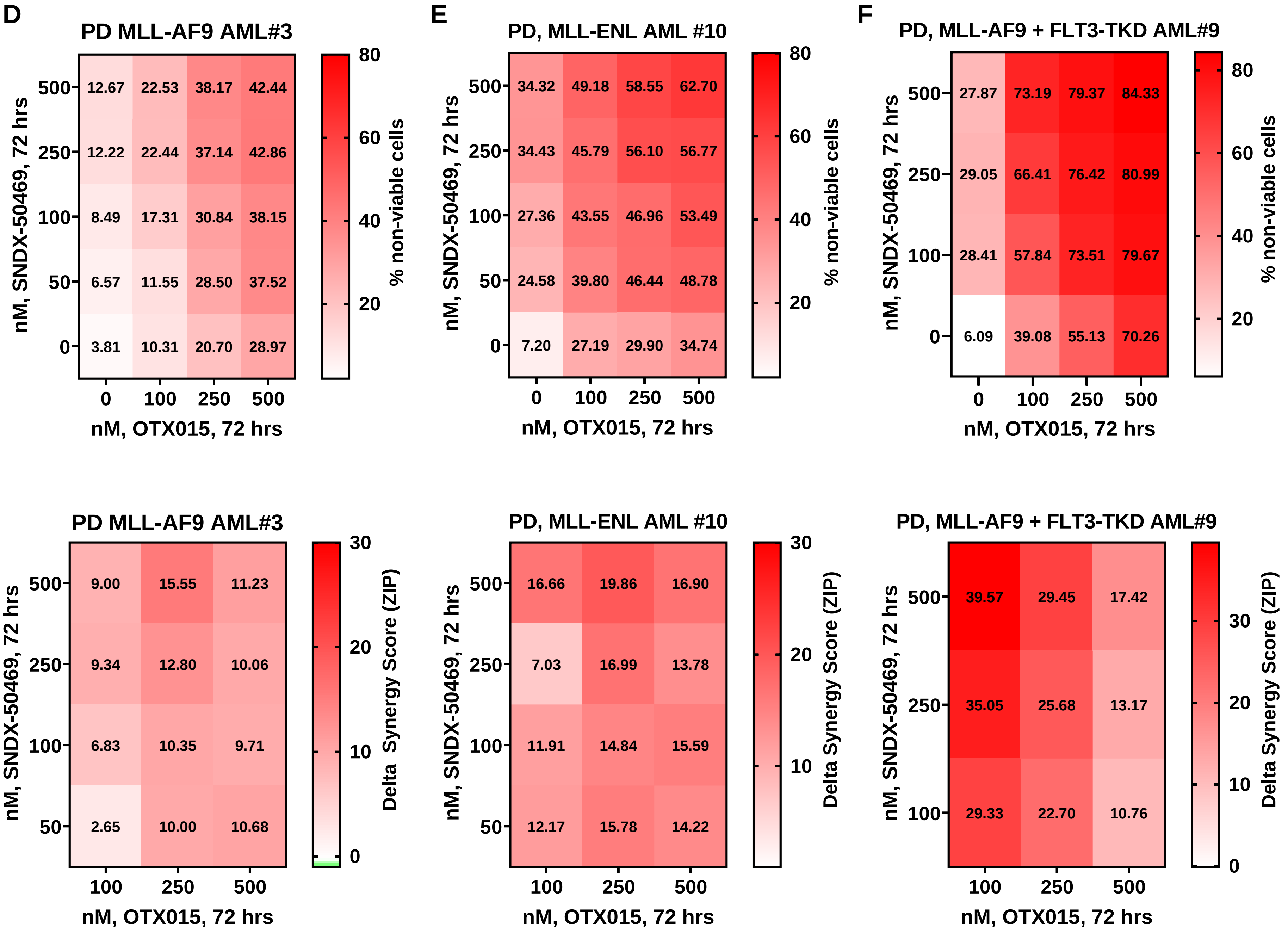

D
PD MLL-AF9 AML#3
500 12.67 22.53 38.17 42.44
250 12.22 22.44 37.14 42.86
100 8.49 17.31 30.84 38.15
50 6.57 11.55 28.50 37.52
0 3.81 10.31 20.70 28.97
0 100 250 500
nM, OTX015, 72 hrs
nM, SNDX-50469, 72 hrs
80 60 40 20
% non-viable cells
E
PD, MLL-ENL AML #10
500 34.32 49.18 58.55 62.70
250 34.43 45.79 56.10 56.77
100 27.36 43.55 46.96 53.49
50 24.58 39.80 46.44 48.78
0 7.20 27.19 29.90 34.74
0 100 250 500
nM, OTX015, 72 hrs
nM, SNDX-50469, 72 hrs
80 60 40 20
% non-viable cells
F
PD, MLL-AF9 + FLT3-TKD AML#9
500 27.87 73.19 79.37 84.33
250 29.05 66.41 76.42 80.99
100 28.41 57.84 73.51 79.67
0 6.09 39.08 55.13 70.26
0 100 250 500
nM, OTX015, 72 hrs
nM, SNDX-50469, 72 hrs
80 60 40 20
% non-viable cells
PD MLL-AF9 AML#3
500 9.00 15.55 11.23
250 9.34 12.80 10.06
100 6.83 10.35 9.71
50 2.65 10.00 10.68
100 250 500
nM, OTX015, 72 hrs
nM, SNDX-50469, 72 hrs
30 20 10 0
Delta Synergy Score (ZIP)
PD, MLL-ENL AML #10
500 16.66 19.86 16.90
250 7.03 16.99 13.78
100 11.91 14.84 15.59
50 12.17 15.78 14.22
100 250 500
nM, OTX015, 72 hrs
nM, SNDX-50469, 72 hrs
30 20 10
Delta Synergy Score (ZIP)
PD, MLL-AF9 + FLT3-TKD AML#9
500 39.57 29.45 17.42
250 35.05 25.68 13.17
100 29.33 22.70 10.76
100 250 500
nM, OTX015, 72 hrs
nM, SNDX-50469, 72 hrs
30 20 10 0
Delta Synergy Score (ZIP)


**Figure S8**

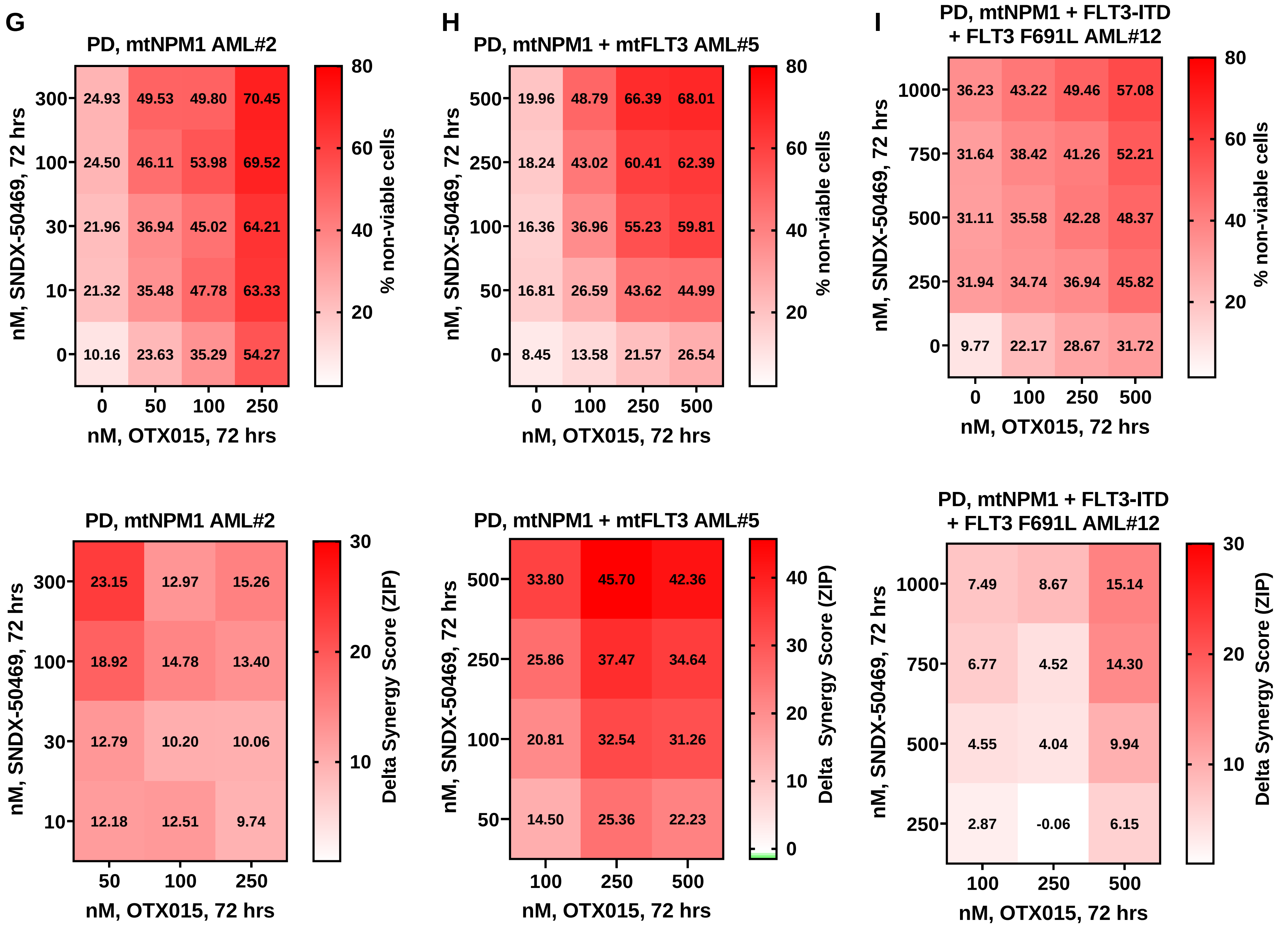

G
PD, mtNPM1 AML#2
nM, SNDX-50469, 72 hrs
300 100 30 10 0
24.93 49.53 49.80 70.45
24.50 46.11 53.98 69.52
21.96 36.94 45.02 64.21
21.32 35.48 47.78 63.33
10.16 23.63 35.29 54.27
0 50 100 250
nM, OTX015, 72 hrs
80 60 40 20
% non-viable cells
H
PD, mtNPM1 + mtFLT3 AML#5
nM, SNDX-50469, 72 hrs
500 250 100 50 0
19.96 48.79 66.39 68.01
18.24 43.02 60.41 62.39
16.36 36.96 55.23 59.81
16.81 26.59 43.62 44.99
8.45 13.58 21.57 26.54
0 100 250 500
nM, OTX015, 72 hrs
80 60 40 20
% non-viable cells
I
PD, mtNPM1 + FLT3-ITD
+ FLT3 F691L AML#12
nM, SNDX-50469, 72 hrs
1000 750 500 250 0
36.23 43.22 49.46 57.08
31.64 38.42 41.26 52.21
31.11 35.58 42.28 48.37
31.94 34.74 36.94 45.82
9.77 22.17 28.67 31.72
0 100 250 500
nM, OTX015, 72 hrs
80 60 40 20
% non-viable cells
PD, mtNPM1 AML#2
nM, SNDX-50469, 72 hrs
300 100 30 10
23.15 12.97 15.26
18.92 14.78 13.40
12.79 10.20 10.06
12.18 12.51 9.74
50 100 250
nM, OTX015, 72 hrs
30 20 10
Delta Synergy Score (ZIP)
PD, mtNPM1 + mtFLT3 AML#5
nM, SNDX-50469, 72 hrs
500 250 100 50
33.80 45.70 42.36
25.86 37.47 34.64
20.81 32.54 31.26
14.50 25.36 22.23
100 250 500
nM, OTX015, 72 hrs
40 30 20 10 0
Delta Synergy Score (ZIP)
PD, mtNPM1 + FLT3-ITD
+ FLT3 F691L AML#12
nM, SNDX-50469, 72 hrs
1000 750 500 250
7.49 8.67 15.14
6.77 4.52 14.30
4.55 4.04 9.94
2.87 -0.06 6.15
100 250 500
nM, OTX015, 72 hrs
30 20 10
Delta Synergy Score (ZIP)


**Figure S8**

J

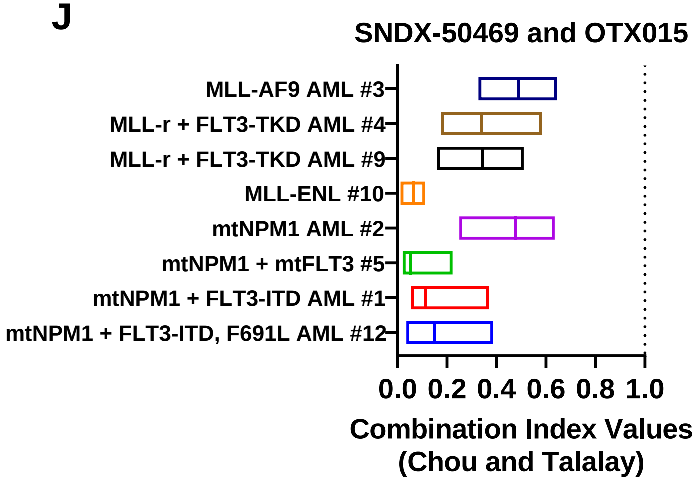
SNDX-50469 and OTX015
MLL-AF9 AML #3
MLL-r + FLT3-TKD AML #4
MLL-r + FLT3-TKD AML #9
MLL-ENL #10
mtNPM1 AML #2
mtNPM1 + mtFLT3 #5
mtNPM1 + FLT3-ITD AML #1
mtNPM1 + FLT3-ITD, F691L AML #12
0.0
0.2
0.4
0.6
0.8
1.0
Combination Index Values
(Chou and Talalay)

Figure S8

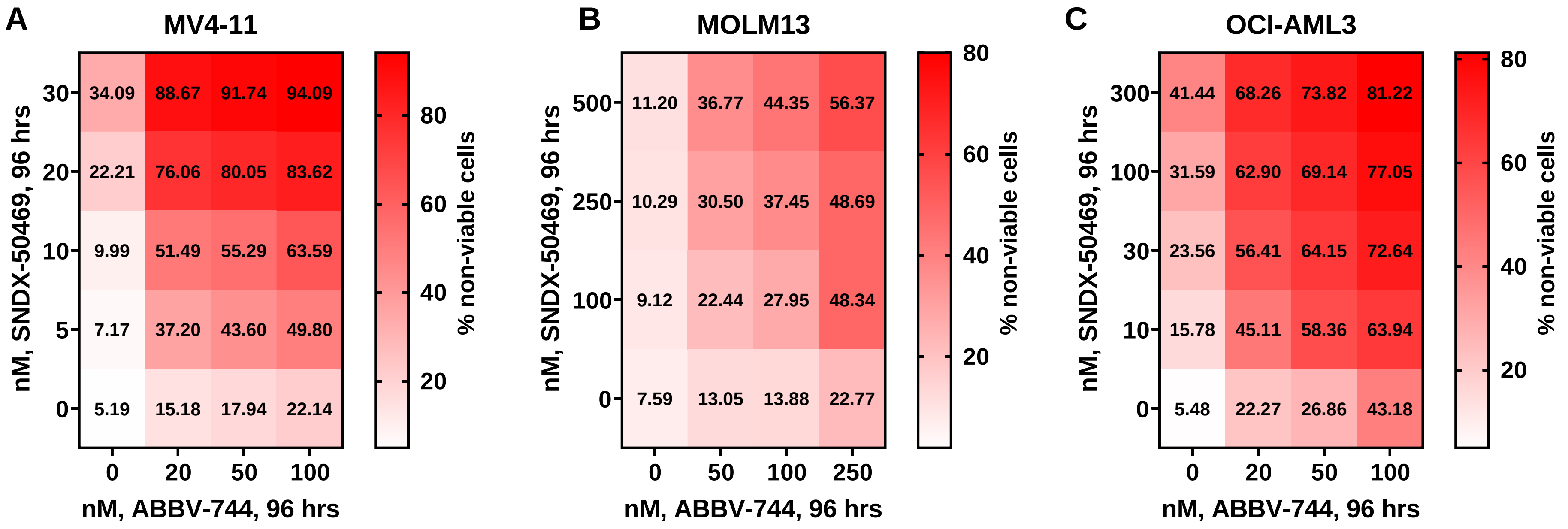
A
MV4-11
nM, SNDX-50469, 96 hrs
30
20
10
5
0
34.09
88.67
91.74
94.09
22.21
76.06
80.05
83.62
9.99
51.49
55.29
63.59
7.17
37.20
43.60
49.80
5.19
15.18
17.94
22.14
0
20
50
100
nM, ABBV-744, 96 hrs
80
60
40
20
% non-viable cells
B
MOLM13
nM, SNDX-50469, 96 hrs
500
250
100
0
11.20
36.77
44.35
56.37
10.29
30.50
37.45
48.69
9.12
22.44
27.95
48.34
7.59
13.05
13.88
22.77
0
50
100
250
nM, ABBV-744, 96 hrs
80
60
40
20
% non-viable cells
C
OCI-AML3
nM, SNDX-50469, 96 hrs
300
100
30
10
0
41.44
68.26
73.82
81.22
31.59
62.90
69.14
77.05
23.56
56.41
64.15
72.64
15.78
45.11
58.36
63.94
5.48
22.27
26.86
43.18
0
20
50
100
nM, ABBV-744, 96 hrs
80
60
40
20
% non-viable cells

Figure S9

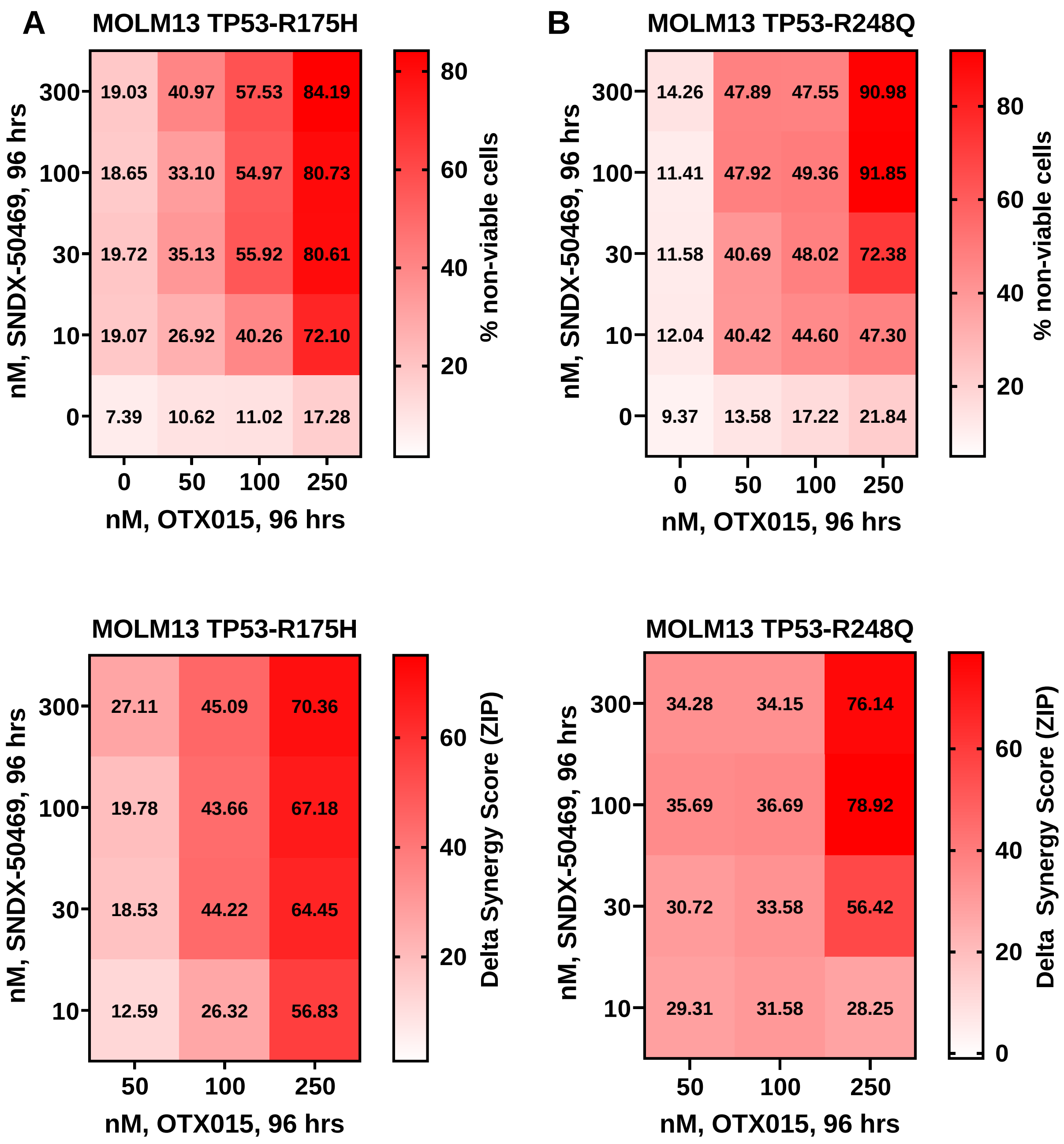
A
MOLM13 TP53-R175H
nM, SNDX-50469, 96 hrs
300
100
30
10
0
19.03 40.97 57.53 84.19
18.65 33.10 54.97 80.73
19.72 35.13 55.92 80.61
19.07 26.92 40.26 72.10
7.39 10.62 11.02 17.28
0 50 100 250
nM, OTX015, 96 hrs
% non-viable cells
80
60
40
20
B
MOLM13 TP53-R248Q
14.26 47.89 47.55 90.98
11.41 47.92 49.36 91.85
11.58 40.69 48.02 72.38
12.04 40.42 44.60 47.30
9.37 13.58 17.22 21.84
MOLM13 TP53-R175H
27.11 45.09 70.36
19.78 43.66 67.18
18.53 44.22 64.45
12.59 26.32 56.83
50 100 250
Delta Synergy Score (ZIP)
60
40
20
MOLM13 TP53-R248Q
34.28 34.15 76.14
35.69 36.69 78.92
30.72 33.58 56.42
29.31 31.58 28.25
0

Figure S10

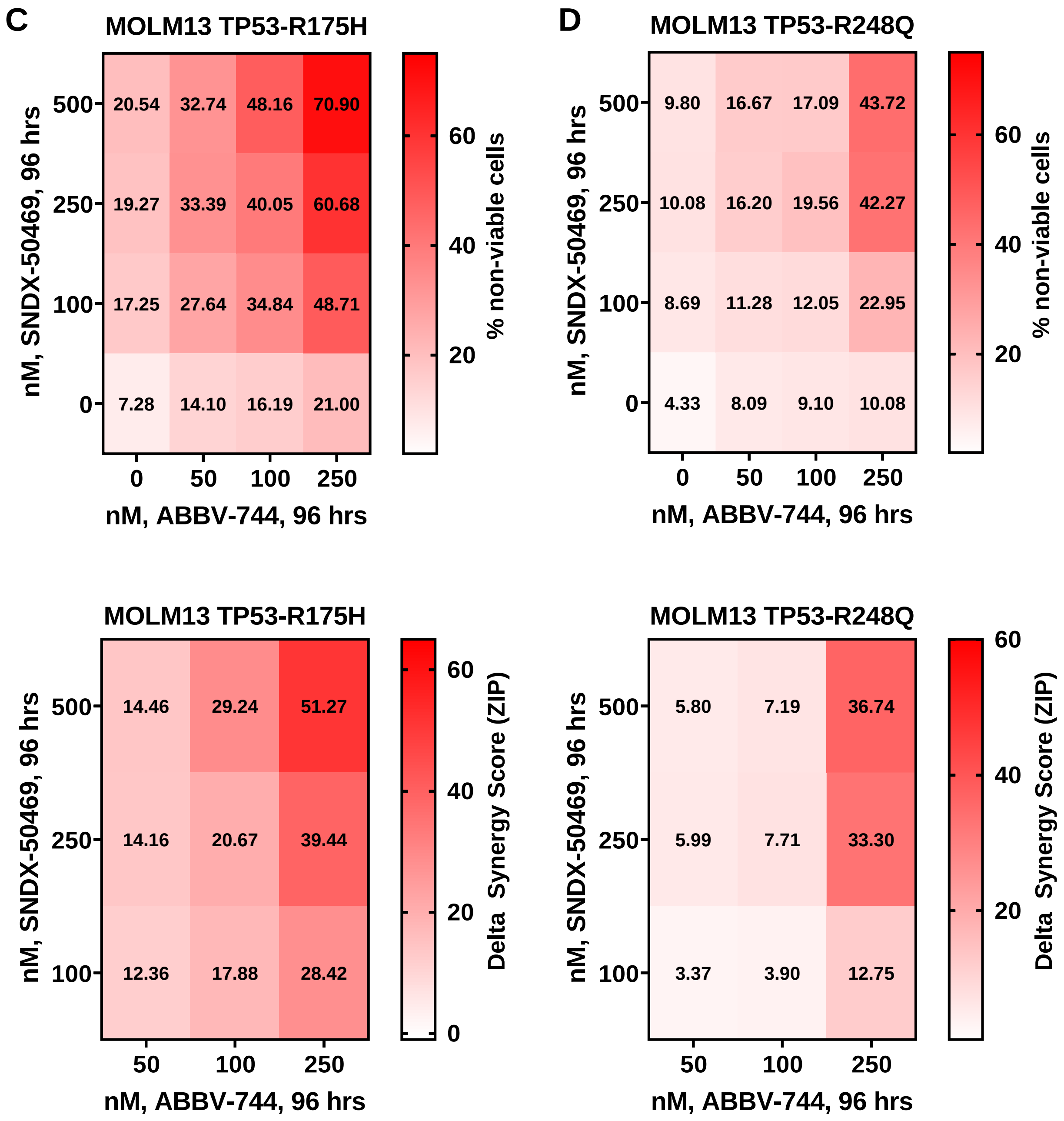
C
MOLM13 TP53-R175H
nM, SNDX-50469, 96 hrs
500
250
100
0
20.54
32.74
48.16
70.90
19.27
33.39
40.05
60.68
17.25
27.64
34.84
48.71
7.28
14.10
16.19
21.00
0
50
100
250
nM, ABBV-744, 96 hrs
% non-viable cells
60
40
20
D
MOLM13 TP53-R248Q
9.80
16.67
17.09
43.72
10.08
16.20
19.56
42.27
8.69
11.28
12.05
22.95
4.33
8.09
9.10
10.08
MOLM13 TP53-R175H
14.46
29.24
51.27
14.16
20.67
39.44
12.36
17.88
28.42
Delta Synergy Score (ZIP)
0
MOLM13 TP53-R248Q
5.80
7.19
36.74
5.99
7.71
33.30
3.37
3.90
12.75

Figure S10

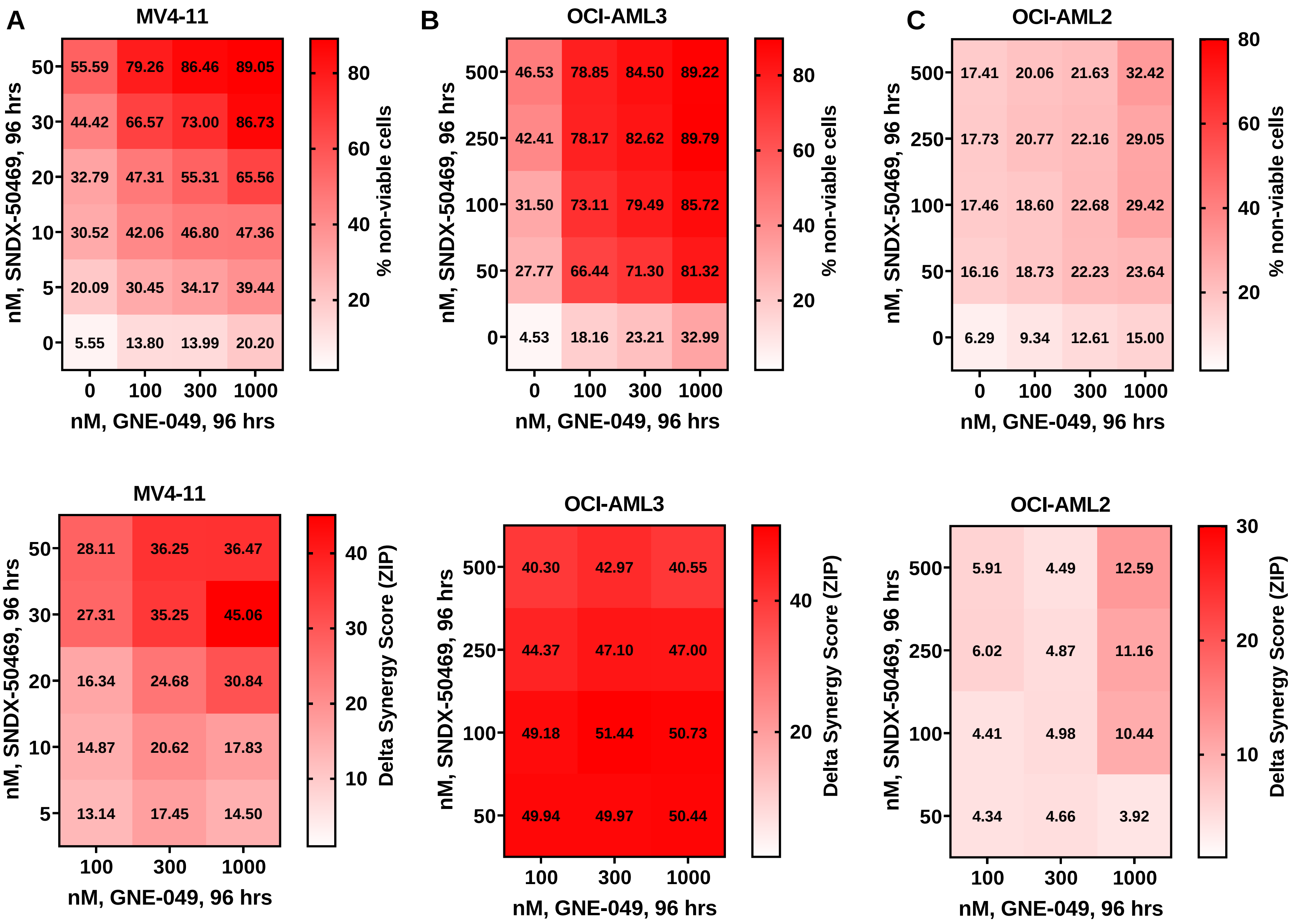
A
MV4-11
B
OCI-AML3
C
OCI-AML2
nM, SNDX-50469, 96 hrs
nM, GNE-049, 96 hrs
% non-viable cells
Delta Synergy Score (ZIP)
55.59 79.26 86.46 89.05
44.42 66.57 73.00 86.73
32.79 47.31 55.31 65.56
30.52 42.06 46.80 47.36
20.09 30.45 34.17 39.44
5.55 13.80 13.99 20.20
46.53 78.85 84.50 89.22
42.41 78.17 82.62 89.79
31.50 73.11 79.49 85.72
27.77 66.44 71.30 81.32
4.53 18.16 23.21 32.99
17.41 20.06 21.63 32.42
17.73 20.77 22.16 29.05
17.46 18.60 22.68 29.42
16.16 18.73 22.23 23.64
6.29 9.34 12.61 15.00
28.11 36.25 36.47
27.31 35.25 45.06
16.34 24.68 30.84
14.87 20.62 17.83
13.14 17.45 14.50
40.30 42.97 40.55
44.37 47.10 47.00
49.18 51.44 50.73
49.94 49.97 50.44
5.91 4.49 12.59
6.02 4.87 11.16
4.41 4.98 10.44
4.34 4.66 3.92

Figure S11

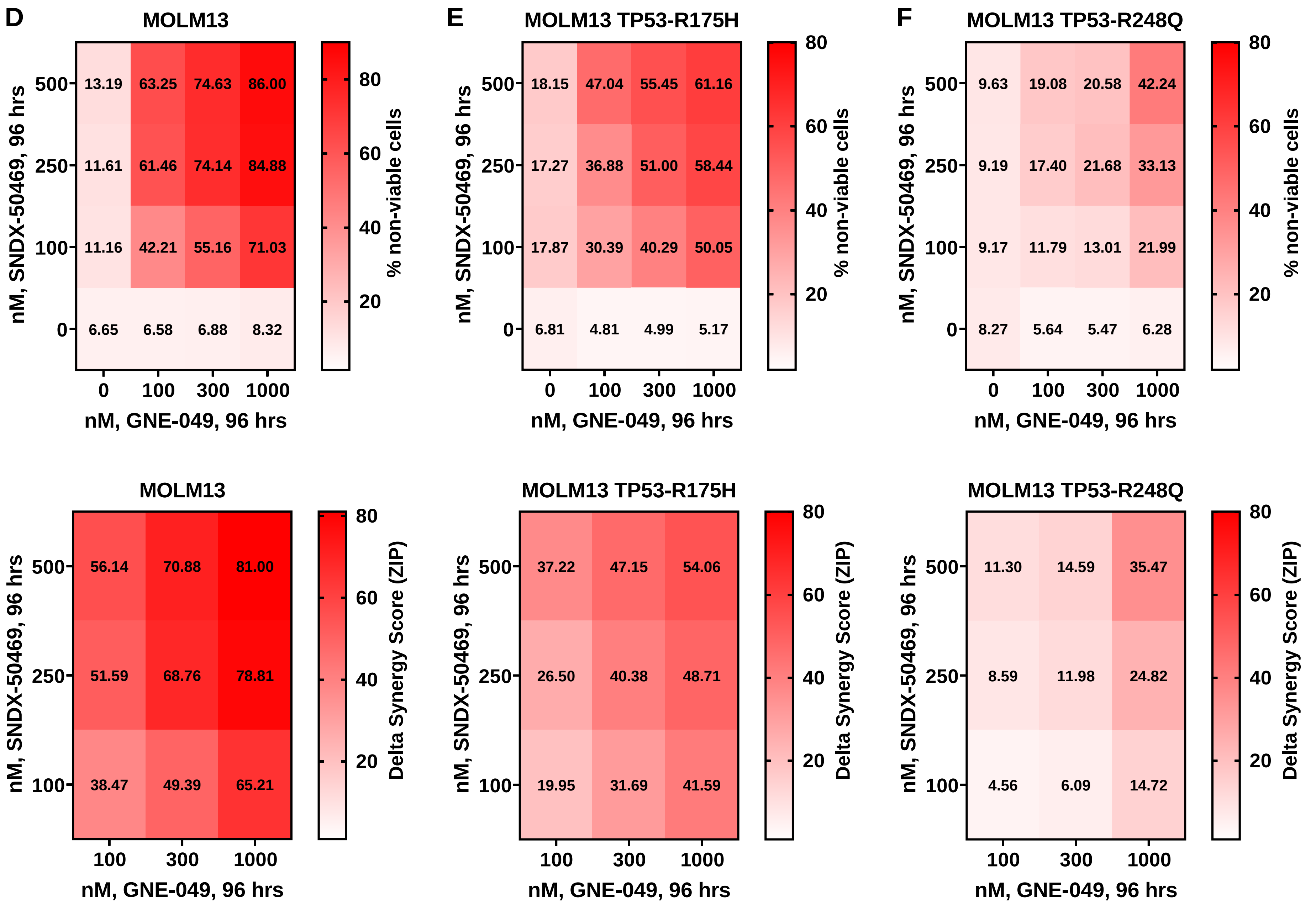
D
MOLM13
nM, SNDX-50469, 96 hrs
500
250
100
0
13.19 63.25 74.63 86.00
11.61 61.46 74.14 84.88
11.16 42.21 55.16 71.03
6.65 6.58 6.88 8.32
0 100 300 1000
nM, GNE-049, 96 hrs
% non-viable cells
80 60 40 20
E
MOLM13 TP53-R175H
nM, SNDX-50469, 96 hrs
500
250
100
0
18.15 47.04 55.45 61.16
17.27 36.88 51.00 58.44
17.87 30.39 40.29 50.05
6.81 4.81 4.99 5.17
0 100 300 1000
nM, GNE-049, 96 hrs
% non-viable cells
80 60 40 20
F
MOLM13 TP53-R248Q
nM, SNDX-50469, 96 hrs
500
250
100
0
9.63 19.08 20.58 42.24
9.19 17.40 21.68 33.13
9.17 11.79 13.01 21.99
8.27 5.64 5.47 6.28
0 100 300 1000
nM, GNE-049, 96 hrs
% non-viable cells
80 60 40 20
MOLM13
nM, SNDX-50469, 96 hrs
500
250
100
56.14 70.88 81.00
51.59 68.76 78.81
38.47 49.39 65.21
100 300 1000
nM, GNE-049, 96 hrs
Delta Synergy Score (ZIP)
80 60 40 20
MOLM13 TP53-R175H
nM, SNDX-50469, 96 hrs
500
250
100
37.22 47.15 54.06
26.50 40.38 48.71
19.95 31.69 41.59
100 300 1000
nM, GNE-049, 96 hrs
Delta Synergy Score (ZIP)
80 60 40 20
MOLM13 TP53-R248Q
nM, SNDX-50469, 96 hrs
500
250
100
11.30 14.59 35.47
8.59 11.98 24.82
4.56 6.09 14.72
100 300 1000
nM, GNE-049, 96 hrs
Delta Synergy Score (ZIP)
80 60 40 20

**Figure S11**

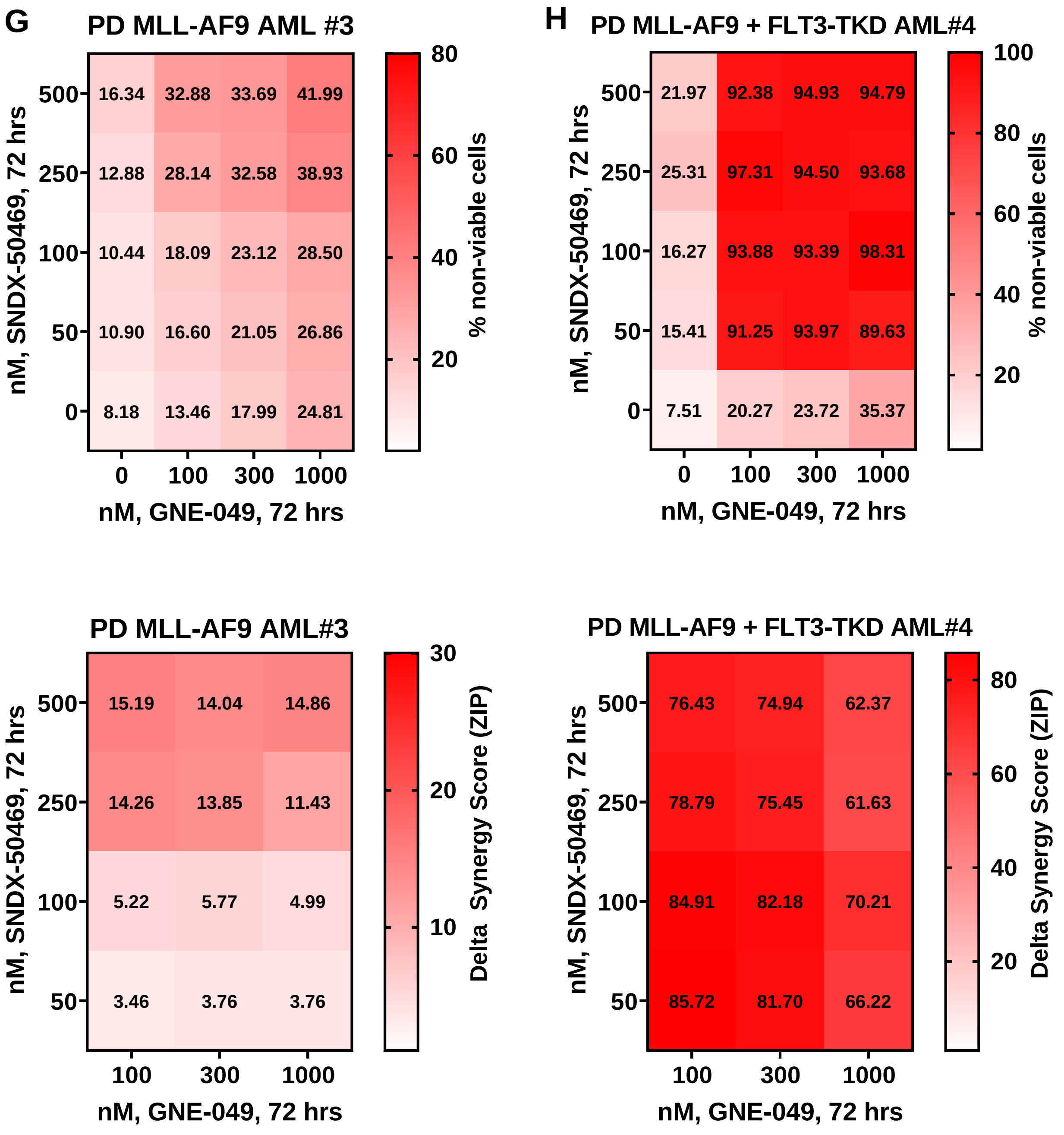
G
PD MLL-AF9 AML #3
nM, SNDX-50469, 72 hrs
500 250 100 50 0
16.34 32.88 33.69 41.99
12.88 28.14 32.58 38.93
10.44 18.09 23.12 28.50
10.90 16.60 21.05 26.86
8.18 13.46 17.99 24.81
0 100 300 1000
nM, GNE-049, 72 hrs
% non-viable cells
80 60 40 20
H
PD MLL-AF9 + FLT3-TKD AML#4
nM, SNDX-50469, 72 hrs
500 250 100 50 0
21.97 92.38 94.93 94.79
25.31 97.31 94.50 93.68
16.27 93.88 93.39 98.31
15.41 91.25 93.97 89.63
7.51 20.27 23.72 35.37
0 100 300 1000
nM, GNE-049, 72 hrs
% non-viable cells
100 80 60 40 20
PD MLL-AF9 AML#3
nM, SNDX-50469, 72 hrs
500 250 100 50
15.19 14.04 14.86
14.26 13.85 11.43
5.22 5.77 4.99
3.46 3.76 3.76
100 300 1000
nM, GNE-049, 72 hrs
Delta Synergy Score (ZIP)
30 20 10
PD MLL-AF9 + FLT3-TKD AML#4
nM, SNDX-50469, 72 hrs
500 250 100 50
76.43 74.94 62.37
78.79 75.45 61.63
84.91 82.18 70.21
85.72 81.70 66.22
100 300 1000
nM, GNE-049, 72 hrs
Delta Synergy Score (ZIP)
80 60 40 20

Figure S11

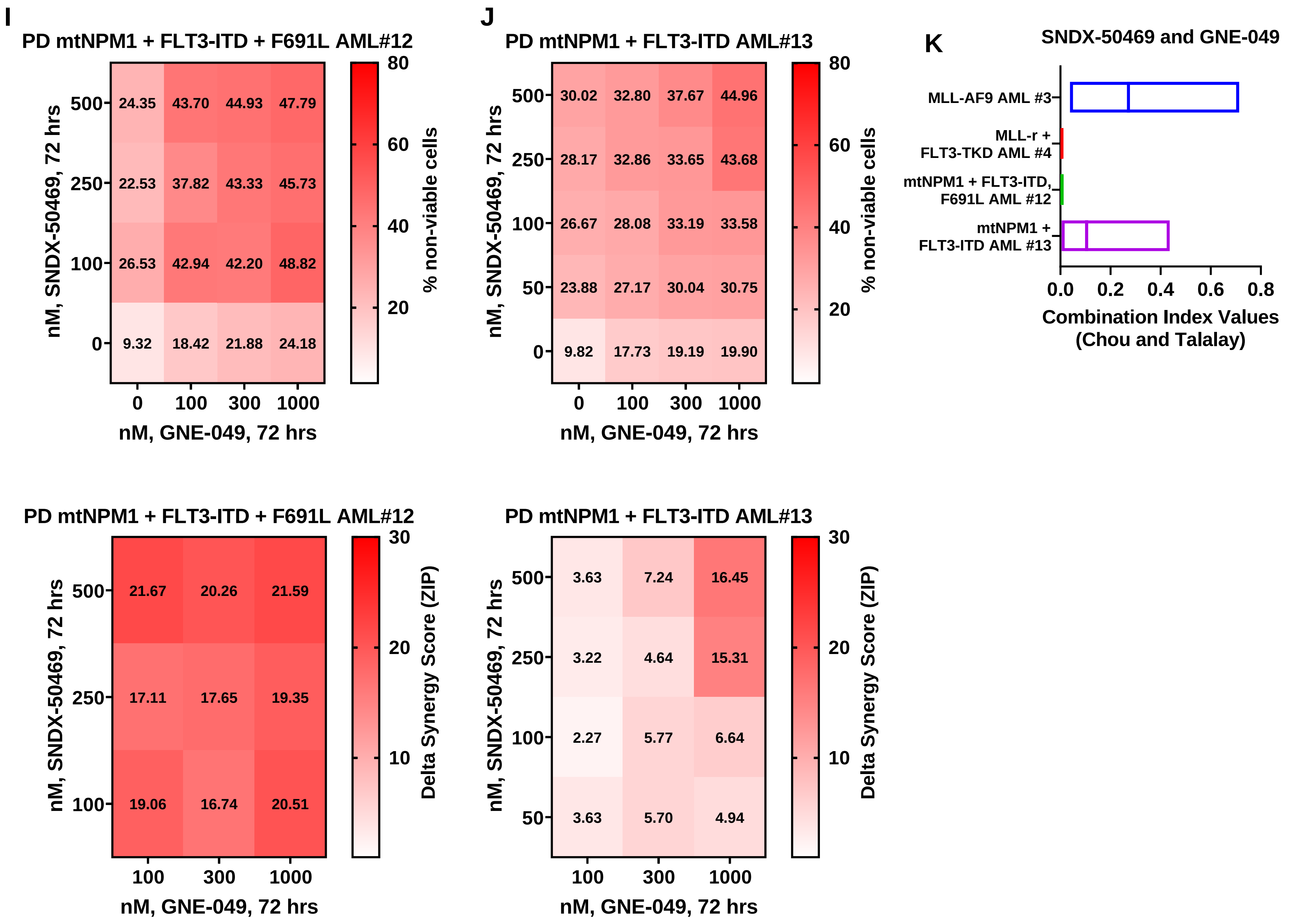
I
PD mtNPM1 + FLT3-ITD + F691L AML#12
nM, SNDX-50469, 72 hrs
500
250
100
0
24.35 43.70 44.93 47.79
22.53 37.82 43.33 45.73
26.53 42.94 42.20 48.82
9.32 18.42 21.88 24.18
0 100 300 1000
nM, GNE-049, 72 hrs
% non-viable cells
80 60 40 20
J
PD mtNPM1 + FLT3-ITD AML#13
nM, SNDX-50469, 72 hrs
500
250
100
50
0
30.02 32.80 37.67 44.96
28.17 32.86 33.65 43.68
26.67 28.08 33.19 33.58
23.88 27.17 30.04 30.75
9.82 17.73 19.19 19.90
0 100 300 1000
nM, GNE-049, 72 hrs
% non-viable cells
80 60 40 20
K
SNDX-50469 and GNE-049
MLL-AF9 AML #3
MLL-r + FLT3-TKD AML #4
mtNPM1 + FLT3-ITD, F691L AML #12
mtNPM1 + FLT3-ITD AML #13
0.0 0.2 0.4 0.6 0.8
Combination Index Values (Chou and Talalay)
PD mtNPM1 + FLT3-ITD + F691L AML#12
nM, SNDX-50469, 72 hrs
500
250
100
21.67 20.26 21.59
17.11 17.65 19.35
19.06 16.74 20.51
100 300 1000
nM, GNE-049, 72 hrs
Delta Synergy Score (ZIP)
30 20 10
PD mtNPM1 + FLT3-ITD AML#13
nM, SNDX-50469, 72 hrs
500
250
100
50
3.63 7.24 16.45
3.22 4.64 15.31
2.27 5.77 6.64
3.63 5.70 4.94
100 300 1000
nM, GNE-049, 72 hrs
Delta Synergy Score (ZIP)
30 20 10

Figure S11

L

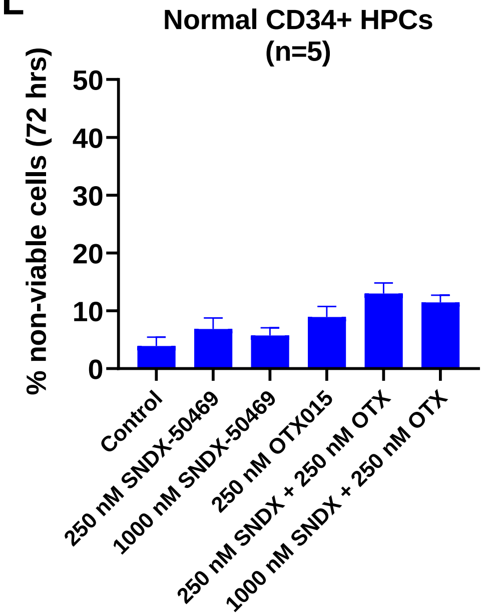


M

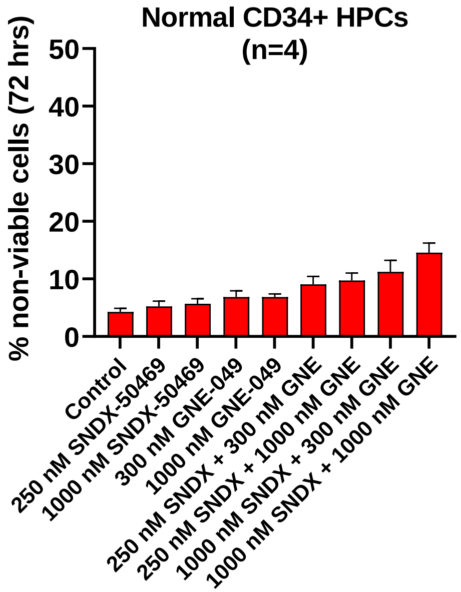


Figure S11

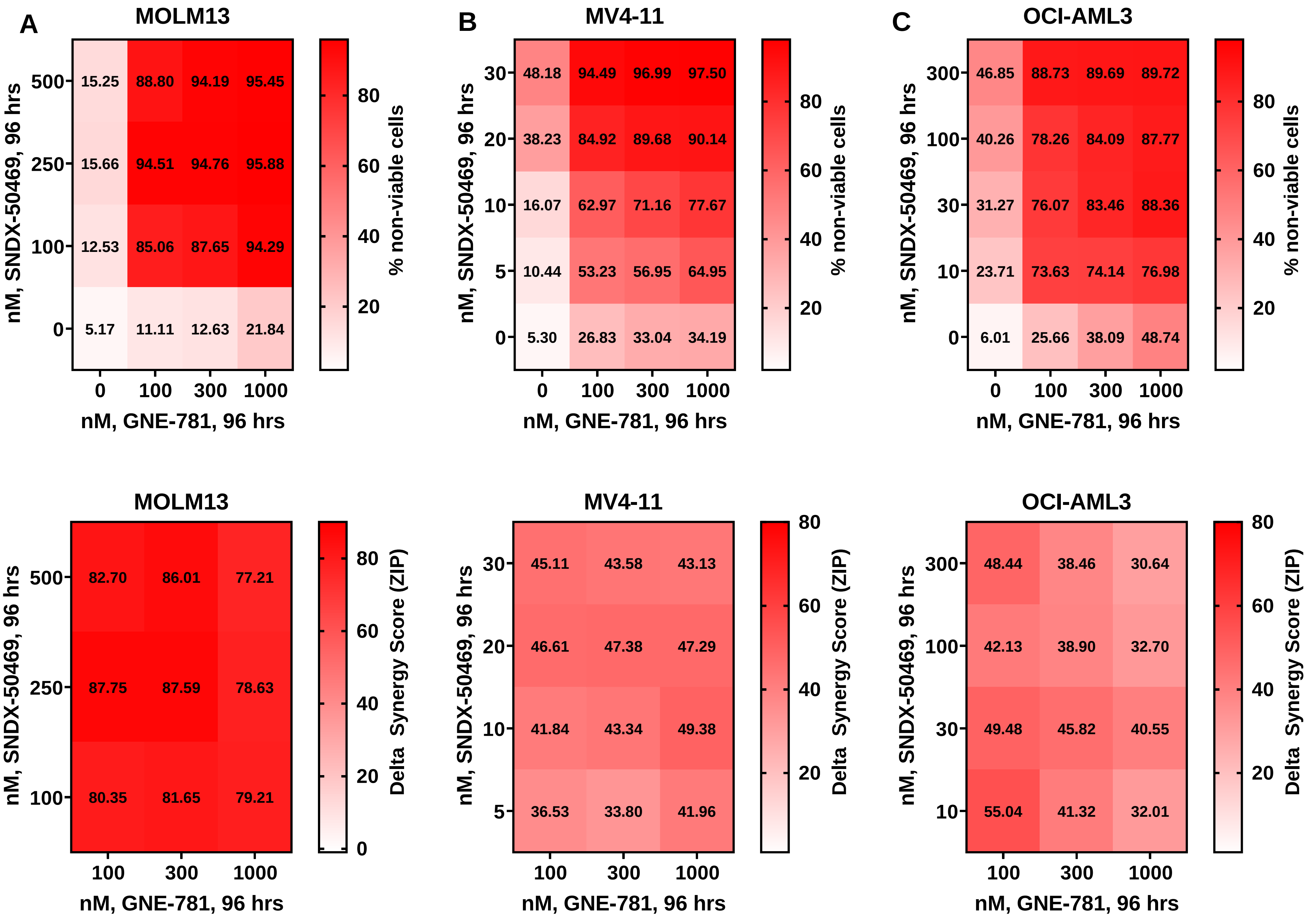
A
MOLM13
B
MV4-11
C
OCI-AML3
nM, SNDX-50469, 96 hrs
nM, GNE-781, 96 hrs
% non-viable cells
Delta Synergy Score (ZIP)
15.25 88.80 94.19 95.45
15.66 94.51 94.76 95.88
12.53 85.06 87.65 94.29
5.17 11.11 12.63 21.84
48.18 94.49 96.99 97.50
38.23 84.92 89.68 90.14
16.07 62.97 71.16 77.67
10.44 53.23 56.95 64.95
5.30 26.83 33.04 34.19
46.85 88.73 89.69 89.72
40.26 78.26 84.09 87.77
31.27 76.07 83.46 88.36
23.71 73.63 74.14 76.98
6.01 25.66 38.09 48.74
82.70 86.01 77.21
87.75 87.59 78.63
80.35 81.65 79.21
45.11 43.58 43.13
46.61 47.38 47.29
41.84 43.34 49.38
36.53 33.80 41.96
48.44 38.46 30.64
42.13 38.90 32.70
49.48 45.82 40.55
55.04 41.32 32.01

Figure S12

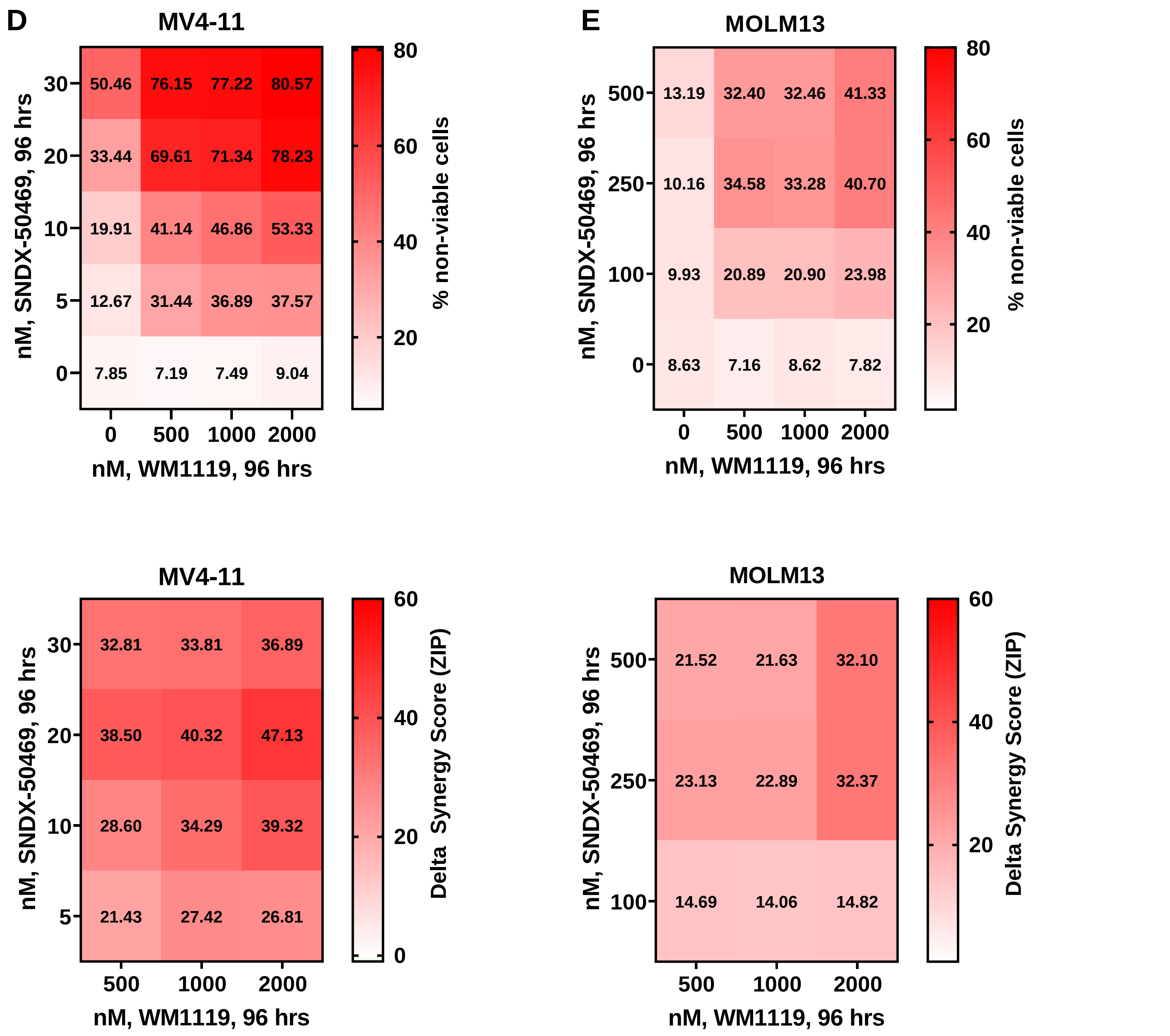
D
MV4-11
nM, SNDX-50469, 96 hrs
30
20
10
5
0
50.46 76.15 77.22 80.57
33.44 69.61 71.34 78.23
19.91 41.14 46.86 53.33
12.67 31.44 36.89 37.57
7.85 7.19 7.49 9.04
0 500 1000 2000
nM, WM1119, 96 hrs
% non-viable cells
80 60 40 20
E
MOLM13
nM, SNDX-50469, 96 hrs
500
250
100
0
13.19 32.40 32.46 41.33
10.16 34.58 33.28 40.70
9.93 20.89 20.90 23.98
8.63 7.16 8.62 7.82
0 500 1000 2000
nM, WM1119, 96 hrs
% non-viable cells
80 60 40 20
MV4-11
nM, SNDX-50469, 96 hrs
30
20
10
5
32.81 33.81 36.89
38.50 40.32 47.13
28.60 34.29 39.32
21.43 27.42 26.81
500 1000 2000
nM, WM1119, 96 hrs
Delta Synergy Score (ZIP)
60 40 20 0
MOLM13
nM, SNDX-50469, 96 hrs
500
250
100
21.52 21.63 32.10
23.13 22.89 32.37
14.69 14.06 14.82
500 1000 2000
nM, WM1119, 96 hrs
Delta Synergy Score (ZIP)
60 40 20

Figure S12

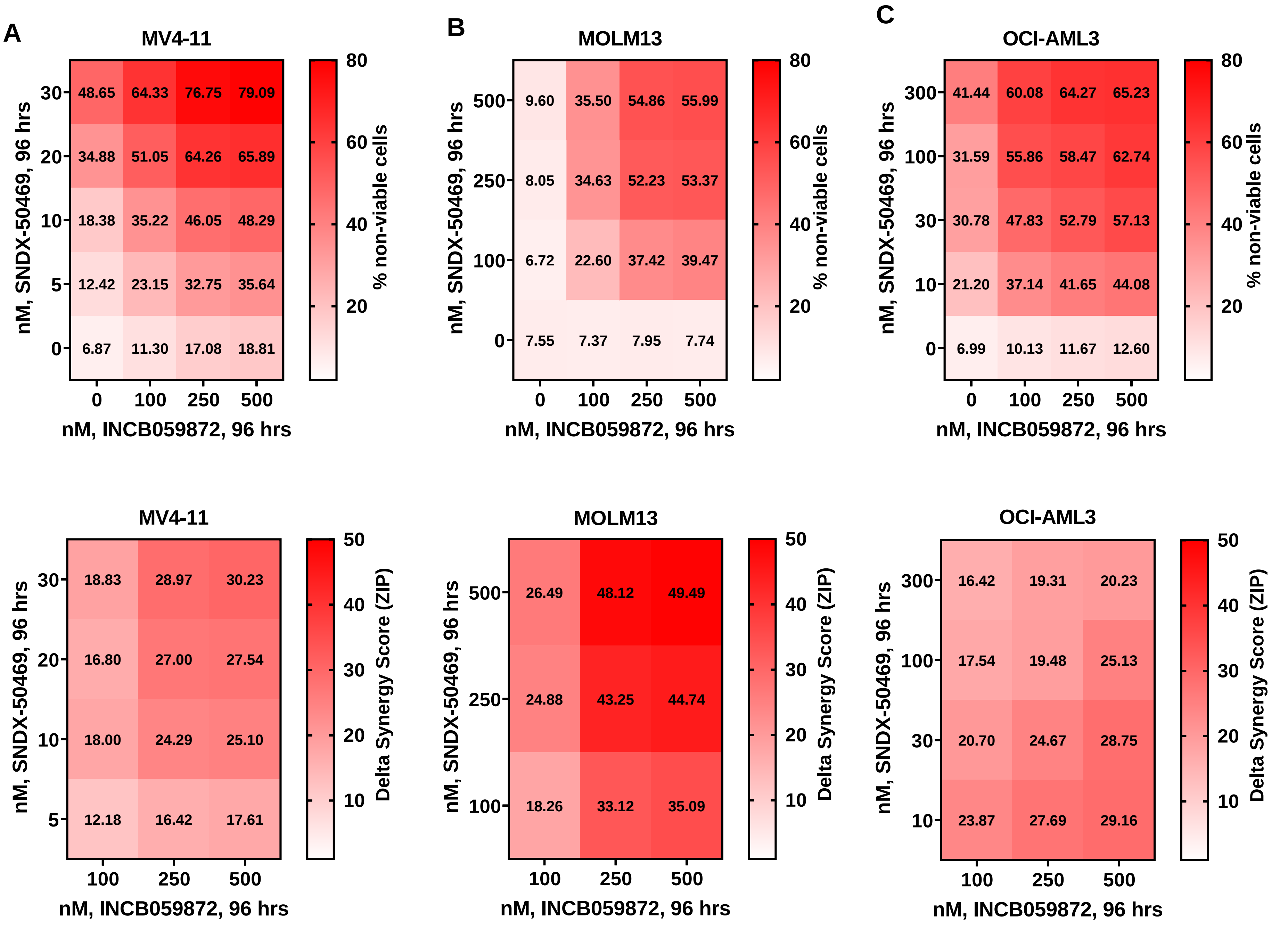
A
MV4-11
nM, SNDX-50469, 96 hrs
30 20 10 5 0
48.65 64.33 76.75 79.09
34.88 51.05 64.26 65.89
18.38 35.22 46.05 48.29
12.42 23.15 32.75 35.64
6.87 11.30 17.08 18.81
0 100 250 500
nM, INCB059872, 96 hrs
% non-viable cells
80 60 40 20
B
MOLM13
500 250 100 0
9.60 35.50 54.86 55.99
8.05 34.63 52.23 53.37
6.72 22.60 37.42 39.47
7.55 7.37 7.95 7.74
C
OCI-AML3
300 100 30 10 0
41.44 60.08 64.27 65.23
31.59 55.86 58.47 62.74
30.78 47.83 52.79 57.13
21.20 37.14 41.65 44.08
6.99 10.13 11.67 12.60
MV4-11
18.83 28.97 30.23
16.80 27.00 27.54
18.00 24.29 25.10
12.18 16.42 17.61
100 250 500
Delta Synergy Score (ZIP)
50 40 30 20 10
MOLM13
26.49 48.12 49.49
24.88 43.25 44.74
18.26 33.12 35.09
OCI-AML3
16.42 19.31 20.23
17.54 19.48 25.13
20.70 24.67 28.75
23.87 27.69 29.16

Figure S13

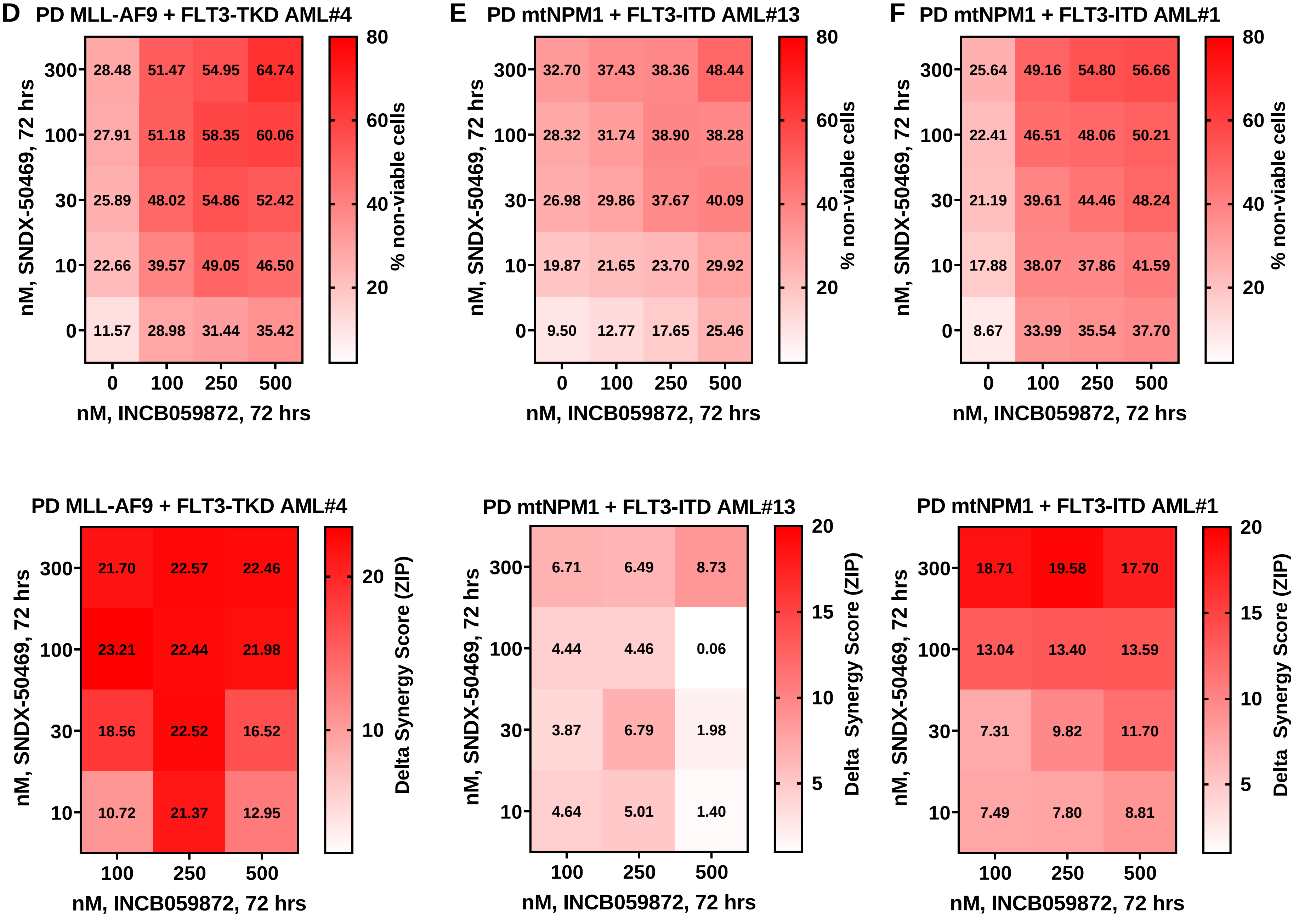
D
PD MLL-AF9 + FLT3-TKD AML#4
28.48 51.47 54.95 64.74
27.91 51.18 58.35 60.06
25.89 48.02 54.86 52.42
22.66 39.57 49.05 46.50
11.57 28.98 31.44 35.42
300 100 30 10 0
nM, SNDX-50469, 72 hrs
0 100 250 500
nM, INCB059872, 72 hrs
80 60 40 20
% non-viable cells
E
PD mtNPM1 + FLT3-ITD AML#13
32.70 37.43 38.36 48.44
28.32 31.74 38.90 38.28
26.98 29.86 37.67 40.09
19.87 21.65 23.70 29.92
9.50 12.77 17.65 25.46
300 100 30 10 0
nM, SNDX-50469, 72 hrs
0 100 250 500
nM, INCB059872, 72 hrs
80 60 40 20
% non-viable cells
F
PD mtNPM1 + FLT3-ITD AML#1
25.64 49.16 54.80 56.66
22.41 46.51 48.06 50.21
21.19 39.61 44.46 48.24
17.88 38.07 37.86 41.59
8.67 33.99 35.54 37.70
300 100 30 10 0
nM, SNDX-50469, 72 hrs
0 100 250 500
nM, INCB059872, 72 hrs
80 60 40 20
% non-viable cells
PD MLL-AF9 + FLT3-TKD AML#4
21.70 22.57 22.46
23.21 22.44 21.98
18.56 22.52 16.52
10.72 21.37 12.95
300 100 30 10
nM, SNDX-50469, 72 hrs
100 250 500
nM, INCB059872, 72 hrs
20 10
Delta Synergy Score (ZIP)
PD mtNPM1 + FLT3-ITD AML#13
6.71 6.49 8.73
4.44 4.46 0.06
3.87 6.79 1.98
4.64 5.01 1.40
300 100 30 10
nM, SNDX-50469, 72 hrs
100 250 500
nM, INCB059872, 72 hrs
20 15 10 5
Delta Synergy Score (ZIP)
PD mtNPM1 + FLT3-ITD AML#1
18.71 19.58 17.70
13.04 13.40 13.59
7.31 9.82 11.70
7.49 7.80 8.81
300 100 30 10
nM, SNDX-50469, 72 hrs
100 250 500
nM, INCB059872, 72 hrs
20 15 10 5
Delta Synergy Score (ZIP)

**Figure S13**

G

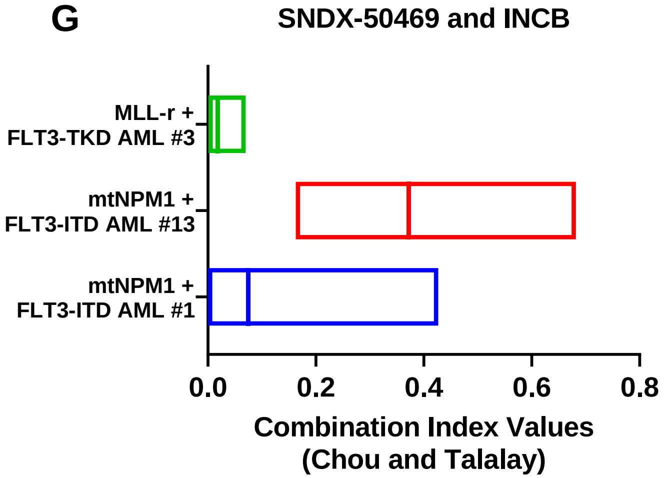
SNDX-50469 and INCB
MLL-r +
FLT3-TKD AML #3
mtNPM1 +
FLT3-ITD AML #13
mtNPM1 +
FLT3-ITD AML #1
0.0
0.2
0.4
0.6
0.8
Combination Index Values
(Chou and Talalay)

Figure S13

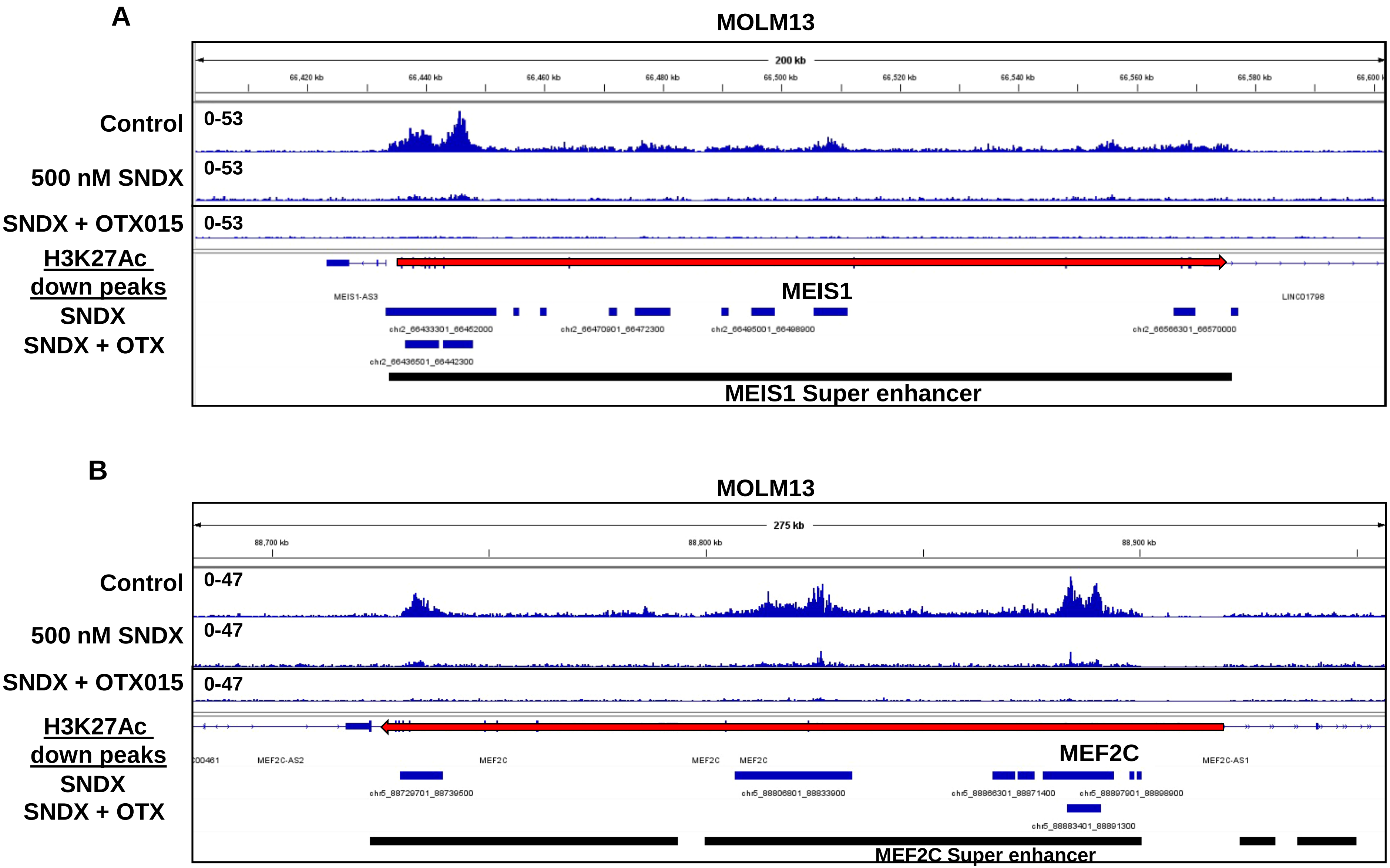
A
MOLM13
200 kb
66,420 kb
66,440 kb
66,460 kb
66,480 kb
66,500 kb
66,520 kb
66,540 kb
66,560 kb
66,580 kb
Control
0-53
500 nM SNDX
0-53
SNDX + OTX015
0-53
H3K27Ac down peaks
SNDX
SNDX + OTX
MEIS1-AS3
MEIS1
LINC01798
chr2_66433301_66452000
chr2_66470901_66472300
chr2_66495001_66498900
chr2_66566301_66570000
chr2_66436501_66442300
MEIS1 Super enhancer
B
MOLM13
275 kb
88,700 kb
88,800 kb
88,900 kb
Control
0-47
500 nM SNDX
0-47
SNDX + OTX015
0-47
H3K27Ac down peaks
SNDX
SNDX + OTX
MEF2C-AS2
MEF2C
MEF2C
MEF2C
MEF2C
MEF2C-AS1
chr5_88729701_88739500
chr5_88806801_88833900
chr5_88866301_88871400
chr5_88897901_88898900
chr5_88883401_88891300
MEF2C Super enhancer

Figure S14

C

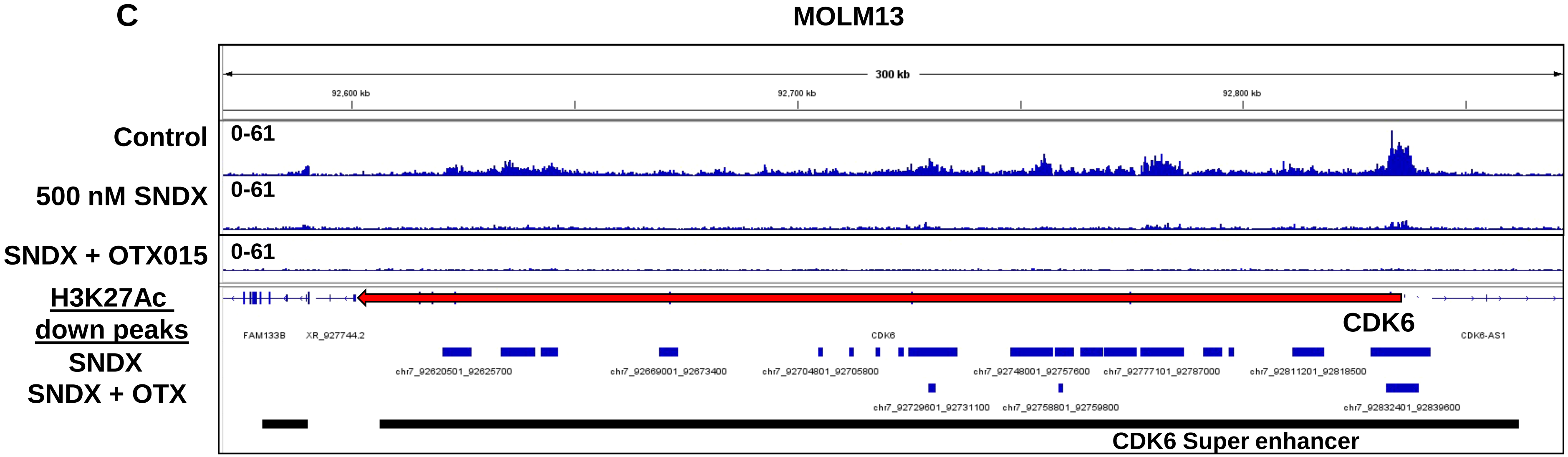


D

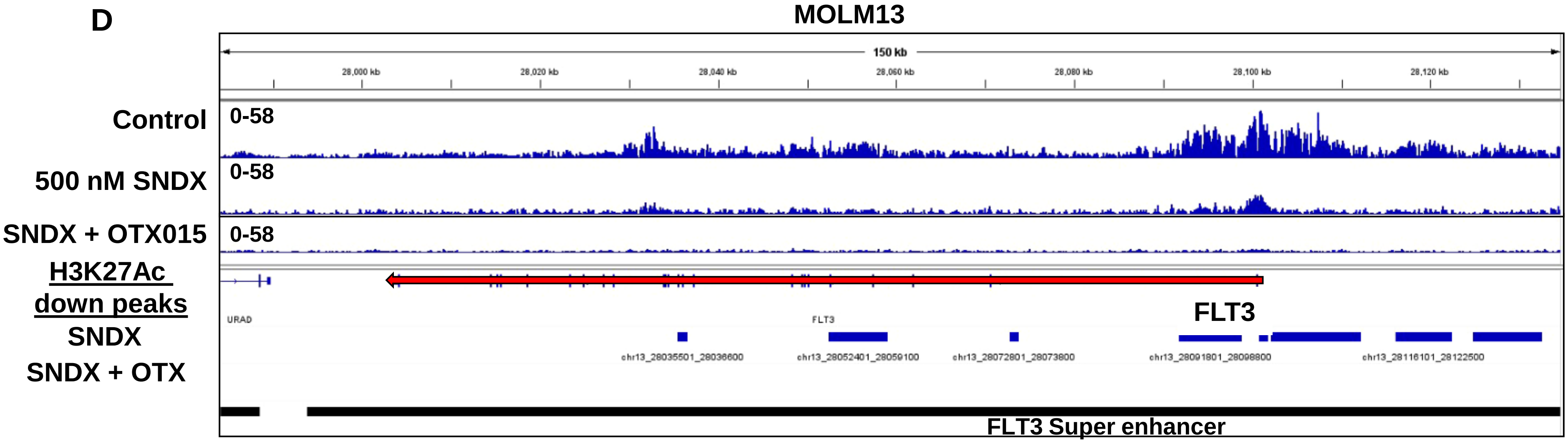


Figure S14

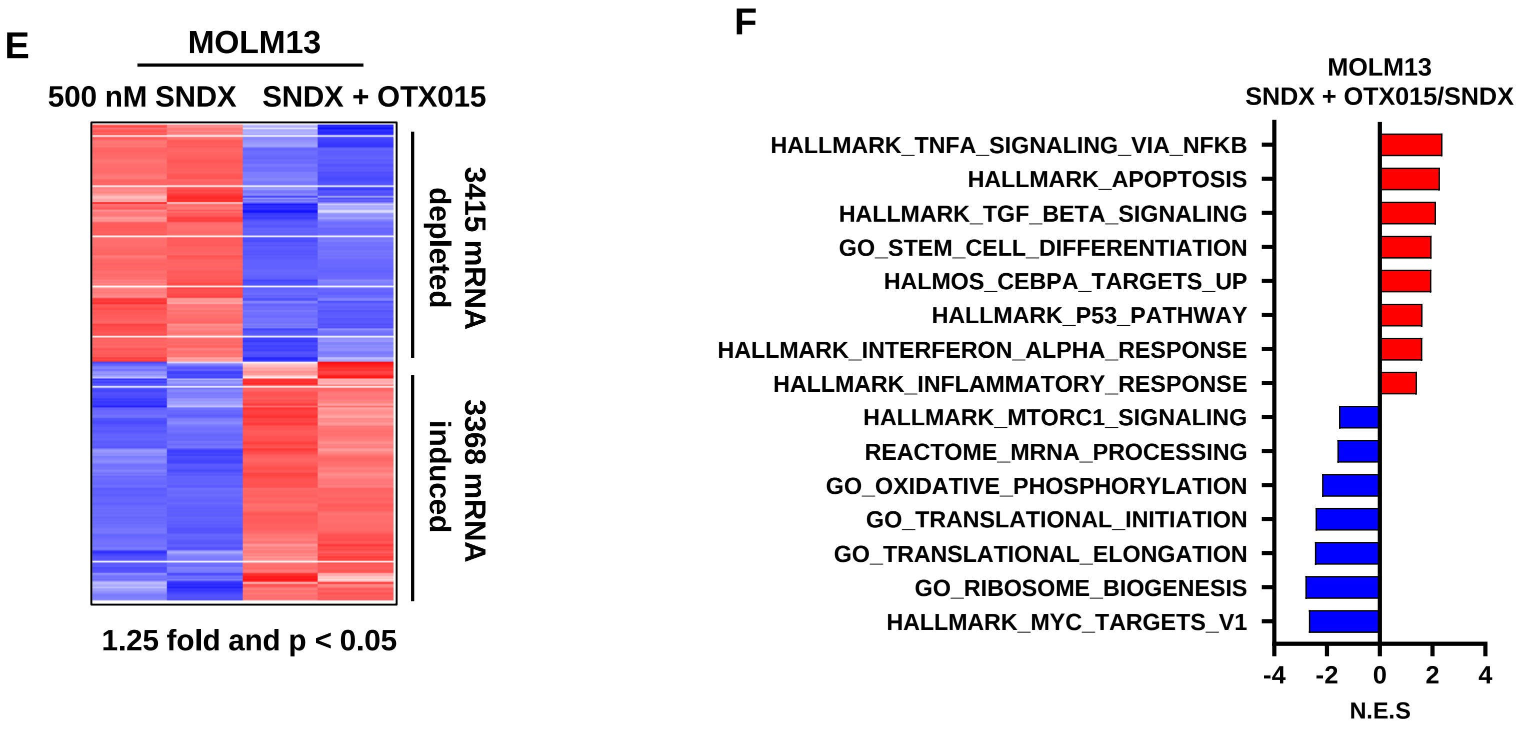
E
MOLM13
500 nM SNDX
SNDX + OTX015
3415 mRNA depleted
3368 mRNA induced
1.25 fold and p < 0.05
F
MOLM13
SNDX + OTX015/SNDX
HALLMARK_TNFA_SIGNALING_VIA_NFKB
HALLMARK_APOPTOSIS
HALLMARK_TGF_BETA_SIGNALING
GO_STEM_CELL_DIFFERENTIATION
HALMOS_CEBPA_TARGETS_UP
HALLMARK_P53_PATHWAY
HALLMARK_INTERFERON_ALPHA_RESPONSE
HALLMARK_INFLAMMATORY_RESPONSE
HALLMARK_MTORC1_SIGNALING
REACTOME_MRNA_PROCESSING
GO_OXIDATIVE_PHOSPHORYLATION
GO_TRANSLATIONAL_INITIATION
GO_TRANSLATIONAL_ELONGATION
GO_RIBOSOME_BIOGENESIS
HALLMARK_MYC_TARGETS_V1
-4
-2
0
2
4
N.E.S

**Figure S14**
