# Supplementary material for: Targeting of epigenetic co-dependencies enhances anti-AML efficacy of Menin inhibitor in AML with MLL1-r or mutant NPM1
Source: Blood Cancer J. 2023 Apr 13;13(1):53. doi: 10.1038/s41408-023-00826-6 (PMC10102188; doi:10.1038/s41408-023-00826-6)
Supplement: Supplementary file 4 — Supplemental Materials and Methods [file 41408_2023_826_MOESM4_ESM.docx]

**Supplemental Methods:**

**Contact for Reagent sharing. Kapil N Bhalla. Department of Leukemia, MD. Anderson Cancer Center, 1400 Holcombe Blvd, Unit428, Houston, TX, 77030. kbhalla@mdanderson.org**

**Reagents and antibodies.** SNDX-50469, GNE-049, GNE-781, INCB059872 and WM1119 for in vitro studies were obtained from MedChem Express (Monmouth Junction, NJ). DTAG-13 (Cat. No. 6605) was obtained from Tocris/Bio-Techne (Minneapolis, MN). All compounds were prepared as 10 mM stocks in 100% DMSO and frozen at -80°C in 5-10 µL aliquots to allow for single use, thus avoiding multiple freeze-thaw cycles that could result in compound decomposition and loss of activity. SNDX-5613 for in vivo studies was obtained from Syndax Pharmaceuticals Inc. (Waltham, MA) under a material transfer agreement. GNE-781 for in vivo studies was synthesized by the Institute for Applied Cancer Science (IACS) labs at the M.D. Anderson Cancer Center (Houston, TX). Anti-c-Myc [RRID:AB_1903938], anti-Menin [RRID:AB_10858216], anti-MEF2C [RRID:AB_10548759], anti-p21 Waf1/Cip1 [RRID:AB_823586] and anti-C/EBPα [RRID:AB_11178517] antibodies were obtained from Cell Signaling Technologies (Beverly, MA). Anti-HA-tag [RRID:AB_444303], Anti-MEIS1 [RRID:AB_776272], anti-FLT3 [Cat# ab245116], anti-PBX3 [RRID:AB_10858991], and anti-CD11b [RRID:AB_2650514] antibodies were obtained from Abcam (Cambridge, MA). Anti-HOXA9 [Cat# 07-178; RRID:AB_310414] antibody was obtained from Millipore-Sigma (Burlington, MA). Anti-CDK6 [RRID:AB_10610066], anti-BCL2 [RRID:AB_626733], anti-GAPDH [RRID:AB_627679] and anti-β-Actin [RRID:AB_626630] antibodies were obtained from Santa Cruz Biotechnologies (Santa Cruz, CA). Anti-p27 [RRID:AB_397636] antibody was obtained from BD Transduction Labs (Franklin Lakes, NJ). Anti-BRD4 antibody (Catalog # A301-985A-M) was obtained from Fortis Life Sciences (Waltham, MA).

**Cell lines and cell culture.** MOLM13 [DSMZ Cat# ACC-554, RRID: CVCL_2119], OCI-AML2 [DSMZ Cat# ACC-99, RRID:CVCL_1619) and OCI-AML3 [DSMZ Cat# ACC-582, RRID:CVCL_1844] cells were obtained from the DSMZ (Braunschweig, Germany). MV4-11 [ATCC Cat# CRL-9591, RRID:CVCL_0064] and THP-1 [ATCC Cat# TIB-202; RRID:CVCL_0006] cells were obtained from the ATCC (Manassas, VA). MOLM13 cells with isogenic TP53 mutations [R175H, R248Q and TP53-KO] were a gift from Dr. Benjamin L. Ebert (Dana Farber Cancer Center, Boston, MA). HEK-293T [RRID:CVCL_0063] cells were obtained from the Characterized Cell Line Core Facility at M.D. Anderson Cancer Center, Houston TX. All experiments with cell lines were performed within 6 months after thawing or obtaining from ATCC or DSMZ. MOLM13 and OCI-AML3 cells were cultured in RPMI-1640 media with 20% FBS, 1% penicillin/streptomycin and 1% non-essential amino acids. OCI-AML2 cells were cultured in MEM-alpha media with 20% FBS 1% penicillin/streptomycin and 1% non-essential amino acids. MV4-11 cells were cultured in ATCC-formulated IMDM media with 20% FBS, 1% penicillin/streptomycin and 1% non-essential amino acids. HEK-293T cells were cultured in high-glucose-formulated DMEM media with 10% FBS, 1% penicillin/streptomycin and 1% glutamine. Logarithmically growing, mycoplasma-negative cells were utilized for all experiments. Following drug treatments, cells were washed free of the drug(s) prior to the performance of the studies described.

**Cell Line Authentication**. The cell lines utilized in these studies were authenticated in the Characterized Cell Line Core Facility at M.D. Anderson Cancer Center, Houston TX utilizing STR profiling.

**Plasmid Generation, Viral Packaging, and Creation of Cell Lines.** Plasmid constructs for the production of lentivirus were transfected with packaging plasmids psPAX2 and pMD2.G into HEK-293T cells utilizing jetPRIME reagent (PolyPlus Transfection, New York, NY). The psPAX2 and pMD2.G packaging plasmids were a gift from Didier Trono (Addgene plasmid #12260 and #12259 [RRID: Addgene_12260; RRID: Addgene_12259]). Media was changed the following day. Viral supernatant was collected 72 hours post transfection and filtered through a 0.45 µm PES membrane. AML cells were seeded at 5 x 10^5^ cells/mL in a 50:50 mix of media and lentiviral supernatant with 8 µg/mL polybrene (Sigma-Aldrich, Burlington, MA). The following day, the viral supernatant was removed by centrifugation and cells were transduced with fresh viral supernatant for an additional 24 hours. To generate luciferase-expressing AML cells, pHIV-Luc-ZsGreen (a gift from Bryan Welm [Addgene plasmid #39196; http://n2t.net/addgene:39196; RRID:Addgene_39196]) was packaged as above and transduced into MOLM13 or patient-derived AML PDX cells. ZsGreen-positive cells were sorted by flow cytometry (FACSAria, FL-1 channel, top 10% brightest GFP-expressing cells), and expanded in culture or grown in NSG mice prior to their utilization in therapeutic in vivo mouse studies. To generate AML cells, MOLM13 and MV4-11 with stable expression of SP-Cas9, lentiCas9-Blast (a kind gift from Feng Zhang; Addgene #52962; RRID:Addgene_52962) was utilized. Transduced cells were selected with 8-10 µg/mL of blasticidin for 10 days. Expression of Cas9 in AML cells was confirmed by immunoblot analyses utilizing anti-FLAG M2 [Sigma-Aldrich Cat# F3165, RRID:AB_259529] and anti-Cas9 [Cat# 698302, RRID:AB_2715782] antibodies (BioLegend, San Diego, CA).

**CRISPR/Cas9-mediated gene editing in cultured AML cells.** To study the effects of knockout/depletion of Menin in AML cells, the CHOP-CHOP prediction algorithm (1) was utilized to develop guide RNAs. Guide RNAs were developed against exon 2 and exon 6 sequence of Menin. High scoring sgRNAs were synthesized by Synthego, Inc. Negative control sgRNA was also obtained from Synthego, Inc. For MOLM13 cells without stable Cas9 expression, to obtain Cas9-sgRNA RNPs (ribonucleoprotein complexes), 1 μg of synthetic sgRNA was incubated with 1.5 μg recombinant Cas9 protein (Synthego, Inc.) for 15 min at room temperature. MOLM13 cells were transfected by electroporation utilizing the Amaxa 2D-Nucleofector device with Nucleofector Kit-C and program X-001 (Amaxa GmbH, Cologne, Germany) as per the manufacturer's instructions. Immediately post-transfection, cells were plated in complete media containing no antibiotics and 2 µM of ROCK inhibitor (Y-27632, Selleck Chemicals; Houston, TX) and allowed to recover for 24 hours. Knockout of Menin was confirmed by mRNA expression (qPCR) and Western blot analysis 5-6 days post-transfection. Gene-edited AML cells were treated with OTX015 for 48 hours or INCB059872 for 96 hours and the % of Annexin V-positive, apoptotic cells or TO-PRO-3iodide positive, non-viable cells were determined by flow cytometry. For knockout of BRD4, two pre-designed TrueGuide™ Synthetic sgRNAs targeting exon 5 or exon 6 were obtained from Life Technologies (Carlsbad, CA) and transfected as above. Knockout of BRD4 was confirmed by immunoblot analysis 5 days post-transfection. For treatment with Menin inhibitor, MOLM13 cells were transfected with negative control sgRNA or BRD4 sgRNAs and incubated for 72 hours. Then, cells were washed with complete RPMI media, plated at 125 x 10^5^ cells/mL and treated with SNDX-50469 for 96 hours. The TO-PRO-3 iodide positive, non-viable cells were determined by flow cytometry.

**Generation of a dTAG13-responsive Menin-FKBP12^F36V^-HA expressing AML cell line.** To generate an in-frame fusion between the Menin cDNA and the F36V mutant FKBP12 cDNA, a Menin cDNA clone (Clone ID:100003387; Catalog ID: OHS5893-202491930) was obtained from Dharmacon/Horizon Discovery (Boulder, CO). The mutant FKBP12 cDNA was amplified from pET15b His6-FKBPF36V which was a gift from Thomas Wandless (Addgene plasmid #73180) [RRID:Addgene_73180]. An attB1 site was incorporated into the N-terminus of the primer for Menin (attB1 Menin.for 5’- GCTT **ACA AGT TTG TAC AAA AAA GCA GGC TTC** ACC ATG GGG GCT GAA GGC CGC-3’) and a *Not1* restriction site into the reverse primer (NotI Menin.rev 5’- GTC A **GCG GCC GC** GAG GCC TTT GCG CTG CC-3’), thus removing the native stop codon. A *Not1* restriction site was designed into the forward primer of the FKBP12 cDNA (NotI FKBP12.for 5’-GTC A **GCG GCC GC**T GGA GTG CAG GTG GAA ACC ATC TC-3’) and a C-terminal HA-tag and attB2 site were designed into the reverse primer (attB2 HA FKBP12.rev 5’-GGGG **AC CAC TTT GTA CAA GAA AGC TGG GTA** TCA AGC GTA ATC TGG AAC ATC GTA TGG GTA AGC GTA ATC TGG AAC ATC GTA TGG GTA TTC CAG TTT TAG AAG CTC CAC ATC GAA GAC-3’). The cDNAs were amplified by PCR using Phusion^TM^ polymerase (ThermoFisher Scientific, Waltham, MA) according to the manufacturer’s recommendations for temperature and extension times. PCR products were column-purified and digested with *Not1* overnight at 37°C. The resulting fragments were gel purified and ligated with T4 DNA ligase overnight at 16°C. The pDONR^TM^221 vector (Invitrogen, Carlsbad, CA) was utilized for the BP clonase reaction. The Menin-FKBP12 fusion DNA was introduced into the Gateway cloning vector pDONR^TM^221 following the manufacturer’s BP clonase protocol and incubating the reaction at room temperature for 2 hours. The recombined DNA was transformed into *E. coli* TOP10 cells and selected with 100 µg/mL of ampicillin. Transformants were checked by DNA sequencing. The fusion cDNA was transferred by Gateway cloning into pLEX_305, a kind gift from David Root (Addgene # 41390) [RRID:Addgene_41390] utilizing an LR clonase reaction. The resulting plasmid was transformed into *E. coli* DH5 alpha cells and selected with 100 µg/mL of ampicillin. Positive clones were confirmed by DNA sequencing and then pLEX_305/Menin-FKBP12^(F36V)^-HA vector was combined with packaging vectors (as above) to generate lentiviral particles in HEK293T cells. MOLM13 and MV4-11 cells that had been previously engineered to stably express SP-Cas9 (as above) were transduced with lentivirus supernatants and selected with 0.5 µg/mL of puromycin for 96 hours. Next, two splice-blocking sgRNAs were used to knock out the endogenous Menin: one in the intron between exon 3 and exon 4, and one in the intron between exon 5 and exon 6. DNA oligos were synthesized, annealed to each other in annealing buffer, then ligated into digested LRG [Lenti_sgRNA_EFS_GFP] vector, a kind gift from Christopher Vakoc (Addgene #65656) [RRID: Addgene_65656] and transformed into *E. coli* Sure2 cells (Part Number: 200152; Agilent Technologies, Santa Clara, CA). Positive transformants were confirmed by Sanger sequencing, then combined with packaging vectors to generate lentiviral particles. Lentiviral supernatant was combined with MOLM13-Menin-FKBP12^(F36V)^-HA expressing cells or MV4-11-Menin-FKBP12^(F36V)^-HA expressing cells and incubated for 48 hours. GFP-positive cells were selected by FACS sorting and expanded for experimentation. dTAG-13 was synthesized as previously described (2). Cells were treated with dTAG-13 as indicated.

**Primary AML blasts.** Patient-derived AML cells samples were obtained with informed consent as part of a clinical protocol approved by the Institutional Review Board of The University of Texas, M.D. Anderson Cancer Center. Mononuclear cells were purified by Ficoll Hypaque (Axis Shield, Oslo, Norway) density centrifugation following the manufacturer’s protocol. Mononuclear cells were washed once with sterile 1X PBS then suspended in complete RPMI media containing 20% FBS. Cells were counted to determine the number of cells isolated prior to immuno-magnetic selection. CD34+ AML blast progenitor cells were purified by immuno-magnetic beads conjugated with anti-CD34 antibody following the manufacturer’s protocol (StemCell Technologies, Vancouver, British Columbia) prior to utilization in the cell viability assays, RNA expression, and immunoblot analyses.

**Sequencing of primary de novo blast cells.** We performed targeted next-generation sequencing (NGS) of DNA samples from bone marrow or peripheral blood collected from patients at our center with de novo AML (3). Diagnostic bone marrow samples were obtained for mutational analysis. Total genomic DNA was extracted from unenriched peripheral blood (PB) or bone marrow (BM) samples using ReliaPrep genomic DNA isolation kit (Promega Corp, Madison, WI, USA). Briefly, a total of 250 ng DNA was utilized to prepare sequencing libraries using Agilent HaloPlex custom Kit (Agilent Technologies, Santa Clara, CA, USA). The entire coding sequences of 81 genes including ABL1, ASXL1, BRAF, CALR, DNMT3A, EGFR, EZH2, FLT3, GATA1, GATA2, HRAS, IDH1, IDH2, KIT, KRAS, MDM2, IKZF2, JAK1, JAK2, MLL, MPL, MYD88, NOTCH1, NF1, NPM1, NRAS, PTPN11, RUNX1, TET2, TP53, and WT1 were interrogated on a custom-designed next-generation sequencing approach using the Illumina MiSeq platform (Illumina; San Diego, CA, USA; RRID:SCR_016379). The genomic reference sequence used was genome GRch37/hg19. The following software tools were utilized in the experimental setup and data analysis: Illumina Experiment Manager 1.6.0 (Illumina; San Diego, CA, USA), MiSeq Control Software 2.4 (Illumina; San Diego, CA, USA), Real Time Analysis 1.18.54 (Illumina; San Diego, CA, USA), Sequence Analysis Viewer 1.8.37 (Illumina; San Diego, CA, USA), MiSeq Reporter 2.5.1 (Illumina; San Diego, CA, USA), and SureCall 3.0.1.4 (Agilent Technologies; Santa Clara, CA, USA). A minimum of 80% reads at quality scores of AQ30 or higher were required to pass quality control. The lower limit of detection of this assay (analytical sensitivity) for single nucleotide variations was determined to be 5% (one mutant allele in the background of nineteen wild type alleles) to 10% (one mutant allele in the background of nine wild type alleles). Testing of patients with active hematologic malignancies was limited to somatic mutations only.

**Analysis of epigenetic state in AML cells *in vitro***. ATAC-Seq analysis of untreated and treated AML cells was performed following a previously described protocol (4). ATAC-Seq libraries were generated with a Nextera DNA Library Preparation Kit containing the mutant Tn5 transposase (Illumina, San Diego, CA; Catalog number: FC-121-1030). The DNA fragments were indexed utilizing a Nextera Index Kit (Illumina, San Diego, CA; Catalog number: FC-121-1011) and amplified by PCR utilizing NEBNext® High-Fidelity 2X PCR Master Mix according to the manufacturer’s protocol (New England Biolabs, Ipswich, MA). Library fragments were amplified for 10-11 cycles utilizing the denaturation, annealing, and extension times as previously described (4). The amplified library fragments were PCR-purified with a Qiagen MinElute column (Qiagen, Germantown, MD) then size selected with a 1.0X bead concentration to remove fragments shorter than 200 bp. Library fragments were incubated with AMPure XP SPRI beads (Beckman Coulter, Indianapolis, IN) for 10 minutes at room temperature in 1.5 mL microcentrifuge tubes. The mixture was placed on a magnetic stand for 10 minutes. The supernatant was removed and the SPRI beads were washed twice with fresh 80% ethanol (30 seconds for each wash) and air-dried for 2-3 minutes. Library DNA was eluted from the SPRI beads with a 20 µL volume of 10 mM Tris-HCl (pH 8.5). Beads were incubated at room temperature for 10 minutes, then the tubes were transferred to a magnetic stand for 10 minutes. The supernatant containing the DNA libraries was carefully removed by pipetting and transferred into a clean microcentrifuge tube. The individual libraries were quantified and quality-checked by Thermo Fisher Qubit [Thermo Fisher Qubit fluorimeter, RRID:SCR_018095] fluorometric quantification and Agilent Bioanalyzer 2100 [Agilent 2100 Bioanalyzer Instrument, RRID:SCR_019389] analysis, respectively. Individual libraries were pooled into one tube, purified over a Qiagen MinElute column [QIAGEN, RRID:SCR_008539], eluted in 20 µL of 10 mM Tris, (pH 8.5) and sequenced on a NextSeq 500 next generation sequencer (Illumina NextSeq 500, RRID:SCR_014983) utilizing a 150 cycle mid-output kit (Illumina, San Diego, CA). Raw sequencing data was mapped using TopHat2 (5) [TopHat, RRID:SCR_013035] onto the human genome build UCSC hg38 (NCBI 51) for human data and log2-fold changes were calculated with diffReps (6) [diffReps, RRID:SCR_010873]. Sequence tracks were visualized with IGV software [Integrative Genomics Viewer, RRID:SCR_011793] (7,8). We also determined the H3K27Ac status and BRD4 occupancy in untreated and Menin inhibitor-treated MOLM13 cells by ChIPmentation following a previously described protocol (9), ChIP-Seq libraries were generated with a Nextera DNA Library Preparation Kit containing the mutant Tn5 transposase (Illumina, San Diego, CA; Catalog number: FC-121-1030). The DNA fragments were indexed utilizing a Nextera Index Kit (Illumina, San Diego, CA; Catalog number: FC-121-1011) and amplified by PCR utilizing NEBNext® High-Fidelity 2X PCR Master Mix according to the manufacturer’s protocol (New England Biolabs, Ipswich, MA). Library fragments were amplified for 12-15 cycles utilizing the denaturation, annealing, and extension times as previously described (9). The amplified library fragments were PCR-purified with a Qiagen MinElute column (Qiagen, Germantown, MD) then size selected with a 0.65X bead volume to remove large fragments (remaining on the beads), then the supernatant was combined with a 1.0X SPRI bead volume to remove fragments shorter than 200 bp. Library fragments were incubated with AMPure XP SPRI beads (Beckman Coulter, Indianapolis, IN) for 10 minutes at room temperature in 1.5 mL microcentrifuge tubes. The mixture was placed on a magnetic stand for 10 minutes. The supernatant was removed and the SPRI beads were washed twice with fresh 80% ethanol (30 seconds each wash) and air-dried for 2-3 minutes. Library DNA was eluted from the SPRI beads with a 20 µL volume of 10 mM Tris-HCl (pH 8.5). Beads were incubated at room temperature for 10 minutes, then the tubes were transferred to a magnetic stand for 10 minutes. The supernatant containing the DNA libraries was carefully removed by pipetting and transferred into a clean microcentrifuge tube. The individual libraries were quantified by Thermo Fisher Qubit [Thermo Fisher Qubit fluorimeter, RRID:SCR_018095] fluorometric quantification and quality checked by Agilent Bioanalyzer 2100 [Agilent 2100 Bioanalyzer Instrument, RRID:SCR_019389] analysis, respectively. Individual libraries were pooled into one tube, purified over a Qiagen MinElute column [QIAGEN, RRID:SCR_008539], eluted in 20 µL of 10 mM Tris, (pH 8.5) and sequenced on a NextSeq 500 next generation sequencer (Illumina NextSeq 500, RRID:SCR_014983) utilizing a 150 cycle high-output kit (Illumina, San Diego, CA). Raw sequencing data was mapped using TopHat2 (5) [TopHat, RRID:SCR_013035] onto the human genome build UCSC hg38 (NCBI 51) for human data and log2-fold changes were calculated with diffReps (6) [diffReps, RRID:SCR_010873]. Sequence tracks were visualized with IGV software [Integrative Genomics Viewer, RRID:SCR_011793] (7, 8). To identify super enhancers, we performed a ranked order of super enhancers (ROSE) analysis [ROSE, RRID:SCR_017390] utilizing the H3K27Ac status of the chromatin according to the methods of Loven et al. (10). Analysis of transcription factor binding motifs in gained ATAC-Seq and ChIP-Seq peaks was performed with HOMER [HOMER, RRID:SCR_010881] (11).

**Transcriptome Analysis.** Total RNA was isolated from untreated or Menin inhibitor-treated AML and sAML cells utilizing a PureLink RNA Mini kit from Ambion, Inc. (Austin, TX). Sequencing libraries were prepared with ERCC spike-in controls in the MD Anderson Cancer Center DNA Sequencing and Microarray core facility and sequenced on an Illumina HiSeq-4000 next generation sequencer [Illumina HiSeq 3000/HiSeq 4000 System, RRID:SCR_016386]. Each library yielded 30-40 million read pairs. Data was mapped using TopHat2 [RRID: SCR_013035] (5) onto the human genome build UCSC hg38 (NCBI 51) for human data. Gene expression was assessed using Cufflinks2 (12) [Cufflinks, RRID: SCR_014597], then variance stabilization and quantile normalization were applied. Significantly altered transcripts were determined using the limma package (13) in R [LIMMA, RRID: SCR_010943]; multiple hypotheses testing correction was applied using the false discovery rate (fdr) method implemented in the R statistical system. We considered that significance was achieved for fold changes greater than or equal to 1.25X up or down relative to the untreated or parental cells, and p-values less than 0.05. We inferred enriched pathways using the Gene Set Enrichment (GSEA) method (14), and the gene set collection from the Molecular Signature Database (MSigDB) (15) [Molecular Signatures Database, RRID:SCR_016863].

**Single cell ATAC and RNA-Seq.** PD MLL-AF9 + FLT3-TKD AML#4 cells were treated with 500 nM of SNDX-50469 (2 million cells per condition) for 16 hrs and then cryopreserved in freezing media (90% FBS + 10% DMSO) until processing of sample. For single cell ATAC Seq analysis, the AML sample (~6000 cells sampled) was processed according to the manufacturer, 10x Genomics, recommendation for scATAC-Seq utilizing a Chromium Next GEM Single Cell ATAC Library & Gel Bead Kit v1.1, Chromium Next GEM Chip H Single Cell Kit and Single Index Kit N Set A. For single cell RNA-Seq, the AML sample (~6000 cells sampled) was processed according to the manufacturer, 10x Genomics, recommendation for scRNA-Seq (Fresh Frozen Human PBMCs for Single Cell RNA Sequencing and Chromium Next GEM Single Cell 3’ Reagent Kits v3.1 User Guide). Indexed ATAC or cDNA libraries were sequenced on an Illumina NovaSeq 6000 Sequencing System [RRID:SCR_020150]. ATAC and RNA data were mapped onto the human genome build UCSC hg38 (NCBI 51) for human data. Cell clusters were defined by Cell Ranger ATAC [RRID:SCR_021160] or Cell Ranger [RRID:SCR_017344] and imaged by Loupe Browser 5.0 [RRID:SCR_018555]. Composition of cells within each sc-RNA cluster was determined by the SingleR algorithm (16). Differential gene expressions were determined as greater than 1.25-fold up or down and a p-value less than 0.05. Gene set enrichments of scRNA-Seq data were performed by GSVA analysis (17).

**Cell proliferation analysis.** For cell proliferation analysis following Menin knockout, cells were plated in duplicate or triplicate at 0.125 x 10^6^ cells/mL and total cell numbers were counted utilizing a Countess-2 cell counting instrument (Life Technologies, Carlsbad, CA) at the end of 5 days of incubation.

**Assessment of leukemia cell differentiation.** Following treatment with Menin inhibitors, cells were harvested and washed with 1X PBS. Cells were re-suspended in 0.5% BSA/PBS and stained with APC-conjugated anti-CD11b antibody [RRID:AB_398456] or APC-conjugated IgG1 isotype control antibody [RRID:AB_398613] in the dark, at 4°C for 15-20 minutes. Cells were washed with 0.5% BSA/PBS by centrifugation at 125 x g for 5 minutes, and then re-suspended in 0.5% BSA/PBS for analysis by flow cytometry. Cells were assessed in the FL-4 fluorescence channels on a BD Accuri CFLow6 flow cytometer. Differentiation of leukemia cells was also determined by examination of cellular/nuclear morphology. Cells were cytospun onto glass slides at 500 rpm for 5 minutes. The cytospun cells were fixed and stained with a Protocol® HEMA3 stain set (Fisher Scientific, Kalamazoo, MI). Cellular/nuclear morphology was assessed by light microscopy. Two hundred cells were counted in at least 5 sections of the slide for each condition. The % morphologic differentiation is reported relative to control cells. Each experiment was performed at least twice.

**Assessment of apoptosis by annexin-V staining.** Following designated treatments, untreated or drug-treated cells were stained with Annexin-V (Pharmingen, San Diego, CA) and TO-PRO-3iodide (Life Technologies, Carlsbad, CA) and the percentages of annexin V-positive, apoptotic cells were determined by flow cytometry on a BD Accuri CFlow-6 flow cytometer (BD Biosciences, San Jose, CA).

**Assessment of percentage non-viable cells.** Following designated treatments (72-96 hours), cultured cell lines or patient-derived (PD) AML blast cells, were washed with 1X PBS, stained with TO-PRO-3 iodide (Cat# T3605, Life Technologies, Carlsbad, CA) and analyzed by flow cytometry on a BD Accuri CFlow-6 flow cytometer (BD Biosciences, San Jose, CA). We used matrix dosing of agents in combinations to allow synergy assessment utilizing the SynergyFinder V2 online web application tool (<http://synergyfinder.fimm.fi/>) and Delta Synergy scores by ZIP method (18-20). For primary AML cells treated with SNDX50469-based combinations we also determined the combination index (CI) values utilizing CompuSyn (Chou and Talalay method). Combination index values less than 1.0 indicate a synergistic interaction of the two drugs in the combination.

**RNA isolation and quantitative polymerase chain reaction.** Following the designated treatments, total RNA was isolated from cultured or patient-derived AML cells utilizing a PureLink RNA Mini kit from Ambion, Inc. (Austin, TX) and reverse transcribed with a High Capacity Reverse Transcription kit from Life Technologies (Carlsbad, CA). Quantitative real-time PCR analysis for the expression of target genes was performed on cDNA using TaqMan probes and a TaqMan Universal PCR Mastermix from Applied Biosystems (Foster City, CA). Relative mRNA expression was normalized to the expression of GAPDH and compared to the untreated cells.

**Cell lysis and protein quantitation.** Untreated or drug-treated cells were centrifuged, and the cell pellets were incubated in lysis buffer on ice for 20 minutes (21). After centrifugation, an aliquot of each cell lysate was diluted 1:10 and the protein content was quantitated using a BCA protein quantitation kit (Pierce, Rockford, IL), according to the manufacturer’s protocol. Protein concentrations were determined by comparing the absorbance at 562 nm compared to a known concentration range of bovine serum albumin (BSA) from 0.125 mg to 2 mg/mL.

**SDS-PAGE and immunoblot analyses.** Seventy-five micrograms of total cell lysate were used for SDS-PAGE. Western blot analyses were performed on total cell lysates using specific antisera or monoclonal antibodies. Blots were washed with 1× PBST, then incubated in IRDye 680RD goat anti-mouse (RRID:AB_10956588) or IRDye 800CW goat anti-rabbit (RRID:AB_621843) secondary antibodies (LI-COR, Lincoln, NE) for 1 h, washed three times in 1× Phosphate Buffered Saline with Tween®20 (PBST) and scanned with an Odyssey CLX Infrared Imaging System utilizing Image Studio 5.0 Software (RRID:SCR_015795) (LI-COR, Lincoln, NE). The expression levels of β-Actin or GAPDH in the cell lysates were used as the loading control for the western blots. Immunoblot analyses were performed at least twice. Representative immunoblots were subjected to densitometry analysis. Densitometry analysis was performed using ImageJ software (22) [Image J: RRID:SCR_003070).

**Single cell next-generation mass cytometry ‘CyTOF’ analysis of mtNPM1 + FLT3-TKD-expressing AML cells.** Primary, patient-derived mtNPM1 + FLT3-TKD-expressing AML cells were treated with 1.0 µM of SNDX-50469 alone or in combination with 500 nM of OTX015 for 16 hours. At the end of treatment, cells were blocked with staining buffer (0.5% BSA/PBS) for 30 minutes, then a cocktail of extracellular antibodies (CLEC12A, CD123, CD244, CD99, CD33 and CD11b) conjugated to transition element isotopes were added and incubated for 1 hour at room temperature (RT). For viability staining, a 5 µM concentration of cisplatin was added and incubated at RT for 2 minutes. Cells were washed with staining buffer, centrifuged at 500 x g for 5 minutes and staining buffer was vacuum aspirated. Cells were fixed with 100 µL of 1.6% paraformaldehyde (PFA) for 10 minutes at room temperature. Following this, cells were permeabilized with 900 µL of ice-cold 100% methanol (90% volume) at -20°C for at least 20 minutes. Next, cells were washed with 1 ml of staining buffer to remove the paraformaldehyde/methanol solution. Cells were blocked in 50 µL of staining buffer for 30 minutes and a cocktail of intracellular antibodies conjugated to transition element isotopes was added to be used as tags in atomic mass spectrometric analysis of the cells. Cells were incubated for 1 hour at room temperature, then washed with staining buffer at 500 x g for 5 minutes. Intercalator was added (500 µL of 1:1000 Ir-intercalator diluted in 1.6% PFA/1X PBS) and cells were incubated overnight at 4°C. Cells were washed twice (500 x g for 5 minutes per wash) in staining buffer, then counted using a Countess II counting device. Following the last wash, 1 x 10^6^ cells were suspended in 100 µL of de-ionized water and incubated overnight at 4°C. Time-of-flight mass spectrometry (CyTOF) measured multiple different cellular parameters simultaneously in each cell. The absolute fold-change of protein expression changes in SNDX-50469 and OTX015-treated cells over control cells within the CLEC12A Hi, CD123 Hi, CD99 Hi, CD33 Hi, CD11b Lo population was analyzed by the Astrolabe Cytometry Platform (Astrolabe, Fort Lee, NJ).

**Reverse phase protein array (RPPA) analysis.** Menin inhibitor and BET inhibitor-treated cells were treated in biologic triplicates for 24 to 48 hours. At the end of treatment, cells were harvested, washed once with 1X PBS and snap frozen in liquid nitrogen. RPPA analysis was performed in the Functional Proteomics RPPA core facility at the MD Anderson Cancer Center. This array allows the simultaneous detection of 494 unique antibodies against human proteins. This array is curated and highly validated. Briefly, cell lysates were serially diluted two-fold for 5 dilutions (from undiluted to 1:16 dilution) and arrayed on nitrocellulose-coated slides in an 11 x 11 format. Samples were probed with antibodies by tyramide-based signal amplification approach and visualized by DAB colorimetric reaction. Slides were scanned on a flatbed scanner to produce 16-bit tiff image. Spots from tiff images were identified and the density was quantified by Array-Pro Analyzer. Relative protein expression for each sample were normalized by interpolation of each dilution curves from the "standard curve" (supercurve) of the slide (antibody). Supercurve is constructed by a script in R, written by the Bioinformatics Department at the University of Texas MD Anderson Cancer Center (23). These values (given as Log2 values) are defined as Supercurve Log2 (Raw) values and imported into an Excel worksheet. All the data points were normalized for protein loading and transformed to linear value, designated as "Normalized Linear" (labeled "NormLinear" in the worksheet). "Normalized Linear" values were transformed to Log2 values (labeled "NormLog2" in worksheet), and then median-centered for hierarchical clustering analysis (labeled "NormLog2_MedianCentered" in the worksheet). Median-centered values were then formatted for heatmap generation in the "Format for Heatmap" worksheet. Our data were further processed and our heatmaps display only proteins that were altered greater than or equal to 25% up or down and had a p-value of less than 0.05. Multiple hypotheses testing correction was applied using the false discovery rate (fdr) method as implemented in the R statistical system.

**CRISPR domain-scanning dropout screen in MOLM13 and MV4-11 cells.** To determine specific dependencies in AML cells and identify sgRNA dropouts that were synthetically lethal with with Menin inhibitor treatment, we obtained a human chromatin regulatory domain-focused CRISPR screening library, in which sgRNAs had been designed to target the catalytic domain or bromodomain of each protein in the library based on the NCBI database annotation, from the laboratory of Dr. Christopher Vakoc (Cold Spring Harbor Laboratory, Cold Spring Harbor, New York) (24). The library of sgRNAs was sub-cloned into E. coli Stbl3 cells. Lentivirus was prepared as described above. MOLM13 and MV4-11-Cas9-expressing cells were transduced with the library in biologic duplicates for 24 hours. Cells were washed, centrifuged at 125 x g, and plated in complete media. Forty-eight hours post-transduction, a portion of the cells were removed and frozen for genomic DNA isolation and PCR amplification. The remaining cells were cultured for an additional 6 days. On day 8, cells were split into treatment groups (in biologic duplicates) and treated for 96 hours with Menin inhibitor. MOLM13 cells were treated with 500 nM of SNDX-50469. Twelve days post-transduction, live AML cells were harvested by ficoll density centrifugation, and genomic DNA was isolated with a GeneJET Genomic DNA Purification Kit (Thermo Scientific). Primers surrounding the sgRNA sequence were utilized to minimally amplify (20 cycles) the genomic DNA (100 ng per reaction in 16-20 separate PCR reactions) from the Day2-transduced cells and the Day12 untreated and SNDX-50469-treated cells. Sequencing libraries were constructed and amplicon-seq was performed on a NextSeq 500 next generation sequencer (Illumina NextSeq 500, RRID:SCR_014983) using a 150 cycle mid-output kit (Illumina, San Diego, CA). Sequencing reads were used to determine the read counts of the remaining sgRNAs in each condition. Log2 fold-changes in the sgRNA reads between Day12 and Day2 or between Day12 treated and untreated groups were calculated utilizing the CRISPRCloud2 online software application (25). Only dropouts or enrichments of sgRNAs that were significant (p< 0.05 and f.d.r. < 0.05) compared to the respective Day2 or Day 12 Control and in the same direction (up or down) in both replicates were graphed with GraphPad V9 [GraphPad Prism: RRID:SCR_002798] software .

**In vivo models of de novo AML.** All in vivo studies were approved by and conducted in accordance with the guidelines of the IACUC at the M.D. Anderson Cancer Center, an AAALAC-accredited facility. Male and female NOD.Cg-Prkdc^scid^ Il2rg^tm1Wjl^/SzJ (NSG) mice (stock number: 005557; 4-6 weeks of age) [Jackson Labs, Bar Harbor, ME; RRID: IMSR_JAX:005557] were exposed to 2.5 Gy of radiation. The following day, mice (n=10 per cohort) were injected in the lateral tail vein with 0.3 x 10^6^ GFP-luciferase expressing MOLM13 or 3.0 x 10^6^ luciferase-GFP expressing AML PDX cells (Dana Farber PDX number: DF87153) (26) and monitored for 4-5 days. Mice were imaged utilizing a Xenogen IVIS Lumina in vivo imaging system to document engraftment before treatment was initiated. Mice were randomized into groups based on equivalent mean bioluminescent intensity to control for variation in cell engraftment and variation between different treatment groups. Treatments were initiated on day 4-5. For the MOLM13 model, mice were treated with Vehicle, SNDX-50469 (30 mg/kg, daily x 5 days, by oral gavage) and/or OTX015 (30 mg/kg, daily x 5 days, by oral gavage) for 2 weeks. SNDX-50469 was reconstituted in a solution of 10% (vol/vol) DMSO, 90%(vol/vol) of 20% Captisol (sulfobutylether-β-cyclodextrin) ( formulated in sterile, deionized water) (Ligand, San Diego, CA). OTX015 was prepared in a solution of 10% (vol/vol) of 95% ethanol, followed by 30% (vol/vol) of PEG-400 (ThermoFisher Scientific, Waltham, MA), and then 60% (vol/vol) of Phosal-50 (LIPOID, LLC via ThermoFisher Scientific, Waltham, MA). Mice were imaged weekly by bioluminescent imaging to document treatment efficacy and/or disease progression. Total bioluminescence was recorded as photons/second. Mice that became moribund or experienced hind limb paralysis were euthanized according to the approved IACUC protocol. Department of Veterinary Medicine staff members assisting in determining when euthanasia was required were blinded to the experimental conditions of the study. The survival of the mice is represented by a Kaplan-Meier plot. Significance was determined by a Mantel-Cox log rank test. P-values of less than 0.05 were assigned significance. To further assess the in vivo combination of Menin inhibitor and OTX015, mice (n=10 per cohort) were injected by lateral tail vein with 2.5 x 10^6^ MLL-AF9 + FLT3-TKD Luc/GFP AML PDX cells (Dana Farber PDX number: DF68555) (26) and monitored for 5 days. Mice were imaged utilizing a Xenogen IVIS Lumina in vivo imaging system to document engraftment before treatment was initiated. Mice were randomized into groups based on equivalent mean bioluminescent intensity to control for variation in cell engraftment and variation between different treatment groups. Treatments were initiated on day 5. For the MLL-AF9 + FLT3-TKD Luc/GFP AML PDX mouse model, mice were treated with vehicle (0.5% methylcellulose solution (in water)), SNDX5613 (50 mg/kg, B.I.D., daily x 5 days, by oral gavage) and/or OTX015 (30 mg/kg, daily x 5 days, by oral gavage) for 6 weeks. SNDX-5613 salt was reconstituted in 0.5% methylcellulose solution. OTX015 was prepared as described above. All mice in each treatment cohort were imaged utilizing a Xenogen IVIS Lumina in vivo imaging system once per week to monitor disease status and treatment efficacy. Total bioluminescence was recorded as photons/second. Mice that became moribund or experienced hind limb paralysis were euthanized according to the approved IACUC protocol. Department of Veterinary Medicine staff members assisting in determining when euthanasia was required were blinded to the experimental conditions of the study. The survival of the mice is represented by a Kaplan-Meier plot. Significance was determined by a Mantel-Cox log rank test. P-values of less than 0.05 were assigned significance. To determine the in vivo efficacy of the combination of SNDX-5613 and GNE-781, mice (n=10 per cohort) were injected in the lateral tail vein with 0.3 x 10^6^ GFP-luciferase expressing MOLM13 cells and monitored for 4 days. Mice were imaged utilizing a Xenogen IVIS Lumina in vivo imaging system to document engraftment before treatment was initiated. Mice were randomized into groups based on equivalent mean bioluminescent intensity. Treatments were initiated on day 5. Mice were treated with Vehicle (DMSO [10% (vol/vol)] + 90%(vol/vol) of 20% Captisol), SNDX5613 (50 mg/kg, B.I.D., daily x 5 days, by oral gavage) and/or GNE-781 (5 mg/kg, B.I.D., daily x 5 days, by oral gavage) for 2 weeks. GNE-781 was reconstituted in DMSO (10% vol/vol), followed by a 90% (vol/vol) of 20% Captisol solution and vortexed. All mice in each treatment cohort were imaged utilizing a Xenogen IVIS Lumina in vivo imaging system once per week to monitor disease status and treatment efficacy. Total bioluminescence was recorded as photons/second. The survival of the mice is represented by a Kaplan-Meier plot. Significance was determined by a Mantel-Cox log rank test. P-values of less than 0.05 were assigned significance.

**Power analysis for in vivo studies**. With a sample size of 10 mice per group, we can achieve 79.5% power to detect a difference of overall survival at a significance level of 0.05 with one-sided log-rank test, assuming 30% of mouse-survival at the end of study in the experimental group.

**Statistical analysis**. Significant differences between values obtained in AML cells treated with different experimental conditions compared to untreated control cells were determined using the Student’s t-test in GraphPad V9 [RRID:SCR_002798]. For the *in vivo* mouse models, a two-tailed, unpaired t-test was utilized for comparing total bioluminescent flux. For survival analysis, a Kaplan-Meier plot and a Mantel–Cox log rank test were utilized for comparisons of different cohorts. P values of < 0.05 were assigned significance.

**Data and Software availability**. ATAC-Seq, sc-ATAC-Seq, ChIP-Seq, bulk RNA-Seq and sc-RNA-Seq datasets have been deposited in GEO under Accession ID: GSE190719 and GSE228326.

**REFERENCES for Supplemental Methods**

1. Labun K, et al. CHOPCHOP v2: a web tool for the next generation of CRISPR genome engineering. Nucleic Acids Res. 2016; 44: W272-6.
2. Nabet B, et al. The dTAG system for immediate and target-specific protein degradation. Nat Chem Biol. 2018; 14: 431-441.
3. Khan M, et al. Clinical outcomes and co-occurring mutations in patients with runx1-mutated acute myeloid leukemia. Int J Mol Sci. 2017; 18:1618
4. Buenrostro JD, Wu B, Chang HY, Greenleaf WJ. ATAC-seq: A method for assaying chromatin accessibility genome-wide. Curr Prot Mol Biol. 2015; 109: 21-29.
5. Kim D, et al. TopHat2: accurate alignment of transcriptomes in the presence of insertions, deletions and gene fusions. Genome Biol. 2013; 14: R36.
6. Shen L, et al. diffReps: detecting differential chromatin modification sites from ChIP-seq data with biological replicates. PLoS One. 2013; 8: e65598.
7. Robinson JT, et al. Integrative genomics viewer. Nat Biotechnol 29, 24-6, (2011).
8. Thorvaldsdottir H, Robinson JT, Mesirov JP. Integrative Genomics Viewer (IGV): high-performance genomics data visualization and exploration. Briefings in Bioinformatics. 2013; 14: 178-92.
9. Schmidl C, Rendeiro AF, Sheffield NC, Bock C. ChIPmentation: fast, robust, low-input ChIP-seq for histones and transcription factors. Nat Methods. 2015; 12: 963-5.
10. Loven J, et al. Selective inhibition of tumor oncogenes by disruption of super-enhancers. Cell. 2013; 153: 320-34.
11. Heinz S, et al. Simple Combinations of Lineage-Determining Transcription Factors Prime cis-Regulatory Elements Required for Macrophage and B Cell Identities. Mol Cell 2010; 38: 576-589.
12. Trapnell C, et al. Transcript assembly and quantification by RNA-Seq reveals unannotated transcripts and isoform switching during cell differentiation. Nat Biotechnol 28, 511-5, (2010).
13. Smyth GK. Linear models and empirical bayes methods for assessing differential expression in microarray experiments. Statistical applications in genetics and molecular biology 3, Article3, (2004).
14. Subramanian A, et al. Gene set enrichment analysis: a knowledge-based approach for interpreting genome-wide expression profiles. Proc Natl Acad Sci USA 102, 15545-50, (2005).
15. Liberzon A, et al. Molecular signatures database (MSigDB) 3.0. Bioinformatics. 2011; 27: 1739-40.
16. Aran D, et al. Reference-based analysis of lung single-cell sequencing reveals a transitional profibrotic macrophage. Nat. Immunol. 2019; 20:163-172.
17. Hänzelmann S, Castelo R, Guinney J. GSVA: gene set variation analysis for microarray and RNA-seq data. BMC Bioinformatics. 2013; 14:7.
18. Yadav B, Wennerberg K, Aittokallio T, Tang J. Searching for Drug Synergy in Complex Dose-Response Landscapes Using an Interaction Potency Model. Comput Struct Biotechnol J 2015; 13: 504-13.
19. Ianevski A, He L, Aittokallio T, Tang J. SynergyFinder: a web application for analyzing drug combination dose-response matrix data. Bioinformatics. 2017; 33: 2413-2415.
20. Ianevski, A., Giri, A. K., and Aittokallio, T. SynergyFinder 2.0: visual analytics of multi-drug combination synergies, Nucleic Acids Res. 2020; 48: W488-W493.
21. Fiskus W, Verstovsek S, Manshouri T, Rao R, Balusu R, Venkannagari S, et al. Heat shock protein 90 inhibitor is synergistic with JAK2 inhibitor and overcomes resistance to JAK2-TKI in human myeloproliferative neoplasm cells. Clin Cancer Res. 2011; 17: 7347-58.
22. Schneider CA, Rasband WS, Eliceiri KW. NIH Image to ImageJ: 25 years of image analysis. Nat Methods. 2012; 9: 671-5.
23. Troncale S, et al. NormaCurve: a SuperCurve-based method that simultaneously quantifies and normalizes reverse phase protein array data. PLoS One. 2012; 7: e38686.
24. Shi J, et al. Discovery of cancer drug targets by CRISPR-Cas9 screening of protein domains. Nat Biotechnol. 2015; 33: 661-7.
25. Jeong HH, Kim SY, Rousseaux MWC, Zoghbi HY, Liu Z. CRISPRcloud: a secure cloud-based pipeline for CRISPR pooled screen deconvolution. Bioinformatics. 2017; 33: 2963-2965.
26. Townsend EC, et al. The Public Repository of Xenografts Enables Discovery and Randomized Phase II-like Trials in Mice. Cancer Cell. 2016; 29: 574-586.
